# Scanning the solutions for the sustainable supply of forest ecosystem services in Europe

**DOI:** 10.1007/s11625-022-01111-4

**Published:** 2022-03-22

**Authors:** M. Hernández-Morcillo, M. Torralba, T. Baiges, A. Bernasconi, G. Bottaro, S. Brogaard, F. Bussola, E. Díaz-Varela, D. Geneletti, C. M. Grossmann, J. Kister, M. Klingler, L. Loft, M. Lovric, C. Mann, N. Pipart, J. V. Roces-Díaz, S. Sorge, M. Tiebel, L. Tyrväinen, E. Varela, G. Winkel, T. Plieninger

**Affiliations:** 1 Eberswalde University for Sustainable Development, Sustainable Forest Resource Economics, Schicklerstrasse 5, 16225 Eberswalde, Germany; 2 Faculty of Organic Agricultural Sciences, University of Kassel, Steinstraße 19, 37213 Witzenhausen, Germany; 3 Department of Agricultural Economics and Rural Development, University of Göttingen, Platz der Göttinger Sieben 5, 37073 Göttingen, Germany; 4 Centre de Propietat Forestal (CPF), Santa Perpètua de Mogoda, 08130 Barcelona, Spain; 5 Pan Bern AG, Hirschengraben 24, 3001, Bern, Switzerland; 6 Land Environment Agriculture and Forestry Department (TeSAF), University of Padova, Viale dell’Università 16, Legnaro, 35020 Padova, Italy; 7 Lund University Centre for Sustainability Studies Lund University, Box 170, 221 00 Lund, Sweden; 8 Forest Service of the Autonomous Province of Trento, via Trener 3, 38121 Trento, Italy; 9 Research Group COMPASSES-Planning and Management in Social-Ecological Complex Adaptive Systems University of Santiago de Compostela. Campus Universitario, s/n 27002, Lugo, Spain; 10 Department of Civil, Environmental and Mechanical Engineering, University of Trento, via Mesiano 77, Trento, Italy; 11 Forest Research Institute Baden-Wuerttemberg (FVA), Wonnhaldestrasse 4, 79100 Freiburg, Germany; 12 Department of Geography, University of Innsbruck, Innrain 52f, 6020 Innsbruck, Austria; 13 University of Natural Resources and Life Sciences Vienna, Institute for Sustainable Economic Development, Feistmantelstraße 4, 1180 Vienna, Austria; 14 Working Group Governance of Ecosystem Services, Leibniz Centre for Agricultural Landscape Research (ZALF), Eberswalder Str. 84, 15374 Müncheberg, Germany; 15 European Forest Institute Yliopistokatu 6B, 80100 Joensuu, Finland; 16 KU Leuven, Department of Earth and Environmental Sciences Celestijnenlaan 200E, 3001 Leuven, Belgium; 17 Centre for Ecological Research and Forestry Applications (CREAF), 08193 Cerdanyola del Valles, Spain; 18 Department of Agricultural Economics and Rural Development, University of Göttingen, Platz der Göttinger Sieben 5, 37073 Göttingen, Germany; 19 Natural Resources Institute Finland, Latokartanonkaari 9, 00790 Helsinki, Finland; 20 Forest Science and Technology Centre of Catalonia, Ctra. St. Llorenç de Morunys, 25280 Solsona, Spain; 21 Forest and Nature Conservation Policy Group, Wageningen University, Droevendaalsesteeg 3, 6700 AA Wageningen, The Netherlands

**Keywords:** European forests, Ecosystem services, Sustainability, Solution scanning, Leverage points, EU Forestry Strategy

## Abstract

Forests are key components of European multifunctional landscapes and supply numerous forest ecosystem services (FES) fundamental to human well-being. The sustainable provision of FES has the potential to provide responses to major societal challenges, such as climate change, biodiversity loss, or rural development. To identify suitable strategies for the future sustenance of FES, we performed a solution scanning exercise with a group of transdisciplinary forest and FES experts from different European regions. We identified and prioritized fifteen major challenges hindering the balanced provision of multiple FES and identified a series of potential solutions to tackle each of them. The most prominent challenges referred to the increased frequency and impacts of extreme weather events and the normative mindset regarding forest management. The respective solutions pointed to the promotion of forest resilience via climate-smart forestry and mainstreaming FES-oriented management through a threefold strategy focusing on education, awareness raising, and networking. In a subsequent survey, most solutions were assessed as highly effective, transferable, monitorable, and with potential for being economically efficient. The implementation of the solutions could have synergistic effects when applying the notion of leverage points. Seven emerging pathways towards the sustainable supply of FES have been identified. These pathways build on each other and are organized based on their potential for transformation: (1) shifting forest management paradigms towards pluralistic ecosystem valuation; (2) using integrated landscape approaches; (3) increasing forest resilience; (4) coordinating actions between forest-related actors; (5) increasing participation in forest planning and management; (6) continuous, open, and transparent knowledge integration; and (7) using incentive-based instruments to support regulating and cultural FES. These pathways can contribute to the implementation of the new EU Forestry Strategy to support the balanced supply of multiple FES.

**Graphical abstract:**

**Supplementary Information:**

The online version contains supplementary material available at 10.1007/s11625-022-01111-4.

**Figure:**
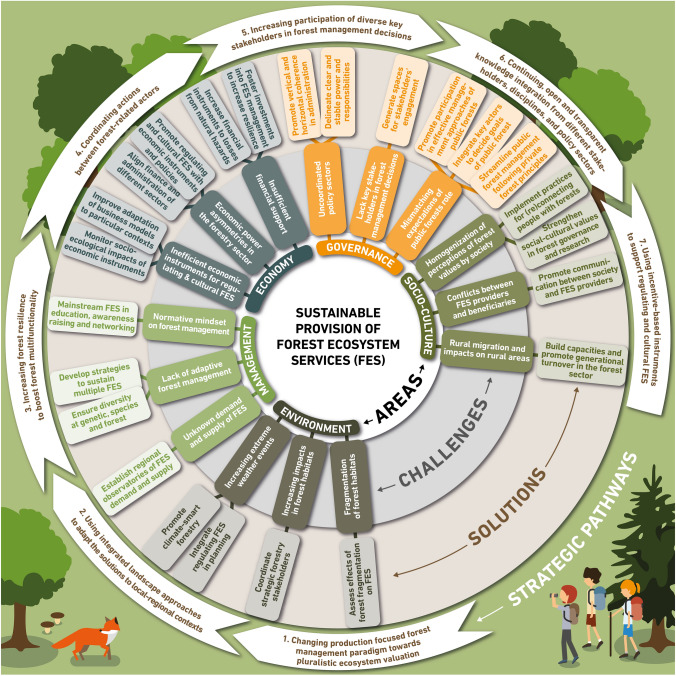

## Introduction

European forests are ecosystems that deliver manifold benefits to society via so-called Forest Ecosystem Services (FES) (Orsi et al. 2020). The benefits that people obtain from the forests, so-called Forest Ecosystem (FES) (MEA 2005), include for example carbon sequestration, protection of soils and water basins, provision income opportunities, physical and mental health benefits, and contribution to the general well-being of rural and urban inhabitants. Furthermore, forests provide renewable resources that offer alternatives to fossil fuel-based products, thus contributing to climate change mitigation (Forest Europe 2020). However, at the same time, numerous direct and indirect drivers of change increasingly challenge the resilience of forests and the provision of FES. These include, for example, climate change, which threatens almost 60% of European forests by increasing their vulnerability to windstorms, fires, and pest infestations (Forzieri et al. 2021), and diverging societal demands ranging from an increased production of wood or biofuel to the promotion of wilderness for recreational purposes (EEA 2016).

To navigate these challenges, it is imperative that forests are sustainably managed so they can continue being part of the solution to mitigate climate change, biodiversity loss, or to control epidemic outbreaks (Swaddle and Calos 2008; Khalil et al. 2016), while maintaining a crucial role in the efforts towards a more sustainable society and economy in Europe (Wolfslehner et al. 2020). Sustainable management is at the core of the European Union’s (EU) forest policy (EC 2013). The previous EU Forest Strategy already highlighted the importance of “balancing various forest functions, meeting demands, and delivering vital ecosystem services”. It called for supporting protection and management efforts aimed at maintaining, enhancing, and restoring the resilience and multi-functionality of forest ecosystems for both urban and rural areas (EC 2013). Various studies have highlighted the importance of multifunctional management for safeguarding different forest functions (Wolf and Primmer 2006; Gustafsson et al. 2012; Benz et al. 2020). In addition, forest products and services are increasingly an inherent and integrated element of many other policy sectors, ranging from energy to food production, conservation and public health (Aznar-Sánchez et al. 2018).

Yet, there is a mismatch between the supply of FES and their recognition in policies across Europe inducing a bias towards timber provision (Primmer et al. 2021).

Strategies for a broad supply of FES often entail competing objectives (Lazdinis et al. 2019). Besides, the disproportionate focus on biomass production, especially in large parts of central and northern Europe, hinder the potential development of other FES. The conflicting demands can be due to the fact that most actions affecting forest landscapes are primarily associated with policy areas and interests outside the forest sector. As a result, some forest objectives are torn between different sectoral interests whenever new targets evolve outside the forest sector (Sotirov et al. 2016).

To address the current sustainability challenges, European forests demand innovative solutions for which the new EU policy frameworks, such as the EU Green Deal and Forestry Strategy, offer emerging opportunities. To support the development and implementation of the new European Forest Strategy, it is fundamental to have clarity on the challenges hindering the sustainable provision of multiple FES and to look for effective solutions. While a plethora of information exists about the measures needed to ensure the provision of specific services such as wood or biomass, no comprehensive effort has been made to identify potential solutions to overcome the impediments in the supply of multiple FES including cultural and regulating services.

To shed light on this issue, we conducted a solution scanning exercise with experts working in different fields of science, policy, and practice in the European forestry sector. Three specific research questions were addressed in this study:

- What are the most pressing challenges hindering the sustainable provision of multiple FES in Europe?
- Which are the most effective solutions to overcome those challenges?
- How can the solutions be logically implemented so their transformational potential is maximized?

## Materials and methods

In this study, we applied an extended version of the solution scanning method. Solution scanning has been increasingly used to identify specific solutions for a particular problem (Sutherland et al. 2014). Solution scanning follows a stepwise methodology to identify a set of actions, interventions, or approaches that respond to a specific challenge. This can be useful to point out potential policy interventions in decision-making processes but also for setting research agendas (Dicks et al. 2017). First, an objective is defined. In most cases, it emerges from specific societal demands (Pullin et al. 2013). Then, a group of experts is asked to identify courses of action from their own experience that can leverage the system towards the stated goal. Finally, the proposed solutions are listed and distributed to the same experts for assessment and prioritization according to given criteria (Hernández-Morcillo et al. 2018).

**Fig. 1:**
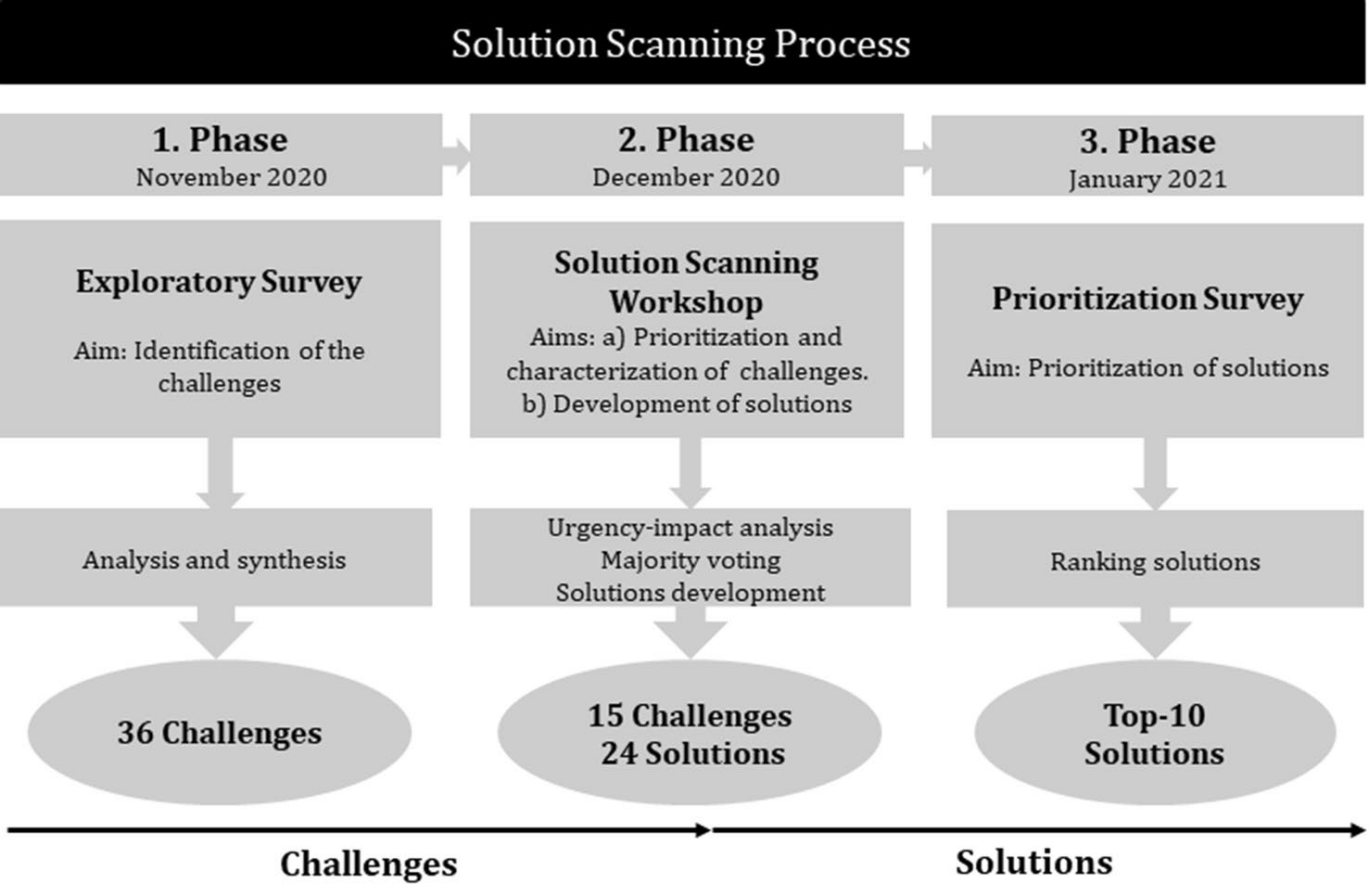
Workflow of the solution scanning exercise

Our solution scanning exercise included three phases (Fig. 1). The first phase consisted of the identification of the challenges that hamper the sustainable provision of FES in Europe. To that end, an exploratory survey was distributed in November 2020 to all expert participants of the study (S1). The survey was structured along a series of open questions, which inquired about the most pressing challenges affecting the sustainable provision of FES in Europe across five thematic areas: economy, environment, socio-culture, management, and governance. Additionally, the survey assessed key knowledge gaps hindering progress towards addressing these challenges. The proposed challenges were structured and bundled according to thematic areas, resulting in a list of 36 challenges, which was shared among all participants to identify additional uncovered challenges prior the next phase in the process.

In the second phase, a two-day participatory solution scanning workshop was organized in December 2020. The aim of the workshop was to prioritize the challenges identified in the first phase and develop strategic solutions for the most relevant ones. Based on their expertise, participants were divided into smaller groups of four–five individuals, and distributed across five thematic areas (economy, environment, socio-culture, management, and governance). On the first day, each thematic group prioritized and characterized the respective subsets of thematic challenges resulting from the exploratory survey. The prioritization included, for each challenge, a general assessment of the urgency (how immediately this challenge needs to be tackled), their impact (degree to which solving this challenge would contribute to the sustainable supply of multiple FES in Europe), the types of FES affected, scale, and the inter-relations between each of these challenges and all the thematic areas. Accordingly, each thematic group reduced the list of challenges to the five most relevant. At the end of the first day, the number of challenges was reduced through a series of anonymous majority voting rounds in plenary to the three most pressing challenges for each thematic area. During the second day, the thematic groups reconvened to formulate and characterize strategic solutions for each of the three selected challenges. The characterization consisted of a description of the solution, the feasibility of implementation, a time frame, and the resources needed for application. A detailed account of the whole process, including the definition, framing and prioritization of the challenges (first day of the workshop); and the identification and characterization of the strategic solutions (second day of the workshop) can be found in S3.

During the third phase, the identified solutions were evaluated in an online survey distributed to all participants in January 2021 (S2). The respondents rated each solution according to five different criteria of optimal solutions, adapted from the concept report on climate policy-mix optimality (Gorlach 2013) (see Box 1). Finally, the notion of leverage points understood as areas of a system where actions can be implemented to induce trasnformational changes (Abson et al. 2017; Dorninger et al. 2020) was used to organize the strategic solutions into pathways of intervention according to their potential to transform the forestry sector.
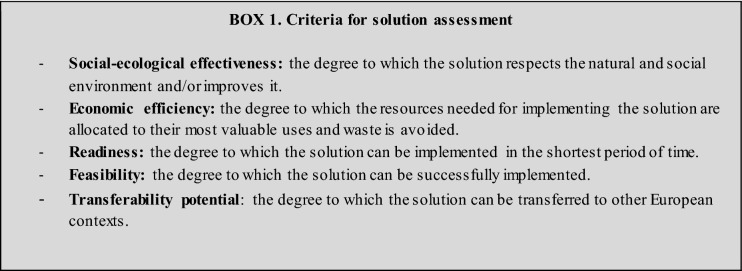


The participants in the solution scanning process were 24 experts from academia, policy, and practice working directly on FES in Europe. Most participants (90%) were related to the EU Horizon 2020 funded projects SINCERE and InnoForESt, both dealing with the promotion of FES-related innovations. A team of three facilitators selected from both projects supported the implementation of the solution scanning exercise. About expert participants, particular attention was paid to balance backgrounds between academia and practice, disciplines, geographic foci, seniority level, and gender (S4).

Most of the selected experts worked at research organizations at the interface between science, policy, and practice (41%). The covered areas of knowledge of the forestry sector were broad, including experts on forest ecosystem services governance and innovation, urban forestry and European forest policy. Partly because the coordination of both of the Horizon 2020 projects is based in Germany, most of the participants worked at German organizations (41%). However, seven other European countries were also represented in the exercise, namely Spain, Italy, Austria, Belgium, Switzerland, Finland, and Sweden. A balanced gender representation was achieved and consciously maintained along the process.

This solution scanning exercise synthesizes the currently fragmented views on forest challenges and targets suitable solutions to foster the sustainable provision of FES. The organization of the exercise into three well-defined phases allows a clear and transparent communication among the coordinators and experts, facilitating a smooth iterative process. Due to COVID-19 restrictions, the participatory process was conducted entirely online. Two important aspects of this sequential participatory method have been the regular communication with the group to ensure a shared understanding of the process, the use of preparatory materials before and after each workshop through two surveys (S1 and S2), and the presentation of the state of the work at the beginning of each phase of the solution scanning session. However, we must emphasize that the results highly depend on the participant contributions and the specific timing of the survey and workshops, which is a limitation of our approach.

## Results

### The most pressing challenges for the sustainable provision of FES

After the prioritization process, 15 challenges were selected, three per thematic area, based on their urgency and impact (Table 1).

**Table 1:** Definition of the final selected challenges for each sustainability area

Area	Challenge	Definition
Environment	*Challenge 1*. Increasing frequency and intensity of extreme weather events	Climate change results in an increase of extreme weather events regarding the frequency and intensity (e.g., storms, droughts, and rainfall) affecting the resilience of forest. It affects the susceptibility to wildfires as well as forest health, functionality, and FES provision all around Europe. Despite the inherent resilience of European forests, the resulted changes in forest structure, composition, and thus ecological functioning could be irreversible
	*Challenge 2*. Increasing extent, frequency, and impacts of pests and diseases in forest habitats	Due to climate change, forests are increasingly vulnerable to pests and diseases, as seen in the extent of recent bark beetle infestations. Especially vulnerable are forest dominated by single species stands with a higher density of trees, resulting in a lower provision of all FES at a European scale
	*Challenge 3*. Fragmentation of forest habitats	Land use change results in fragmented forest structures, habitat quality decline, and negative impacts on biodiversity. The lack of connectivity especially affects forest-dependent and endemic species. Moreover, the lack of spatial continuity could hinder the sustainable provision of FES
Management	*Challenge 4*. Narrow focus and normative mindset on forest management	Traditional and often normative mindsets on forest management are focused on timber and biomass production especially in central and north European regions. Biodiversity and FES such as cultural or regulating services could be affected by this challenge. Integrating all forest functions and socio-cultural dimensions is key for preserving healthy ecosystems, local cultures, knowledge, and values
	*Challenge 5*. Lack of adaptive forest management practices	Forests are undergoing continuous changes that demand an adaptive approach. The lack of adapted management decreases forest resilience to rapid changes affecting people and forests in specific contexts. Continuous monitoring and flexible forest management practices are challenging to implement, due to strict administrative conditions, and lack of resources and knowledge among other factors
	*Challenge 6*. Unknown demand and supply of FES	There is a lack of information on the biophysical supply and societal demand of regulating or cultural FES across European countries. Information about the FES flows, synergies, trade-offs, and bundles is missing. As a result, some services are often absent in policy discussions and decisions (e.g., cultural FES). Barriers inducing social inequality can affect the accessibility of specific FES
Economy	*Challenge 7*. Insufficient financial support for adapting to changing conditions	Support to cover losses from- and adaptation towards natural hazards are deficient to non-existent. This challenge particularly affects forest owners’ capacities to risk investing in innovations, especially when there is no guarantee of receiving sufficient revenue or at least mitigating losses. Facing periodic natural hazards without financial support often exposes forest owners to unbearable risky financial conditions
	*Challenge 8* Economic power asymmetries among actors in the European forestry sector	Power asymmetries are generally influenced by a reduced number of actors, who take decisions, control, and direct the markets. On many occasions, those actors can operate regardless of the negative externalities of intensive wood/timber production
	*Challenge 9*. Lack of efficient economic instruments and business models for regulating and cultural FES	Efficient economic instruments and business models capable of recognizing and promoting regulating and cultural FES are scarce to non-existent in Europe. This also affects non-wood forest products, particularly those of public good character. Many forest owners are motivated to provide those services, but there is a lack of economic incentives
Governance	*Challenge 10*. Lack of coordination and competition among different policy sectors	This challenge occurs across all administrative levels and policy sectors, especially those with contradicting goals affecting forest owners. As a result, making simple decisions on planning and management activities often becomes an ordeal. Depending on the policies conflicting, the process could lead to irreversible changes in the provision of specific FES
	*Challenge 11*. Lack of representation of diverse key stakeholders in forest management decision	Forest planning and management decisions are often made without considering the effects that they can have on actors beyond forest owners, managers, or policy makers. There is almost no space (vertically or horizontally) for participation of other members of the wider community of potential beneficiaries (e.g., local communities) in the decision-making process on the provision and use of FES
	*Challenge 12*. Tensions and mismatching expectations about the role of public forests	Planning and management decisions in public forests are particularly complex. Mismatching expectations about the role of public forests might emerge, seeing them as a strategic profitable resource and/or as public goods with the public mandate to provide FES
Socio-culture	*Challenge 13*. Homogenization of perceptions of forest values by society	This challenge focuses on the multiplicity of social–cultural values associated with FES as well as the difficulties in their identification, prioritization, and integration in forest planning and management. This is particularly true for the marginalized indigenous peoples, traditional communities and the associated risk with the vanishing forest-related forms of knowledge and livelihoods
	*Challenge 14*. Conflicts between FES providers and beneficiaries	The conflicts between FES providers and beneficiaries may arise due to diverging interests, demands and rights. On occasions, private owners are expected to supply a series of public goods without any incentive. This incentive is not necessarily an economic reward for the provision of FES. In occasions, the incentive is an acknowledgment or recognition. It is to some extent a communication and conceptual conflict related to the understanding of public–private relationships, power structures, and interests that regulate the use, provision, and access to forests and forest resources
	*Challenge 15*. Rural migration and impacts on rural areas	European rural areas are increasingly experiencing migratory flows to cities leading to a lack of generational turnover in the forest sector and/or abandonment of forested lands. The trend of urban dwellers moving to the countryside has not stopped the process, as less and less people engages with forest-related economic activities

**Fig. 2:**
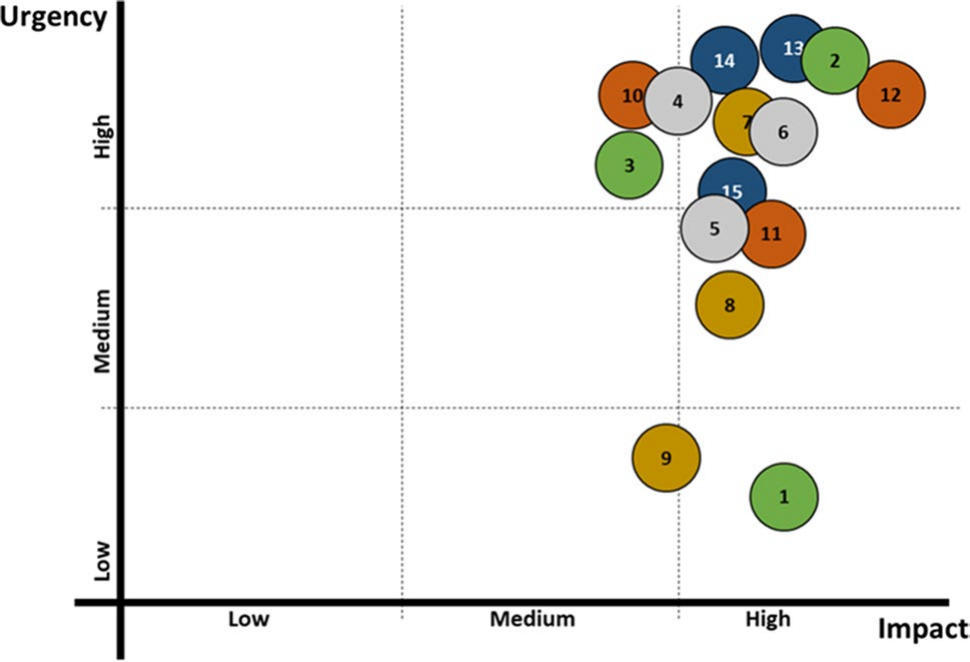
Prioritization of challenges based on urgency and impact. The colors correspond to five different areas of sustainability (green = environment, brown = socio-culture, gray = economy, blue = management, orange = governance); the numbers correspond to the challenges identified (Table 1)

Figure 2 displays the prioritization of these 15 most important challenges based on the expert group perceptions of their urgency and impact. Most of the prioritized challenges were classified as being urgent and having a high impact. The increasing areal expansion, frequency, and impacts of pests and diseases (Ch. 2), the tensions and mismatching expectations among the roles of public forests (Ch. 12), and the homogenization of perceptions of forest values by society (Ch. 13) were the challenges perceived by the experts as those that should be most immediately tackled. The resolution of these challenges would have the maximum potential to contribute to the sustainable supply of multiple FES in Europe. Challenges referring to the fragmentation of forest habitats (Ch. 3), lack of efficient economic instruments (Ch. 9), and lack of coordination among policy sectors (Ch. 10) were considered as having a medium impact due to the fact that solving these challenges would contribute to the sustainable supply of multiple FES although over a longer period of time. The increasing frequency and intensity of extreme weather events (Ch.1) was considered as the least urgent challenge, meaning that it would be occurring during a longer period, although having the biggest impact.

### The most suitable solutions to improve the sustainable provision of FES

To address the 15 challenges, 24 solutions were identified by the team of experts (see S5 for a detailed description of all the solutions). The suitability of each solution was subsequently assessed and ranked based on the following six criteria: social–ecological effectiveness, economic efficiency, readiness, feasibility, and transferability potential (Box 1). Table 2 shows the prioritized challenges per thematic area with the respective solutions and the final ranking (More detailed results on these calculations in S6). The social–ecological effectiveness, respecting the diverse contexts, and the transferability potential were the strongest traits shared by the proposed solutions. In contrast, the readiness, or the short-term implementation potential and the feasibility, understood as the potential for its successful implementation, were generally the weakest traits. After summing up the rankings of all the different criteria for all solutions, the top ten solutions were obtained. These 10 solutions are presented in detail in the next section.

**Table 2:**
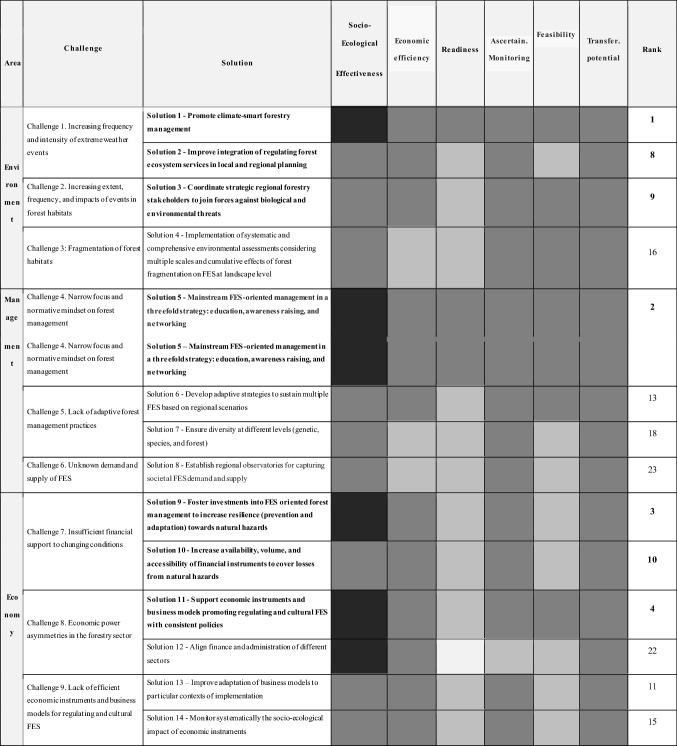

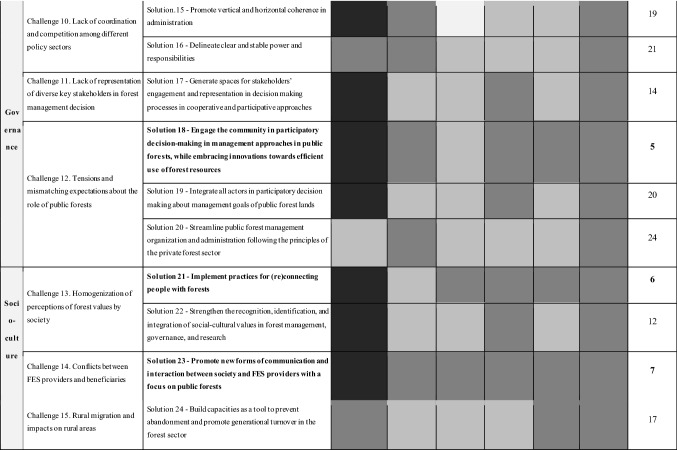
Solutions to foster sustainable FES provision in European forests

The colors indicate the degree to which each solution fulfills the implementation criteria. More detailed results on these calculations are in S6 (white = very low; light gray = low; gray = normal; dark gray = high; black = very high). Bold font indicates the ten highest ranked solutions

#### The top ten solutions for the sustainable provision of FES in Europe

##### Top 1. Promote climate-smart forestry management

*Sustainability Area: Environment; Challenge 1—Increasing frequency and intensity of extreme weather events*

Climate-smart forestry is a targeted approach to manage forests in response to climate change (Bowditch et al. 2020). It aims to increase the climate regulation benefits from forests and the forest sector, in a way that creates synergies addressing other societal needs related to forests while increases forest resilience. It is a large-scale strategy, which includes three main lines of action: the enhancement of natural regeneration and avoidance of deforestation; active forest management; and adaptive forest management to build resilient forests (Nabuurs et al. 2018; Verkerk et al. 2020). For example, a recent analysis along a climate gradient across Europe showed that mixed forest, particularly those forest mixing conifer and broadleaved stands, are more resilient and resistant to drought events than monospecific forests (Pardos et al. 2021). Here, forest resilience refers to the maintenance of regimes and the adaptive capacity of forests as a coupled human–natural system in the face of drivers of change (Nikinmaa et al. 2020). As such, climate-smart forestry strives beyond storing carbon to mitigate climate change and generate synergies with multiple FES and biodiversity. The implementation of this solution needs to carefully consider the different regional contexts in Europe to identify the most cost-effective management options. It would also require sustained commitment as the benefits from this solution would only emerge in a mid-long term.

##### Top 2. Mainstream FES-oriented management in a threefold strategy: education, awareness raising, and networking

*Sustainability Area: Management; Challenge 4—Narrow focus and normative mindset on forest management*

This solution invites broadening the often-narrow perspective of forest management focused on the timber and biomass production of highly productive stands (Jönsson and Snäll 2020), with the help of education and information strategies. In particular, this could be done by diversifying education at the administration and university level (Nair 2004), fostering knowledge transfer to forest operators (Perera et al. 2006), starting and reinforcing social campaigns to make visible the multiple services of forest, and developing and enabling long-lasting cross-sectorial networks (Guerrero and Hansen 2021). Although this solution requires long-term commitment and significant attitudinal change within and beyond the forestry sector (shifting management goals, seeking long-term instead of short-term benefits, or changing contractual arrangements) before its effects become apparent, this solution has the potential to largely generate synergistic and long-lasting effects over forest management in Europe. To tackle complex challenges and developing opportunities for innovation at EU level, collaboration can be enhanced through existing European Innovations Partnership (EIP) operational groups on forest and EU projects through multi-actor approaches. Moreover, in the light of the new EU CAP, Agricultural Knowledge, and Innovation Systems (AKIS) are key to support more intensely the sharing of knowledge and innovation.

##### Top 3. Foster investments into FES-oriented forest management to increase resilience (prevention and adaptation measures) towards natural hazards.

*Sustainability Area: Economy; Challenge 7—Insufficient financial support to changing conditions*

Investing in increasing forest resilience (Nikinmaa et al. 2020) is key for ensuring the prevention of and adaptation to natural hazards and ensuring the sustainable provision of FES (Keenan 2015; Lecina‐Diaz et al. 2021). A first step would be to assess and correct redundancies and ambiguities of forest-related investments. Then, local to regional forestry and nature conservation administrations should oversee the articulation and implementation of those investments. This should be implemented and monitored in a short–medium term to ensure that each forest-related investment fosters sustainable solutions with regard to multiple forest functions. Every economic support needs to be continuous and outcome-oriented by designing policies that consider spatial targeting to FES density, threats and cost levels, payment differentiation, and improved conditionality (Wunder et al. 2020). This solution requires an integrated forest policy that addresses various system dimensions in terms of policy sectors and administrative levels, including both local and landscape-level land uses with indicators oriented towards minimizing socio-ecological damages and losses (Moreira et al. 2020).

##### Top 4. Support economic instruments and business models promoting regulating and cultural FES with consistent policies

*Sustainability Area: Economy; Challenge 8—Economic power asymmetries in the forestry sector*

Effective economic instruments as well as business models that contribute to the sustainable provision of FES (particularly for regulating and cultural FES) should be consistently supported by cross-scale European and national policies similar to those in place for timber and biomass production (Wunder et al. 2019). This could be achieved through, on the one hand, nested multi-scale policies (Ostrom 1990) and, on the other hand, a strategy of advertising and making available successful business models (along with the key features leading to their success). The specific purpose would be to stimulate their replication elsewhere. In relation to incentive-based and result-based payments for ecosystem services (PES) schemes, it is important to target forest owners of those forest areas that show a) high levels of FES supply (e.g., high carbon stocks/ha or endemic biodiversity hotspots), and b) areas with high potential risks (e.g., high threat of deforestation and degradation). This strategy would focus PES in areas where they can realistically make a difference (Börner et al. 2020; Wunder et al. 2020).

##### Top 5. Engage the community in participatory decision-making in management approaches in public forests, while embracing innovations towards efficient use of forest resources.

*Sustainability Area: Governance; Challenge 12—Tensions and mismatching expectations about the role of public forests*

This solution strategy promotes participatory forest management to overcome outdated management approaches that do not respond to current societal demands and larger social–ecological challenges (such as biodiversity loss or climate change). These strategies are often coupled with a philosophy of embracing innovations towards improved forest management for the provision of FES bundles, especially for regulating and cultural FES, for the promotion of ecological and societal transformation, and for the sustainable use of public goods. Public forests would be used as niches of innovation (Geels 2005) of, for example, public–private partnerships or novel actor alliances to improve the provision of regulating and cultural FES or enhance non-wood forest product (NWFP) value chains. Public forests would act as ‘incubation rooms’ for radical novelties, providing locations for learning, and spaces to build social networks which support innovation. Initiatives and innovations would be carefully addressed so that public resources do not end up creating exclusively private benefits, but rather improving local economies with a share of benefits re-invested in improved forest management.

##### Top 6. Implement practices for (re)connecting people with forests

*Sustainability Area: Socio-culture; Challenge 13—Homogenization of perceptions of forest values by society*

Understanding forests as a mean to solve economic problems is a reductionist standpoint. In the pursuit of sustainable forest management, increased identification and inclusion of cultural bonds is crucial. To achieve a deeper understanding of the mutual constitution of the society–forest relation, it is also necessary to recognize the multi-layered spectrum of forests’ contributions (Ritter and Dauksta 2013). Mainstreaming forest models that (re)connect people and forests (like forest kindergartens and forest schools) is crucial. Increasingly, studies show the perceived linkages of people to spiritual and cultural values in forests that are not necessarily related to livelihoods (Rodríguez-Morales et al. 2020; Torralba et al. 2020). In parallel, there is a need to strengthen the social and cultural sciences in FES assessments with a clearer representation of non-material values (Jacobs et al. 2016) and more-than-human thinking (Whatmore 2006).

##### Top 7. Promote new forms of communication and interaction between society and FES providers with a focus on public goods

*Sustainability Area: Socio-culture; Challenge 14—Conflicts between FES providers and beneficiaries*

When forests provide more regulating or cultural services than provisioning services, governance mechanisms are key to maintaining the supply of FES, especially in privately owned forests. To overcome the lack of markets to deal with public goods and services, social support is needed to finance the expenses that keep the sustainable forest management ongoing. This is especially important in situations where management is key to guarantee the provision flow of these goods and services, but where these are under high threat (e.g., wildfire risk in the Mediterranean region that increases with the lack of active forest management). European studies of public perception (Rametsteiner et al. 2009) have revealed that forestry issues are not well understood outside the forestry community and have suggested that improving communication to the general public is essential. Management goals and objectives must be identified and communicated on the short as well as long term, a wide variety of channels should be used, messages should be simple and clear, and collaboration with other organizations (agriculture, wood construction, etc.) should be enhanced. A joint effort with media professionals would lead to results that are more successful. In parallel, further research into the public perception of forests and forestry is needed to define targeted communication strategies (Fabra-Crespo and Rojas-Briales 2015).

##### Top 8. Improve integration of regulating forest ecosystem services in local and regional planning

*Sustainability Area: Environment; Challenge 1—Increasing frequency and intensity of extreme weather events*

This solution proposes that forest planning authorities consider to a larger extent those specific strategies that have been proven to enhance regulating services such as watershed protection, erosion prevention, or flood control, for example by promoting mixed forest stands of uneven ages (Bravo-Oviedo 2018; Felipe-Lucia et al. 2018). These should be economically supported to cover the opportunity costs needed to restructure forests. Such measures, like PES, already exist in some settings worldwide with different degrees of success (Wunder et al. 2020). The implementation of PES has been polarized between pro-market and anti-neoliberal arguments. A political–cultural reconceptualization should be achieved to attain their potential while ensuring an improved environmental governance, (Van Hecken et al. 2015). Moreover, PES implementation may encounter obstacles hampering the promotion of regulating FES and impeding the improvement of the socio-economic situation of forest-dependent communities and stakeholders. Some of these obstacles are on the social side, the lack of know how, insecure property rights, and problematic benefits distribution, on the market side, the adverse PES self-selection, inadequate administrative targeting, and enforced conditionality (Pagiola et al. 2005; Wunder et al. 2020). There is a large potential for the adaptation of these experiences to the European context.

##### Top 9. Coordinate strategic regional forestry stakeholders to join forces against biological and environmental threats

*Sustainability Area: Environment; Challenge 2—Increasing extent, frequency and impacts of events in forest habitats*

This solution proposes the regional-level implementation of coordinated actions and monitoring strategies. Risk can be assessed using analytical techniques that account for threats both spatially and temporally. Subsequently, risk-management strategies need to account more fully for multi-level responses that act to balance conflicting interests between stakeholder organizations concerned within the managed and natural environments (Mills et al. 2011). These strategies would integrate private and public forest owners together with the regional–national administration and other sectors depending on the context (e.g., nature conservation, local communities), and be backed with national support. The objective would be to share knowledge about affected areas and to join forces for specific forest interventions, increasing the readiness, monitoring capacity, and hence increasing the resilience of the system to these perturbations. An example comes from some regions in the Mediterranean, where civil society engages in wildfires extinction through volunteer groups (Górriz-Mifsud et al. 2019). Coordination strategies would need to be specifically adapted to each context. Transferability can be hampered by the heterogeneous systems of management and governance in Europe.

##### Top 10. Increase availability, volume, and accessibility of financial instruments to cover losses from natural hazards

*Sustainability Area: Economy; Challenge 7—Insufficient financial support to changing conditions*

The current natural hazards require planning and management strategies that increase forests capacity for adaptive transformation. This could provide an opportunity to steer the objectives of forest management towards a more sustainable and less production-oriented model. To be efficient, financial instruments need to be conditional upon sustainable practices that ensure a diverse FES provision, while being adapted to the different realities existing in the European forestry sector. This could be achieved by dedicating part of existing economic support (e.g., EU rural development fund, common agricultural policy, and other regional/local funds) for business model implementation to strengthen their adaptation to each specific context. For example, by refocusing on forest protection measures (Alliance Environment EEIG 2017) and encouraging the use of result-based schemes to increase the impact of the funding, while linking the business model with a positive and measurable impact on the FES provision (ECA 2020). Within such a scheme, a requirement for eligibility to receive funds would be the direct link between the business model and a positive impact FES provision (Wunder et al. 2018; Ovando et al. 2019).

## Discussion—Strategic pathways towards the sustainable supply of FES in Europe

European forests are exposed to fundamental and interconnected threats that put many forest ecosystem services that are vital for human well-being at risk. At the same time, various national and EU-wide policies are rapidly emerging in Europe, which try to solve pressing societal challenges with a forward-looking view on FES potential (Primmer et al. 2021). A diagnosis of FES provision, integrating different perspectives from science, policy, and practice is crucial to understand where the flaws of forest socio-economic systems are so that solutions can be strategically designed and implemented.

### Deep and shallow leverage solutions

Most of the proposed solutions were considered as highly effective, transferable, and susceptible of being monitored over time. while none of them was evaluated as economically inefficient by the team of experts. However, more than half of the proposed solutions were considered not to be yet ready for implementation or currently feasible. This is particularly relevant for those solutions that imply a multi-level governance component and/or coordination among vertical and horizontal levels of actors (e.g., solutions 5 and 21). These types of solutions would normally require long-term commitment, institutional changes, and socio- political will (e.g., solutions 18, 24). Furthermore, they directly or indirectly interfere with long-established cultural elements or strong economic interests (e.g., solution 12). These solutions can be considered as aiming for or being dependent on larger, perhaps even fundamental system changes which require the alteration of existing paradigms, institutions (such as policies but also mindsets), and actors’ behaviors.

A closer look at the solutions’ definitions and prioritization suggests a possible sequence of implementation. Inspired on the notion of leverage points, where solutions can induce shallow or deep changes (Abson et al. 2017; Dorninger et al. 2020), we could arrange the prioritized solutions according to their potential to solve the challenges for the sustainable provision of FES. While there are some low-hanging fruits, which could be easily implemented, some of the proposed solutions require a longer and more sustained effort due to their profound transformative potential and respective resistance. Advances towards the implementation of the former, which could be seen as encompassing fundamentally paradigm change solutions, would smooth the way for the later, which could be seen as managerial solutions. This is best illustrated with the highest ranked solutions. The strategic solution of “mainstreaming FES-oriented management in a threefold strategy: education, awareness, and networking” is focused on changing mindsets towards an integrated multiple FES thinking and has the potential to shift the classic market-oriented economic rationale that reinforces a timber production-oriented paradigm. Similarly, the solution of the “promotion of climate-smart and resilient forest” is fundamental to ensure the adaptation of existing forests to the conditions and disturbance regimes associated to climate change so that they can continue to provide FES. This solution is the precondition for targeting several economic, socio-cultural, and environmental challenges.

Due to the complexity inherent to the forestry sector and the entangled character of the challenges, the proposed solutions are highly interconnected, which pledges for a need for system change across all sectors, levels, and actors. A paradigm shift affecting institutions, academia, and forestry administrations is needed to go beyond forest biomass production and leverage the costs induced by investing into regulating and cultural FES.

### The seven pathways towards sustainable FES supply

**Fig. 3:**
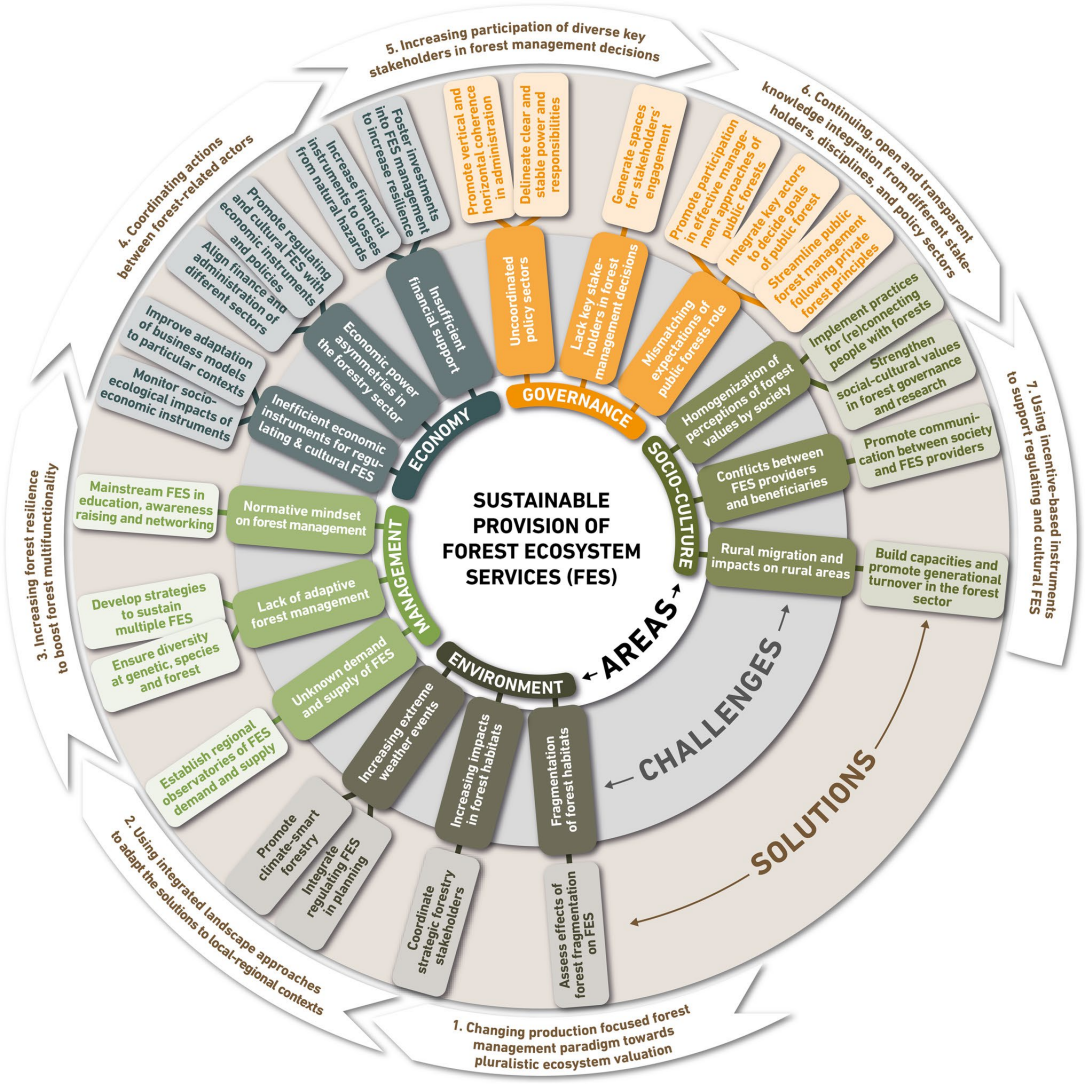
Seven strategic pathways for the sustainable supply of FES in Europe

Many of the solutions have synergistic effects if they are combined and implemented in an orchestrated manner according to their capacity to enable transformation. For example, the integration of social–ecological values proposed in solution 21, could support and benefit from the regional observatories proposed in solution 8. By looking into the elements that are at the core of each individual solution, we propose seven emerging pathways on which European forest policies should focus in the mid- and long-term to ensure the sustainable supply of multiple FES. These strategic pathways could collectively build the backbone of European forest policy implementation. Although all of them are relevant, they can be distinguished by their capacity to leverage change in European forests and to secure the supply of multiple FES in relation to future disturbances and social–ecological changes (Fig. 3). Collectively, the seven identified strategic pathways can be organized in a hierarchical order, where deep forestry system transformation would be in the basis, followed in the middle and top by system-based management strategies and concrete measures.

#### Changing production focused forest management paradigm towards pluralistic ecosystem valuation (Core element of solutions 6, 11, 22, 23, and 24; relevant for challenges 5, 8, 13, 14 and 15)

Decision-making processes affecting FES provision need to embrace broader views, preferences, and values from a multi-actor perspective. Expanding the focus towards regulating, cultural, and supporting FES and understanding their valuation from a pluralistic and integrative point of view would advance the (re)connection between people and nature. Forestry education at all levels, forest management, and policies need to pursue a shift from the consideration of instrumental values, to increasingly considering intrinsic and especially relational value dimensions (Chan et al. 2016; Jacobs et al. 2016).

#### Using integrated landscape approaches to adapt the solutions to local–regional contexts (Core element of solutions 1, 2, 5, 7 and 10; relevant for challenges 1, 4, 5 and 7)

Due to the inherent heterogeneity of European forests, future policies need to embrace the context-specificity of forest social–ecological dynamics, and use the landscape scale as the most appropriate one to address the multi-scalar pressures on forests (Opdam et al. 2018). A landscape scale provides the framework to orchestrate problems related to improving coordination and transparency in decision-making processes (Termorshuizen and Opdam 2009; Sayer et al. 2013).

#### Increasing forest resilience to boost forest multi-functionality (Core element of solutions 3, 8, 13, 14 and 23; relevant for challenges 2, 6, 9 and 14)

Integrated landscape solutions promoting forest resilience and multi-functionality should, therefore, be at the forefront of forest policies. It is fundamental to increase European forest resilience by lessening intensive management to ensure adaptation to fluctuating climatic conditions. As recently observed by Pohjanmise et al. (2021) in boreal forests, - is substantially diminished under intensive forestry and recovers slower, the longer intensive forestry has been implemented.

#### Coordinating actions between forest-related actors (Core element of solutions 4, 5, 12, 15 and 16; relevant for challenges 3, 4, 8 and 10)

The lack of coordination across forestry stakeholders and among different administrative levels can currently be considered an entrenched problem in the European forestry context (Winkel and Sotirov 2016). However, its disentanglement is a requirement for the successful implementation of any forest policy. Once a multifunctional view on forests is emerging and impregnated through educational programs and policies, the forest institutional and social fabric would be better disposed to implement coordinated actions.

#### Increasing participation from a larger diversity of stakeholders during forest planning and management, with a focus on public forests (Core element of solutions 5, 7, 17, 18, 20 and 22; relevant for challenges 4, 5, 11, 12 and 13)

Greater levels of participation from the public into forest management decision-making is at the essence of several solutions. To do so, forest policies should increasingly promote participation in coordinated multi-level governance models (Muradian and Rival 2013) by using for example collaborative digital tools, and capitalize from ongoing and former initiatives engaged in the provision of FES and nature models that have proved successful in ecosystem management and conservation (Armitage et al. 2020).

#### Continuing, open and transparent knowledge integration from different stakeholders, disciplines, and policy sectors (Core element of solutions 5, 6, 9 and 21; relevant for challenges 4, 5, 8 and 13)

Translating sustainable management policy objectives into action on the ground has been described as a “wicked problem” (Duckett et al. 2016). This leverage area is fundamental to establish a fluid dialog to value and to integrate perspectives from “outsiders” disciplines and sectors affecting forests. To do so, several solutions point towards the use of inter- and transdisciplinary approaches as a way to integrate available knowledge and to create ownership for problems and solution options (Lang et al. 2012).

#### Using incentive-based instruments to support regulating and cultural FES (Core element of solutions 2, 8, 11, 13 and 14; relevant for challenges 1, 6, 8 and 9)

PES and PES-like schemes are currently scarce in Europe. An increased role for PES could manifest itself through government-financed PES (e.g., through flexible reforms of the Common Agricultural Policy), or through user-financed PES in those areas where there is sufficient willingness to pay for a specific FES (Wunder et al. 2020).

## Conclusions

EU policy frameworks such as the New Green Deal and the Forestry Strategy offer a unique opportunity to serve as catalysts for solving the challenges hindering the sustainable supply of FES. To support this endeavor, the scanning exercise presented here not only disentangles the most pressing challenges in all sustainability areas but also offers a set of prioritized solutions to each of those challenges. Just as the assessed hindrances affect each other, similarly the strategic solutions can be used synergistically. This way, like concentric levels of mutually supportive implementation (Fig. 3), a paradigm shift to better integrate pluralistic values of forests in a more balanced way would sustain the rest of the strategic solutions. Next, increasing forest resilience through integrated landscape approaches should be prioritized followed by strategies promoting coordinated, inclusive, and transparent decision processes. The next strategies would focus on enabling forest's biophysical conditions to support the balanced supply of FES, while reinforcing the social fabric of forest governance to make it more cohesive. Finally, incentive-based mechanisms could, depending on the local context, promote a management that strengthens the sustainable supply of multiple FES.

## Supplementary Information

Below is the link to the electronic supplementary material.

Supplementary file1 (DOCX 4419 KB)

Supplementary file2 (XLSX 15 KB)
