# Supplementary material for: Scanning the solutions for the sustainable supply of forest ecosystem services in Europe
Source: Sustain Sci. 2022 Mar 22;17(5):2013–29. doi: 10.1007/s11625-022-01111-4 (PMC8939503; doi:10.1007/s11625-022-01111-4)
Supplement: Supplementary file 1 — Supplementary file1 (DOCX 4419 KB) [file 11625_2022_1111_MOESM1_ESM.docx]

**S1. Exploratory Survey**

**Solution Scanning: Sustainable Provision of Forest Ecosystem Services (FES) in Europe**

--------------------------------------------------------

**Personal Information:**

Name

Organization

-------------------------------------------------------------------------------------------------

**Identification of Challenges**

1. In your opinion, what are the most pressing ENVIRONMENTAL CHALLENGES affecting the sustainable provision of multiple FES? (Please, provide at least two challenges and elaborate your answer)
2. In your opinion, what are the most pressing SOCIETAL CHALLENGES affecting the sustainable provision of multiple FES? (Please, provide at least two challenges and elaborate your answer)
3. In your opinion, what are the most pressing ECONOMIC CHALLENGES affecting the sustainable provision of multiple FES? (Please, provide at least two challenges and elaborate your answer)
4. In your opinion, what are the most pressing MANAGEMENT CHALLENGES (incl.technology) affecting the sustainable provision of multiple FES? (Please, provide at least two challenges and elaborate your answer)
5. In your opinion, what are the most pressing GOVERNANCE CHALLENGES affecting the sustainable provision of multiple FES? (Please, provide at least two challenges and elaborate your answer)
6. Is there any other challenge not addressed in the above categories?
7. In your opinion, what are the most important research gaps to promote the multiple FES in EU forest?

----------------------------------------------------------------------------------------------------------------

**S2. Solution Assessment Survey**

**Solution Scanning: Sustainable Provision of Forest Ecosystem Services (FES) in Europe**

-----------------------------------------------------

**Personal Information:**

Name

Organization

Please prioritize each solutions using the Likert scale (from 1=very little to 5=very much) according to the following criteria:

1. SOCIAL-ECOLOGICAL EFFECTIVENESS (SEE): the degree to which, the solution respects the natural and social environment and/or improves it.

2. ECONOMIC EFFICIENCY (EE): the degree to which the resources needed for implementing the solution are allocated to their most valuable uses and waste is eliminated.

3. READINESS (R): the degree to which the solution can be implemented in the shortest period of time.

4. ASCERTAINMENT AND MONITORING (AM): the degree to which the solution can be tracked and assessed.

5. FEASIBILITY (F): the degree to which the solution can be successfully accomplished.

6. TRANSFERABILITY POTENTIAL (TP): the degree to which the solution can be transferred to other contexts.

|  |  |  | Socio-Ecological  Effectiveness | Economic  efficiency | Ascertain. Monitoring | Readiness | Feasibility | Transfer.  potential |
| --- | --- | --- | --- | --- | --- | --- | --- | --- |
| Area | Challenge | Solution |  |  |  |  |  |  |
| Environment | Challenge 1. Increasing frequency and intensity of extreme weather events | Solution 1 - Promote climate-smart forestry and forest resilience |  |  |  |  |  |  |
|  |  | Solution 2 - Improve integration of regulating forest ecosystem services in local and regional planning |  |  |  |  |  |  |
|  | Challenge 2. Increasing extent, frequency, and impacts of events in forest habitats | Solution 3 - Coordinate strategic regional forestry stakeholders to join forces against biological and environmental threats |  |  |  |  |  |  |
|  | Challenge 3: Fragmentation of forest habitats | Solution 4 - Implementation of systematic and comprehensive environmental assessments considering multiple scales and cumulative effects of forest fragmentation on FES at landscape level |  |  |  |  |  |  |
| Management | Challenge 4. Narrow focus and normative mindset on forest management | Solution 5 - Mainstreaming FES-oriented management in a threefold strategy: education, awareness raising, and networking |  |  |  |  |  |  |
|  | Challenge 5. Lack of adaptive forest management practices | Solution 6 - Develop adaptive strategies to sustain multiple FES based on regional scenarios |  |  |  |  |  |  |
|  |  | Solution 7 - Ensure diversity at different levels (genetic, species, and forest) |  |  |  |  |  |  |
|  | Challenge 6. Unknown demand and supply of FES | Solution 8 - Establish regional observatories for capturing societal FES demand and supply |  |  |  |  |  |  |
| Economy | Challenge 7. Insufficient financial support to changing conditions | Solution 9 - Foster investments into FES oriented forest management to increase resilience (prevention and adaptation) towards natural hazards |  |  |  |  |  |  |
|  |  | Solution 10 - Increase availability, volume, and accessibility of financial instruments to cover losses from natural hazards |  |  |  |  |  |  |
|  | Challenge 8. Economic power asymmetries in the forestry sector | Solution 11 - Support economic instruments and business models promoting regulating and cultural FES with consistent policies |  |  |  |  | 3,55 |  |
|  |  | Solution 12 - Align finance and administration of different sectors |  |  |  | 2,36 |  |  |
|  | Challenge 9. Lack of efficient economic instruments and business models for regulating and cultural FES | Solution 13 – Improve adaptation of business models to particular contexts of implementation |  |  |  |  |  |  |
|  |  | Solution 14 - Monitor systematically the socio-ecological impact of economic instruments |  |  |  |  |  |  |
| Governance | Challenge 10. Lack of coordination and competition among different policy sectors | Solution.15 - Promote vertical and horizontal coherence in administration |  |  |  | 2,36 |  |  |
|  |  | Solution 16 - Delineate clear and stable power and responsibilities |  |  |  |  |  |  |
|  | Challenge 11. Lack of representation of diverse key stakeholders in forest management decision | Solution 17 - Generate spaces for stakeholders’ engagement and representation in decision making processes in cooperative and participative approaches |  |  |  |  |  |  |
|  | Challenge 12. Tensions and mismatching expectations about the role of public forests | Solution 18 - Engage the community in participatory decision-making in management approaches in public forests, while embracing innovations towards efficient use of forest resources |  |  |  |  |  |  |
|  |  | Solution 19 - Integrate all actors in participatory decision making about management goals of public forest lands |  |  |  |  |  |  |
|  |  | Solution 20 - Streamline public forest management organization and administration following the principles of the private forest sector |  |  |  |  |  |  |
| Socio-culture | Challenge 13. Homogenization of perceptions of forest values by society | Solution 21 - Implement practices for (re)connecting people with forests |  |  |  |  |  |  |
|  |  | Solution 22 - Strengthen the recognition, identification, and integration of social-cultural values in forest management, governance, and research |  |  |  |  |  |  |
|  | Challenge 14. Conflicts between FES providers and beneficiaries | Solution 23 - Promote new forms of communication and interaction between society and FES providers with a focus on public forests |  |  |  |  |  |  |
|  | Challenge 15. Rural migration and impacts on rural areas | Solution 24 - Build capacities as a tool to prevent abandonment and promote generational turnover in the forest sector |  |  |  |  |  |  |

**S6. Solution scanning workshop: Sustainable Provision of Forest Ecosystem Services in Europe**

# [Contents 1](#_Toc94275451)

[Day 1: Exploring the Challenges 1](#_Toc94275452)

[Part I: Evaluation, prioritization and selection of the challenges 2](#_Toc94275453)

[Group 1 - ENVIRONMENT 2](#_Toc94275454)

[Group 2 – SOCIO-CULTURAL 6](#_Toc94275455)

[Group 3 – ECONOMIC 9](#_Toc94275456)

[Group 4 – MANAGEMENT 10](#_Toc94275457)

[Group 5 – GOVERNANCE 12](#_Toc94275458)

[Part II: Characterization of the challenges 14](#_Toc94275459)

[GROUP 1 – ENVIRONMENT 14](#_Toc94275460)

[Group 2 – SOCIO-CULTURAL 20](#_Toc94275461)

[Group 3 – ECONOMIC 23](#_Toc94275462)

[Group 4 – MANAGEMENT 25](#_Toc94275463)

[Group 5 – GOVERNANCE 28](#_Toc94275464)

[Part III: Plenary and selection of top 3 challenges per area 30](#_Toc94275465)

[Day 2: Identifying the Strategic Solutions 34](#_Toc94275466)

[Part IV-V: Identification and characterization of the strategic solutions 34](#_Toc94275467)

[Group 1 - ENVIRONMENT 34](#_Toc94275468)

[Group 2 – SOCIO-CULTURAL 37](#_Toc94275469)

[Group 3 – ECONOMIC 41](#_Toc94275470)

[Group 4 – MANAGEMENT 47](#_Toc94275471)

[Group 5 – GOVERNANCE 49](#_Toc94275472)

[Part VI – Plenary 52](#_Toc94275473)

# **Day 1: Exploring the Challenges**

## Part I: Evaluation, prioritization and selection of the challenges

### Group 1 - ENVIRONMENT


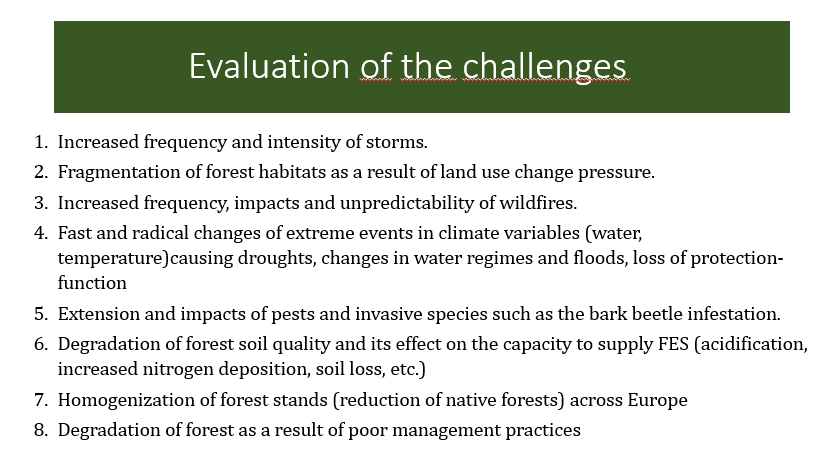


Some of the challenges were changed from their original formulation:

| **Received Challenge Wording** | **Discussion** | **Changed to** |
| --- | --- | --- |
| 1. Increased frequency and intensity of extreme weather events (e.g. storms and flooding). | A strong overlap with challenge 4 was discussed. To prevent treating the topic of precipitation and temperatures twice we changed the wording here to only include storms. We were unsure what is meant by increase of flooding in forests. Are forests affected by floods or is the protective function against floods meant? We concluded that most likely the later option is meant and thus included the protective function in challenge 4.  At a later stage we discussed whether to include wildfires here (we decided against it as solutions might differ. We discussed briefly that avalanches are not included with the new wording. | 1. Increased frequency and intensity of storms. |
| 2. Degradation of forest habitats and biodiversity loss. | We identified a strong overlap / correspondence with challenge 8. Moreover, we discussed that this challenge is rather a consequence of other challenges and thus reformulated it to a challenge that does not emphasize the consequence but its origin. At a later stage we removed the word “pressure” as land use change can also be seen as land abonnement which might result in a fragmentation regarding specific habitat conditions certain species might need. Later, we added in additional challenge about the degradation of forests as the solutions differ between degradation and fragmentation, thus in total we just kind of exchanged original challenge 2 and 8. | 2. Fragmentation of forest habitats as a result of land use change pressure. |
| 3. Increased frequency, impacts and unpredictability of wildfires. | We saw this challenge as being related to challenge 5 (increased biomass). We concluded that challenge 5 is the solution for challenge 3. | 3. Increased frequency, impacts and unpredictability of wildfires. |
| 4. Fast and radical changes of rain-drought regimes. | We emphasised water and temperature here, to clearly separate this challenge from the first one. During a later stage of the process, the words in brackets were deleted. | 4. Fast and radical changes of extreme events in climate variables (water, temperature) causing droughts, changes in water regimes and floods, loss of protective-function |
| 5. Increased biomass (largely unmanaged) in European forests. | Comments from the chat during plenary: L.T. why increased biomass is a challenge? Does it link to increased risk to wildfires? Otherwise it may link to increased biodiversity?, M.: Yes it is in the context of maintenance of forest functions, Competition for water and risk of wildfires . We see this as being the cause for challenge 3 and thus merged these challenges (content-wise, not in the wording). |  |
| 6. Extension and impacts of pests and invasive species such as the bark beetle infestation. | Agreed. At a later stage reformulated to Increasing frequency and impacts of…. Bark beetles were put in brackets to not forget about other diseases and insects for which solutions might strongly differ. | 5. Extension and impacts of pests and invasive species such as the bark beetle infestation. |
| 7. Degradation of forest soil quality and its effect on the capacity to supply FES (acidification, increased nitrogen deposition, soil loss, etc.) | Related to type of management and content. Perhaps not enough expertise in this group about this challenge. | 6. Degradation of forest soil quality and its effect on the capacity to supply FES (acidification, increased nitrogen deposition, soil loss, etc.) |
| 8. Land use change pressure (e.g. agriculture and urbanization) and (or leading to)  fragmentation of forests | We discussed whether to separate these into two challenges and identified challenge 2 as a result of this challenge. Thus the eights challenge was integrated with the second challenge | - |
| 9. Homogenization of forest stands across European forests. | We discussed that this challenge is related to challenge 5 and that it can also be the result from a reduction of native forests. Later, we decided that reduction of native forests is very area specific, we have little knowledge about it and thus deleted the words in brackets. | 7. Homogenization of forest stands (reduction of native forests) across Europe |
|  | Management originating from central europe is brought to mediteraenean regions without adaptation. Thus we added this challenge which closly resembles original challenge 2 (which we did not notice during this process). | 8. Degradation of forest as a result of poor management practices |

Prioritization of the challenges:


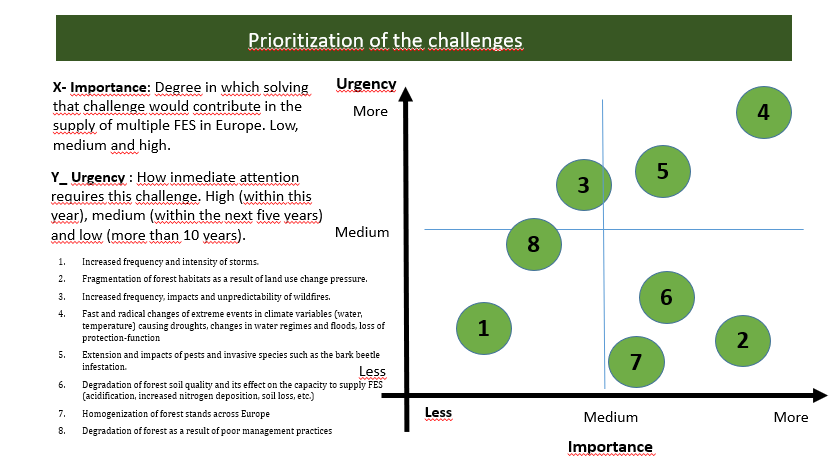


During the priotization, the main questions used for guiding the discussion were:

- Is the importance reduced when the challenge is only relevant for parts of Europe?
- Urgency: Is the theoretical starting point or realistic time-frame meant? We decided on the definition: “need to start doing something soon”
- No challenge is not important, just “less” -> the wordings at the coordinate system were changed to less/more. More important are those challenges that can lead to a tipping point of forest (mega-fires)

Final selection of 5 challenges:


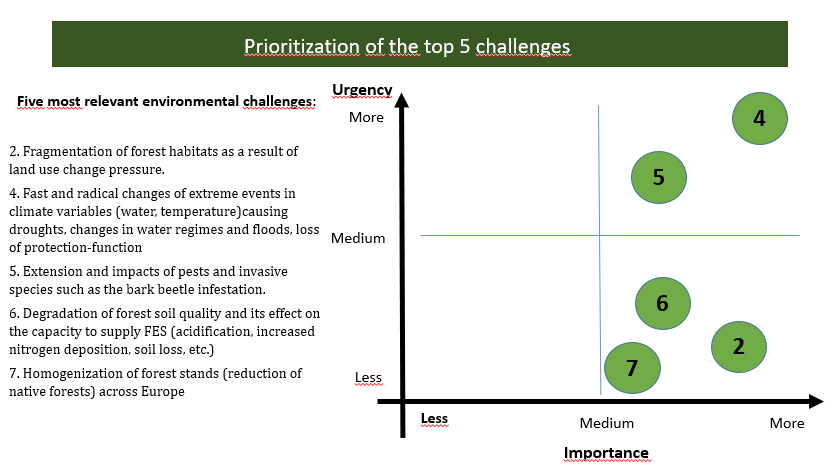


General comment: The wording “five most relevant” is contradictory to prioritization as later implies a ranking.

It was rather a “which one to discard” than a “which one to select” discussion.

Challenge 1 and 8 were discarded because of their location in the coordinate system. We discussed between challenge 3 and 7 and decided against challenge 3 (caused by other challenges, only relevant in some regions, can be related to extreme events)

### *Group 2 – SOCIO-CULTURAL*


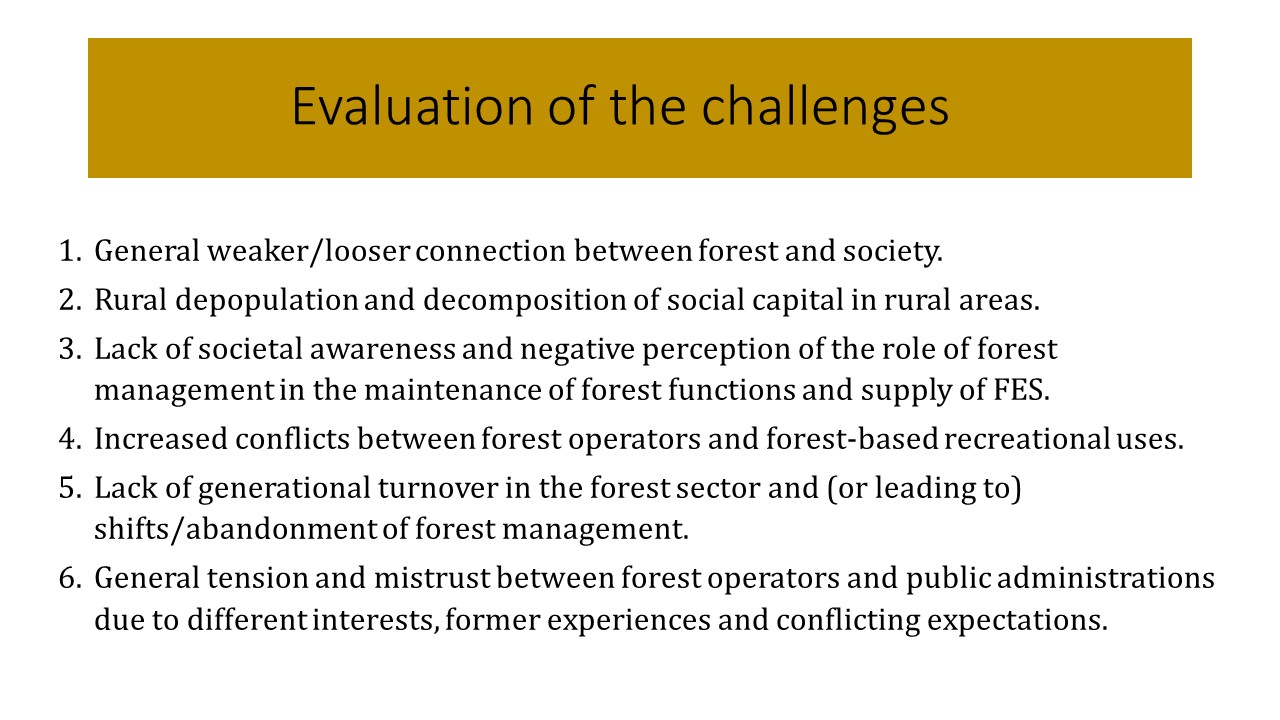


First of all, we briefly discussed what it meant by “social”, and the group agreed that it is everything related to forest and society + cultural dimensions. The question was raised if the conflicts are not more related to governance and so the governance group should tackle them.

We also discussed briefly what is a challenge, and said that it can both be understood as a problem or an opportunity.

Then, we discussed each challenge:

1. **General weaker/looser connection between forest and society.**

An intensified connection can also lead to problems. In Sweden: forest owners are fewer and less connected to the forest and operations. Immigration and urbanization play an important role in weakening the connection (visits only on weekend, no daily interaction with forest landscapes). We notice that there are highly differentiated perceptions of forest as common good.

We considered modifying the phrasing of the challenge with “changing connection between forest and society”: direct connections are decreasing maybe, sense of place is decreasing // immigration and urbanization change the way forests are perceived.

We discussed diverse antagonisms: Belief that to provide FES we need forest management (society is demanding the FES) >< Society doesn’t like forest management. Demands are fluctuating >< dynamics of the forests are slow.

1. **Rural depopulation and decomposition of social capital in rural areas.**

Not always perceived as a problem, for example it can be positive for biodiversity. It depends on the basic assumptions/perspective. The loss of networks/collaboration however has important implications for innovations.

Some participants to the group thought at this stage that this challenge seemed to be very specific, and not highly relevant overall.

1. **Lack of societal awareness and negative perception of the role of forest management in the maintenance of forest functions and supply of FES.**

What proper management means to different people is diverse, e.g. what makes sense ecologically might be different from economically.

The participants in the group did not really agree with the phrasing of the challenge: “Lack of awareness”, it was said that it sounds like the fault is on society. We should rather talk about a lack of shared vision. Foresters also sometimes do not have the “right” answer. We listed several lacks or gaps: lack of societal awareness of forest as common good for human welfare >< private properties; gap between urban and rural perception of forests, differentiate traditional knowledge and innovative approaches.

The challenge was rephrased as “Lack of knowledge”, and then “fragmented knowledge.

1. **Increased conflicts between forest operators and forest-based recreational uses (uses and users).**

The challenge is about power asymmetries and conflicts. Conflicts often appears when there is limited access on forest private properties, or limited public access rights on private properties. The group agreed that the “conflicts” in question here are not between actors but uses/interests.

1. **Lack of generational turnover in the forest sector and (or leading to) shifts/abandonment of forest management.**

Linked to 2.

1. **General tension and mistrust between forest operators and public administrations due to different interests, former experiences and conflicting expectations.**

Linked to governance, the group decided to delete this challenge from the social list.

Another challenge was added:

**Unclear relationship between property rights and FES as common goods**

As a result, the final list of challenges was:


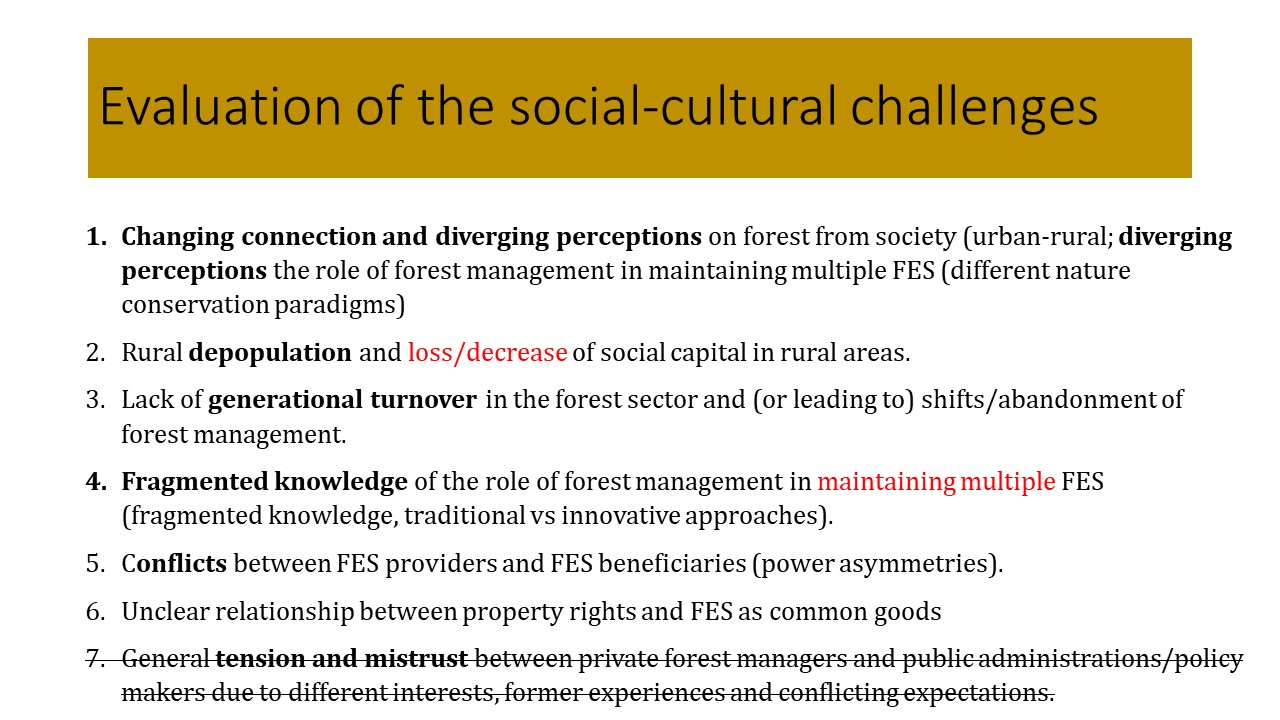


For the prioritization, the order of the challenges was modified by the group (order of importance), and the challenges previously numbered 5 and 6 were merged. We had little time to do this exercise, but the group was unanimous to say that:

- There are all long term challenges, that will take years or decades to change.
- These challenges are related to governance and management.
- Social challenges are about deologies, cultural values, motivations.


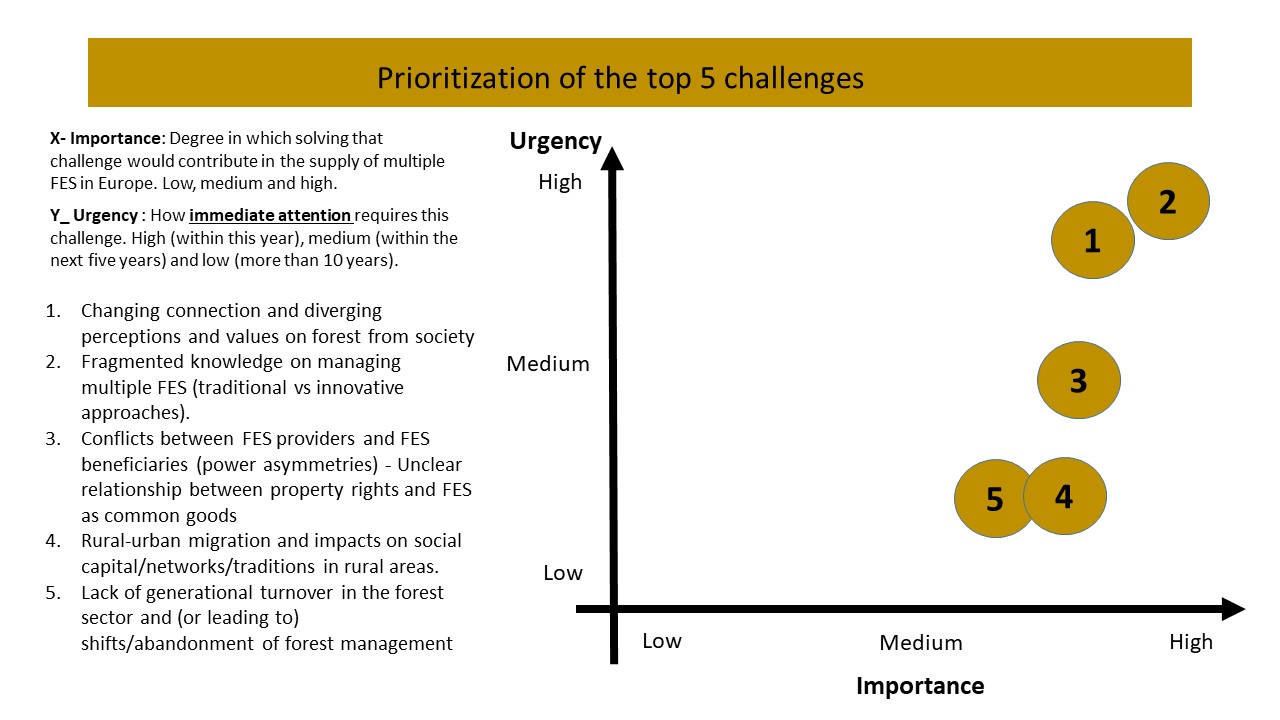


### *Group 3 – ECONOMIC*


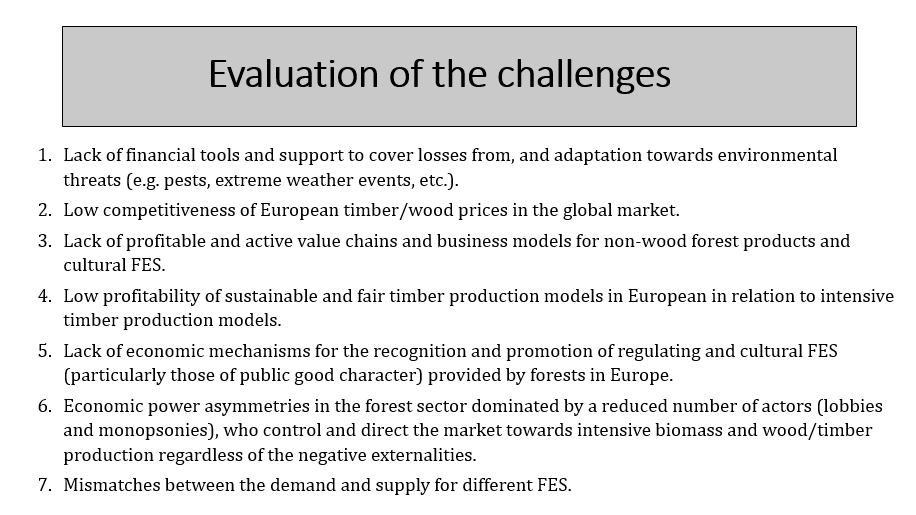


After a series of discussions, the 7 initial challenges were re-formulated in and some cases merged. In particular, in relation to the ross-cutting topics along all challenges such as power asymmetries, demand and supply of ecosystem services and mainstreaming of ecosystem service approach to reach common understanding and possible, targeting two challenges with one solution/approach. The final list of challenges was:

1. **Lack/inefficiency of financial tools** and support to **cover losses** from, and adaptation towards natural hazards (e.g. pests, extreme weather events, etc.).
2. **Lack of economic instruments (incentive based policies, PES) and business models** for the recognition and promotion of **regulating and cultural** FES as well as non-wood-forest-products (particularly those of public good character) provided by forests in Europe (depending on legal frame conditions 🡪 governance, societal awareness 🡪 societal).
3. **Low profitability of multifunctional and close to nature forestry** models (SFM) in Europe in relation to intensive timber production models.
4. **Economic power asymmetries** in the forest sector dominated and influenced by a reduced number of actors (forestry regime, wood processing industry, other powerful sectors such as bioeconomy/bioenergy/biofuel), who control and direct the markets towards intensive biomass and wood/timber production regardless of the negative externalities (depending on legal frame conditions 🡪 governance).
5. **Lack of information/knowledge** on societal demand for different FES (+
   valuation/WTP/trade-offs)


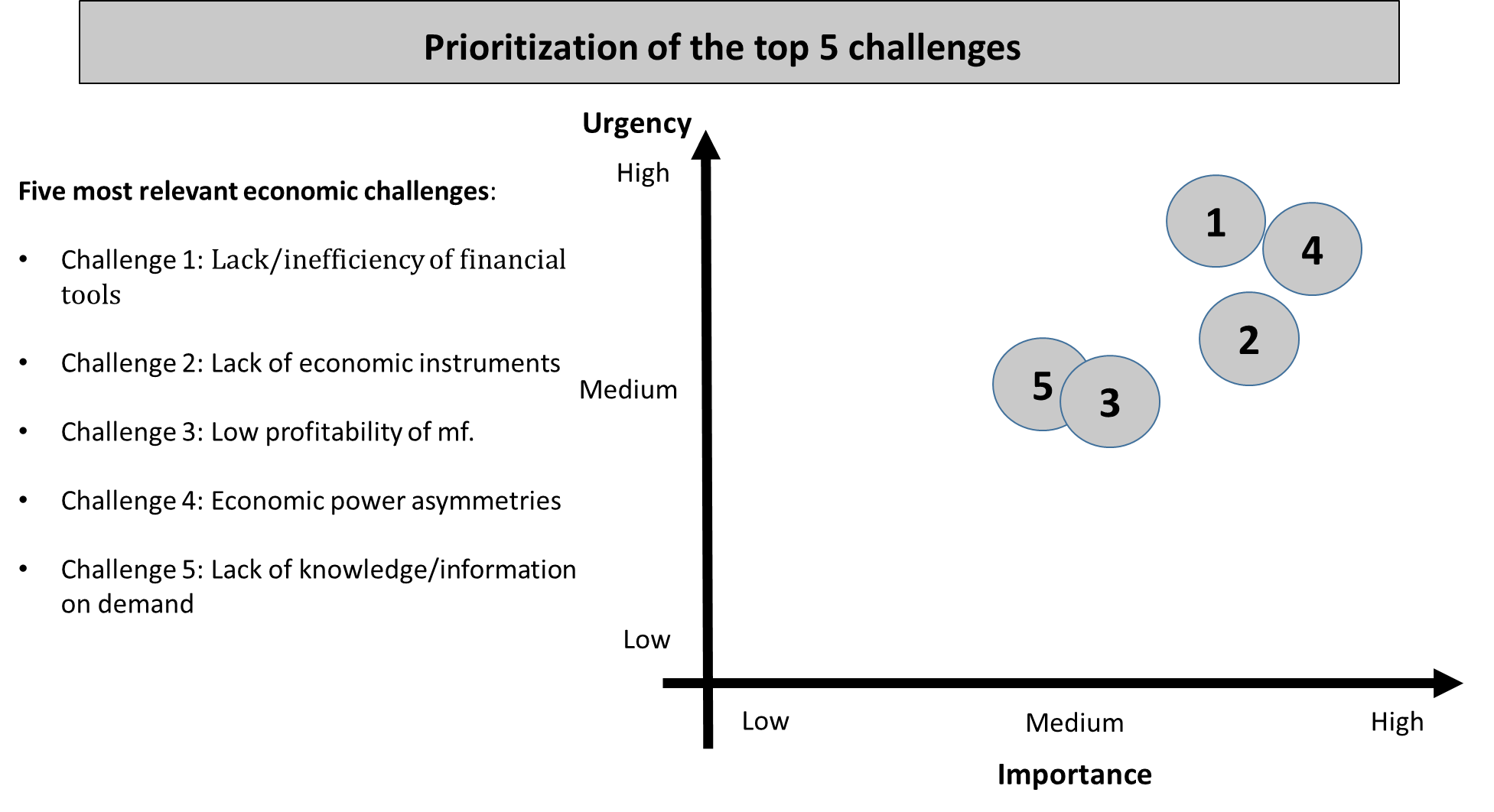


### *Group 4 – MANAGEMENT*


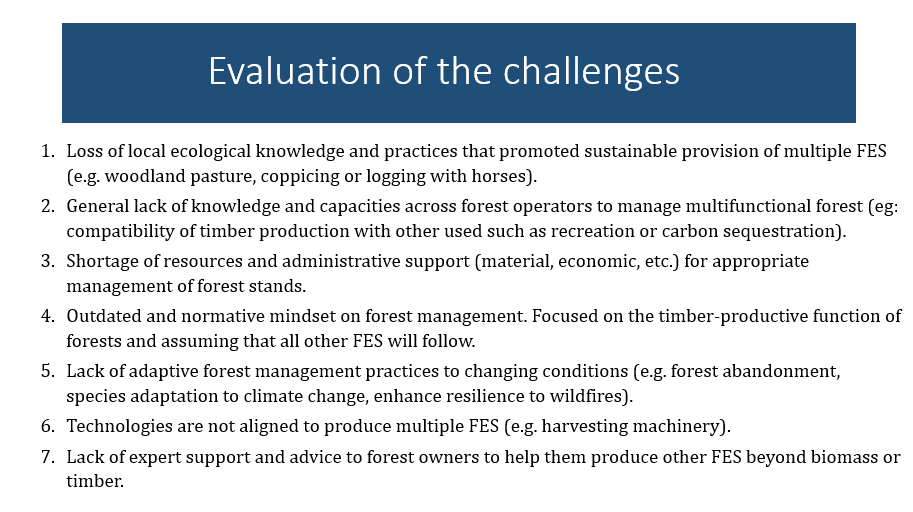


The participants of the Management Group debate vividly each of the Challenges from the North, Central and South European perspectives. It was evident the great differences between South-North priorities, policy goals and capital available.

1. **Loss of local ecological and traditional knowledge and practices that promoted sustainable provision of multiple FES** (e.g. woodland pasture, coppicing or logging with horses). In the south (Spain, Italy), we are facing the loss of TEK and LEK and it would be important and urgent to address. In the north (Finland, Switzerland) it is more about the goal of management oriented to economic purposes and timber production. Three dimensional knowledge there is a loss of local knowledge on the ground.
2. **Shortage of resources and administrative support (material, economic, etc.) for appropriate management of forest stands.** From the north we have the resources, but the value from forest owners have changed, they do not want to focus on timber production any more. We have local associations advising forest owners to enhance the timber production of forest. There are resources to advice but the policy wants to move it to production. It is not only on the goal but on the support of multifunctionality. The political goal is the raw material oriented. From Spain, the owners of forest communities due to a lack of resources and not prioritization of regional administration, as a result it is low in the policy agenda. The shortage is not that importance but the low price of timber is leading the find of new resources more lucrative. What is intended by administrative support.
3. **Outdated and normative mindset on forest management** from academia, administration and forest managers. Focused on the timber-productive function of forests and assuming that all other FES will follow. From the north it is important and urgent. The economic drivers are strong. From North Italy, there is not a big issue to introduced new laws more naturally oriented to provide multiple FES. Generally there is an emerging look at forest as a multifunctional. Andreas, it is important but there are several policies and people with different mindsets… but as an overall it is important. Emilio´s bundles production of FES is not the mainstream in the policy so it is important.
4. **Lack of adaptive forest management practices to changing conditions** (e.g. forest abandonment, species adaptation to climate change, enhance resilience to wildfires). From the north we have management practices that are in the line of adapted species to the new conditions but they are facing the challenges from climate change but they have a nice tool set. It is related to forest owner’s decisions, and in Spain it affects the coping to fight against forest fires. This challenge is affecting other challenges and needs to be tackle. Andreas, there are many programs from climate change that still needs to be coordinated an implemented.
5. **Technologies are not aligned to produce multiple FES** (e.g. harvesting machinery). Francesca, it is not so problematic. People are trained and get the license to raise awareness on the multifunctional FES. In Finland we use a lot of digital information (LIDAR…) but it is difficult to use in selecting cutting but little information is on CES. If you do not know the value of an stand you are managing you cannot protect it properly. The technologies will follow when we have the conservation goals and clarity on the management in place.
6. **Unknown demand and supply of FES for adjust the management strategies accordingly.** From the north we have data on timber values and biodiversity but not much on the demand side from society. When the policy changes you need to produce the data. We would like to monitor the demand of other services. Andreas it is very important to know the demand and supply for forest owners. Regulation and cultural we are less aware of them and it is easier for the provision service. People may not be aware of the importance of some CES. Society take for granted most of FES. An economical evaluation would be important to visualize the value of regulating and cultural services. It is not easy at all.
7. **General lack of expert support to forest owners, forest operators (other stakeholders)** to manage multifunctional forest: Francesca, there is the perception that administration is only focus on production, and that forest owners are seeing forest administration as a threat. Liisa, not much understanding on producing multiple FES, you cannot produce all at the same time. There is a need to balance and combine together the goals. The lack of know how. The multi-stakeholder character, there is a need of a mutual learning, we need management procedure to integrate in the management system.

Based on these discussions and prioritization, we made the selection of the five most relevant challenges:

- Challenge 1: Unknown demand and supply of FES for adjust the management strategies accordingly
- Challenge 2: Outdated and normative mindset on forest management
- Challenge 3: Lack of adaptive forest management practices to changing conditions
- Challenge 4: Loss of local ecological and traditional knowledge
- Challenge 5: General lack of expert support to forest owners, forest operators (other stakeholders)…


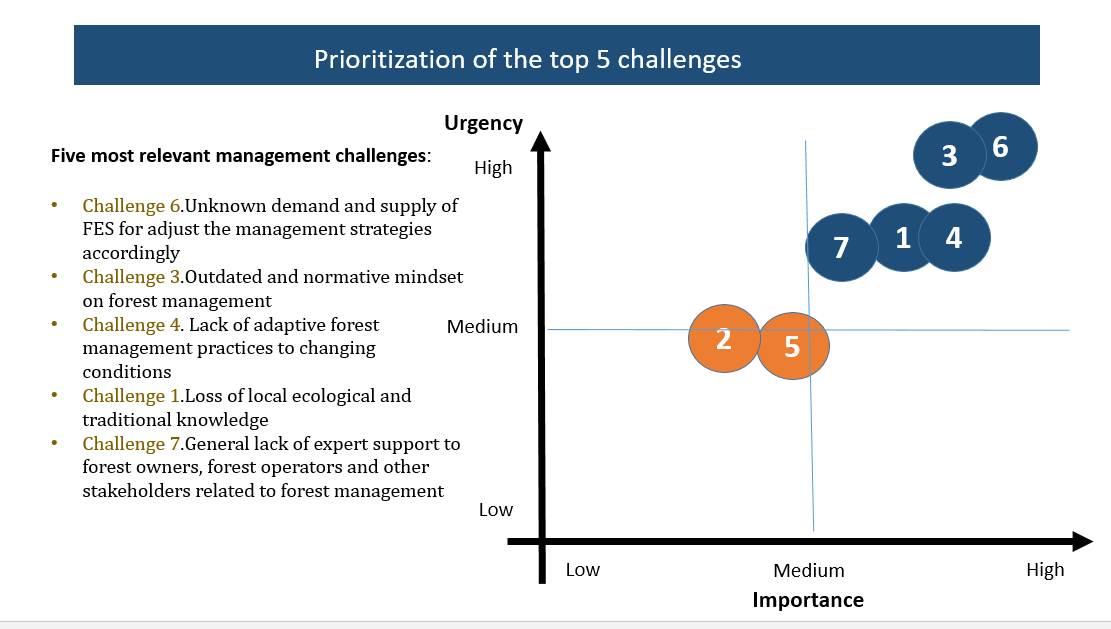


### *Group 5 – GOVERNANCE*


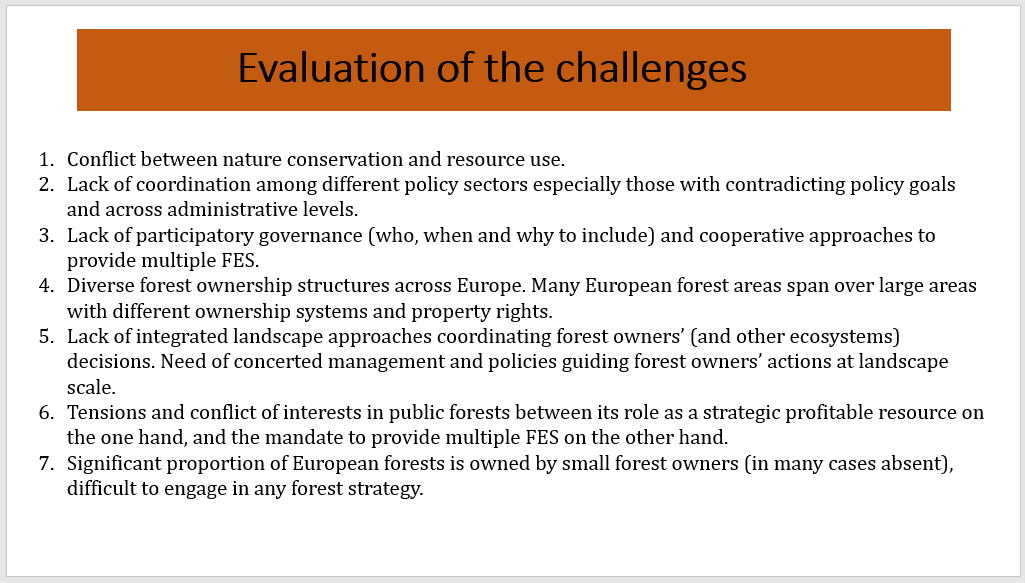


Most of the challenges required some slight re-formulations to make the challenges more concrete and specific. The discussion and explanation of each of the challenges sparked the discussions for that led to the prioritization of the challenges.

After the first of the sessions, the challenges were reformulated as the following:


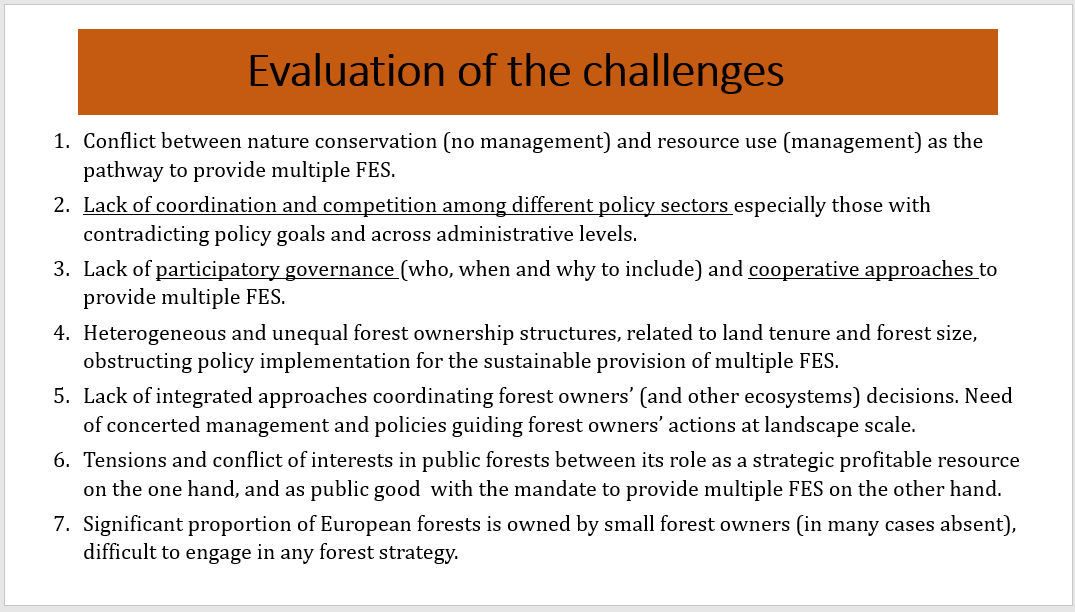


Prioritization and selection of the 5 most relevant challenges: During the discussions, it was considered that challenge 7 would be to a large extent contained within challenge 4, while challenge 5 was fully covered by challenge 2 and 3. By addressing these challenges, there would be no need to assess challenges 5 and 7.

As for the prioritization and selection of the remaining 5 challenges:


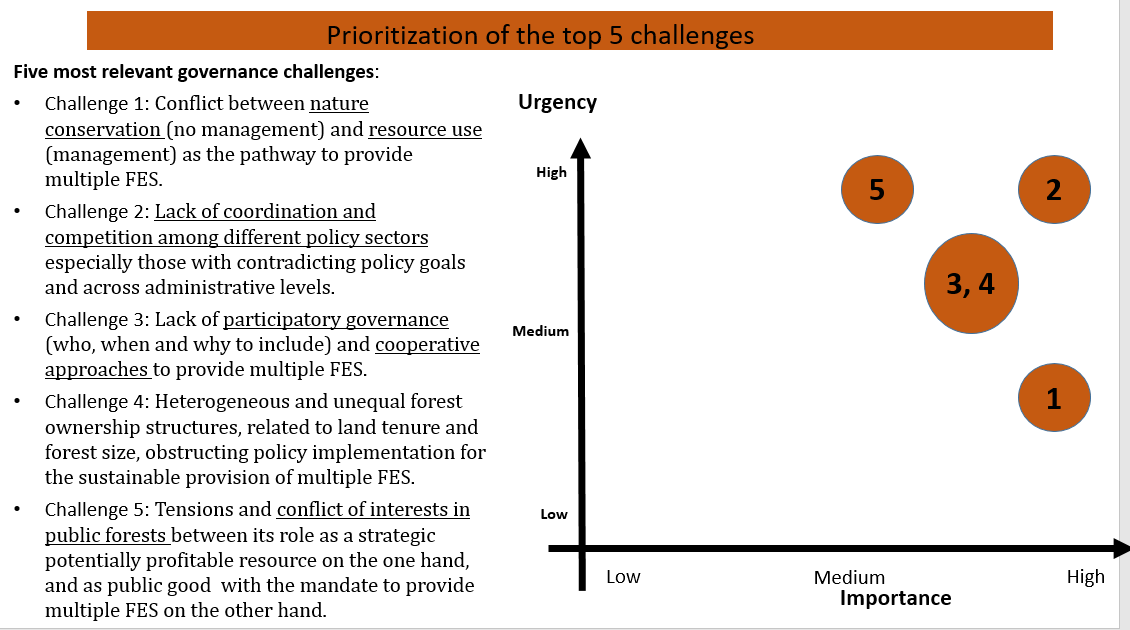


The most pressing challenge would be the lack of coordination and competition among different policy sectors. Challenge 5 (tensions and conflicts in public forest) was considered rather urgent. However, it is a very context-related challenge, as in many European countries there is not much forest that is public. Therefore, it was given a medium relative importance. Challenge 1 (related to the conflict between management and no management to foster biodiversity) is a long-standing challenge. There has been an intense debate over this issue over decades, if not centuries. Therefore it was not considered an urgent matter, although of high relevance. Challenge 3 and 4 were given similar high relevance and urgency.

## **Part II: Characterization of the challenges**

### *GROUP 1 – ENVIRONMENT*

- Challenge 1: Fragmentation of forest habitats as a result of land use change and habitat quality decline.


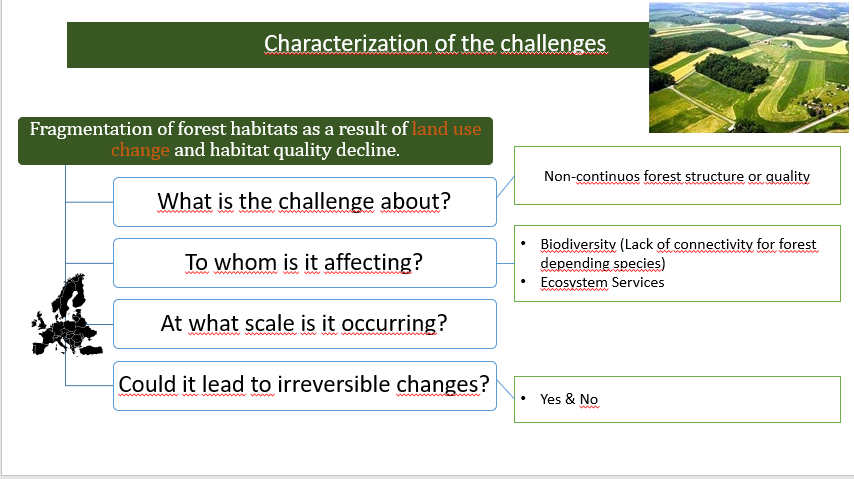


| What is the challenge about? | - Regarding biodiversity conservation: forest dependent species are not connected because a) quality is not sufficient / b) current geographic location of forests is not optimal - Reasons: Land use pressure = infrastructure, urbanization, agriculture (not in southern/central Europe); currently exists, but mainly driven by previous land use decisions |
| --- | --- |
| To whom is it affecting? | - biodiversity (lack of corridors, biol. Stepping stones, reduced connectivity, increasing edge effects) - ecosystem services (particularly cultural, regulating) |
| At what scale is it occurring? | European |
| Could it lead to irreversible changes? | - Biodiversity: Yes, if corridors are lost for certain species, these species have no chance to „have“a habitat there, in the worst case: extinction; for others it can be reversible (wolf/…,)  - ES: for some cultural es it could have a pos. Effect on a local level (->ask Liisa T.) |

- Challenge 2: Increasing frequency and intensity of extreme events in climate variables (water, temperature) causing droughts, changes in water regimes and floods, loss of protection-function


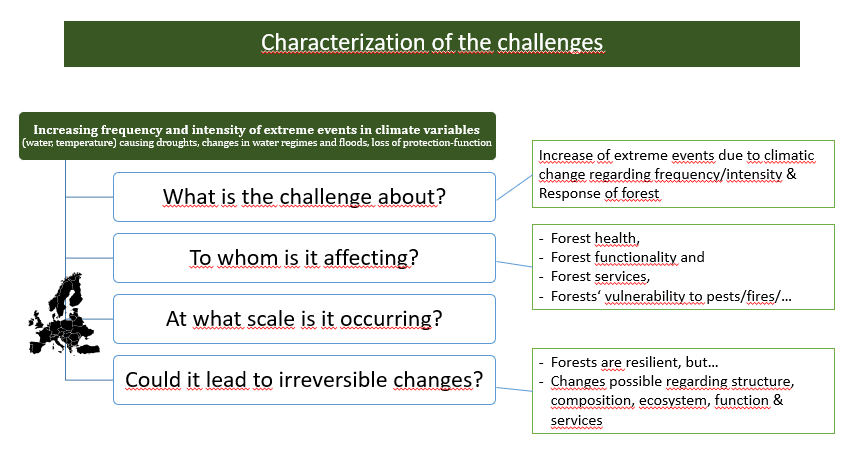


| What is the challenge about? | Increase of extreme events due to climatic change regarding frequency/intensity and response of forest to climatic variability  -> droughts, rainfall |
| --- | --- |
| To whom is it affecting? | - Forest health, - Forest functionality and - Forest services, - Forests‘ vulnerability to pests/fires/… |
| At what scale is it occurring? | European |
| Could it lead to irreversible changes? | Forests are resilient, but there could be changes in structure, composition, ecosystem and thus functions/services  (depends on time scale: First years to 30 years to hundreds…)  Changes are depending on time-scale |

- Challenge 3: Increasing extension, frequency and impacts of pests and invasive species such as the bark beetle infestation.


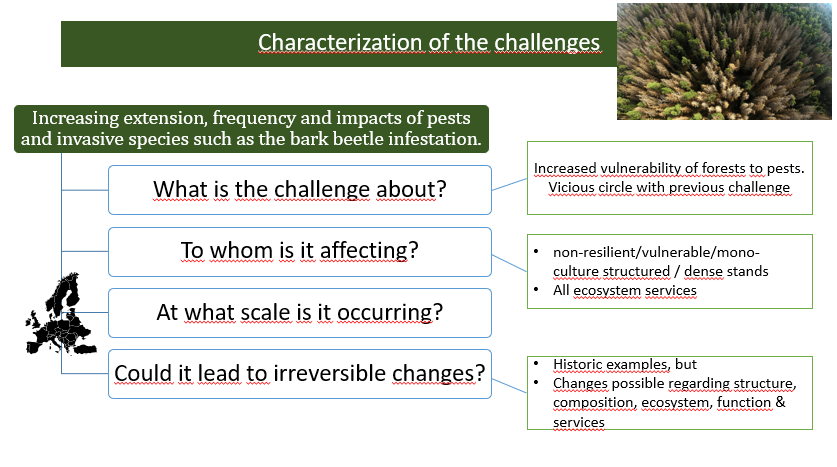


| What is the challenge about? | Increased vulnerability of forests to pests  Consequence of previous challenge and -> vicious circle, will increase vulnerability droughts/…. Highly interlinked |
| --- | --- |
| To whom is it affecting? | - non-resilient/vulnerable/mono-culture structured , dense stands, that are often characterized by a decrease of trad. management - All ecosystem services (carbon sequestration, economic, not protecting soil,…) |
| At what scale is it occurring? | European |
| Could it lead to irreversible changes? | mostly not (except: Dutch elm disease in the medium future), historic examples for such pests  Might result in changes to forest structure/landscape |

- Challenge 4: Degradation of forest soil quality and soil loss and its effect on the capacity to supply FES (acidification, increased nitrogen deposition, etc.)


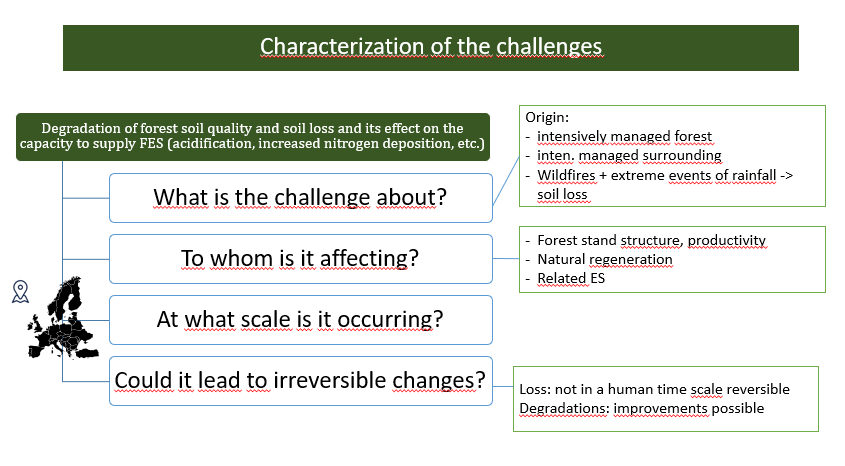


| What is the challenge about? | - Region/context/management dependent - Output of biomass due to extensive management - Input of nitrogen from agricultural sources which leads to a high occurrence of certain plants - Soil loss -> impact on recovery of forest after a fire   Origin:   - intensively managed forest - inten. managed surrounding - Wildfires + extreme events of rainfall -> soil loss |
| --- | --- |
| To whom is it affecting? | - Forest stand structure, productivity - Natural regeneration - Related ES |
| At what scale is it occurring? | locally but throughout Europe |
| Could it lead to irreversible changes? | Loss: not in a human time scale reversible  Degradation: Depending on region and specific conditions, improvements possible |

- Challenge 5: Homogenization of forest stands forests across Europe


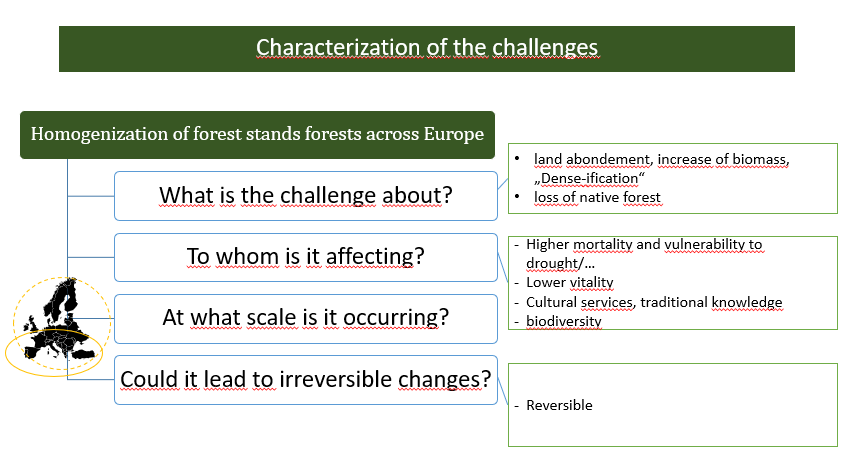


| What is the challenge about? | - Reasons: land abondement, increase of biomass, loss of native forest - „Dense-ification“ |
| --- | --- |
| To whom is it affecting? | - Higher mortality and vulnerability to drought/… - Lower vitality - Cultural services, traditional knowledge - biodiversity |
| At what scale is it occurring? | - Southern Europe - could be a future problem in rest of Europe (especially generational change in private forest ownership, loss of traditional management and related services) |
| Could it lead to irreversible changes? | Reversible |

Exploring relationships between challenges and FES categories and themes:


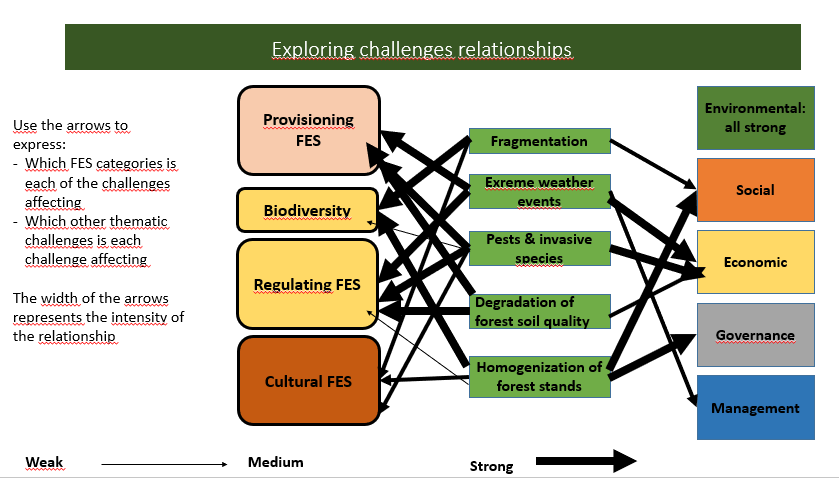


### Group 2 – SOCIO-CULTURAL

- Challenge 1: Changing connection and diverging perceptions and values on forest from society


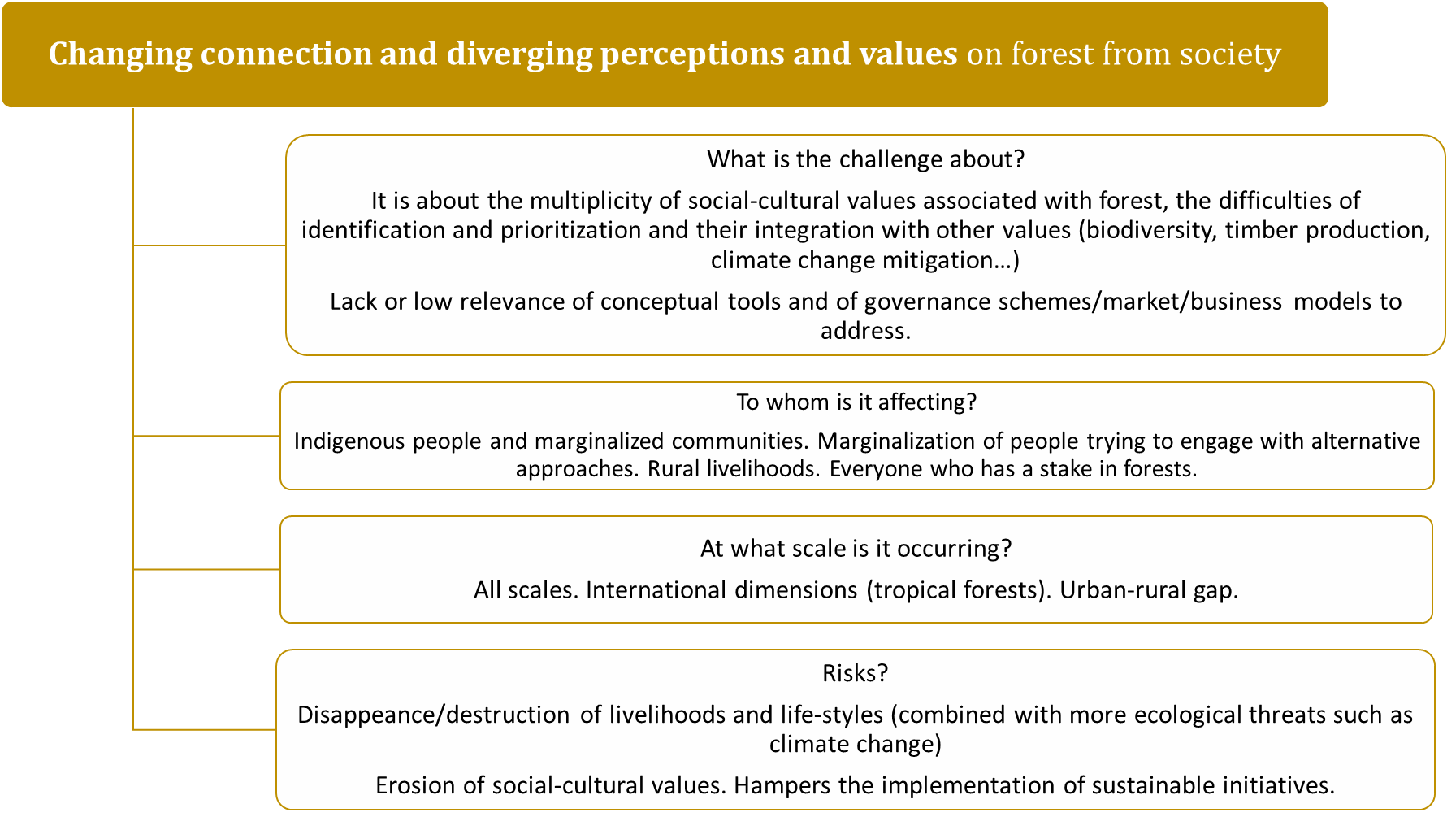


What is the challenge about? It is about an urban-rural; diverging perceptions the role of forest management in maintaining multiple FES (different nature conservation paradigms), diverse cultural values.

*Teresa mentioned* that the understanding of what forest/forestry means is important and changes: it has been subordinated to timber production, but now there is more emphasis on conservation (carbon, old-growth forests) and FES, multifunctionality. Expectations/demands are getting more diverse. Productive forests – recreation activities, ….

Bioeconomy is playing a more important role (mitigation, carbon storing), increasing interest for biodiversity from more sectors of society than conservation organization.

It is about how communities (rural or urban) identify and prioritize the FES. Integration of social-cultural values with biodiversity, climate change, ecological and economic values.

Ths challenge is related to the difficulty to describe what people value in the forest, and as well to know how do you manage forest for e.g. sense of place, how to Integrate of social-cultural values. Values of forests are often in the cultural ES.  there are not many models, markets, governance schemes to address them.

Misrecognized voices in society – indigenous knowledge and values. Any tensions around these questions – marginalization of people trying to engage with alternative approaches

- Challenge 2: Rural depopulation and loss/decrease of social capital in rural areas. 🡪 might be solved as consequence of tackling another challenge


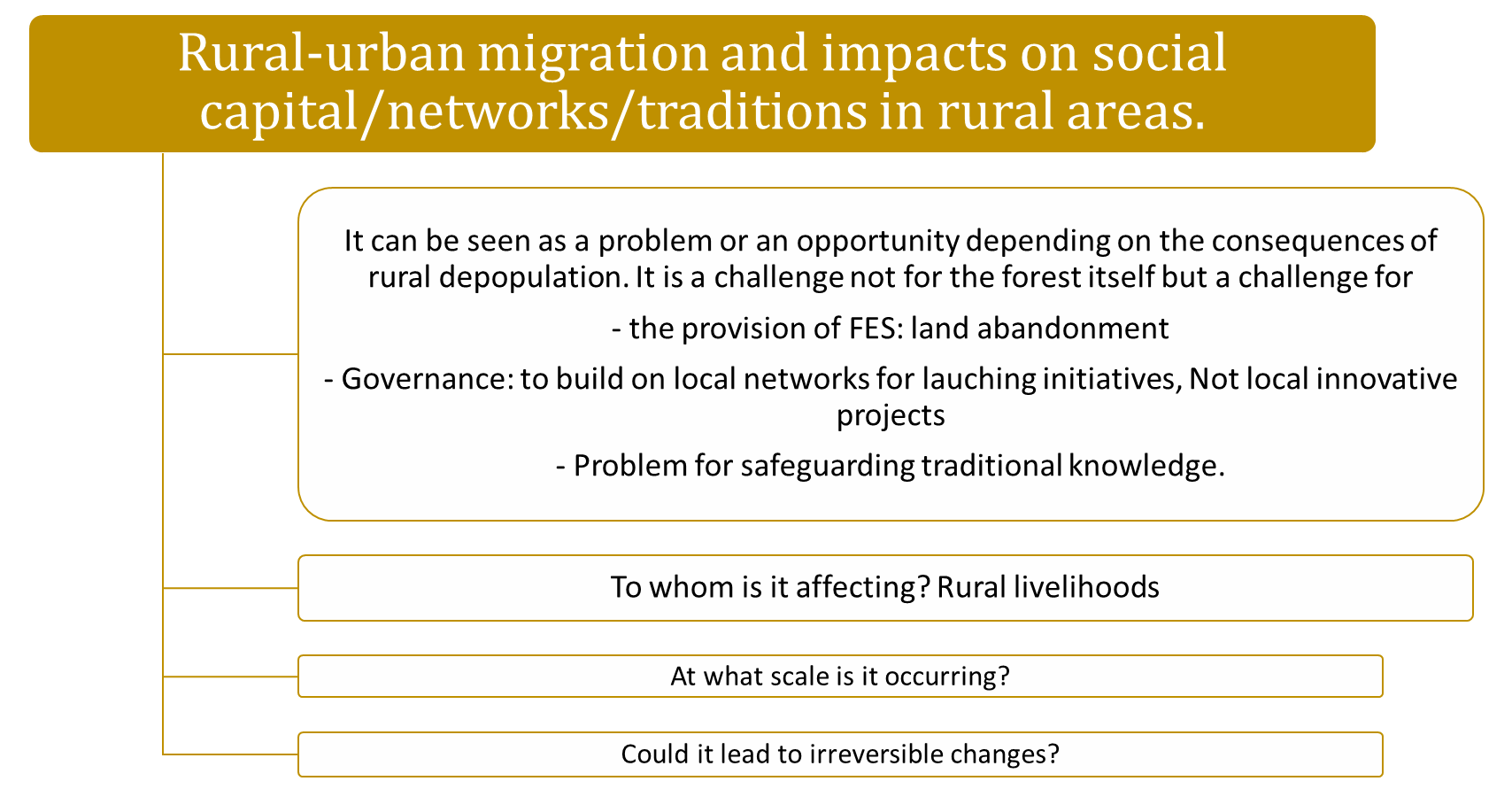


Rural areas are losing populations 🡪 what is the threat? Are traditional forms of management lost?

Loss of landscape value, business and wood value chain

Challenge for governance to build on local networks for launching initiatives, not local innovative projects

Problem for safeguarding traditional knowledge.

If e.g. mountain farming is abandoned, the esthetic value for tourism will decrease 🡪 it triggers the need for maintenance of cultural landscape.

In this challenge, there can also be seen a countermovement: urban people moving to the country side. They have other priorities (side-activity, not so dependent on it to make a living so they are more open to taking risks), other knowledge and skills.

- Challenge 3: Lack of generational turnover in the forest sector and (or leading to) shifts/abandonment of forest management. 🡪 after internal discussions, it was decided by the group that this challenge can be subsumed into the previous one, on rural depopulation.
- Challenge 4: Fragmented knowledge on managing multiple FES


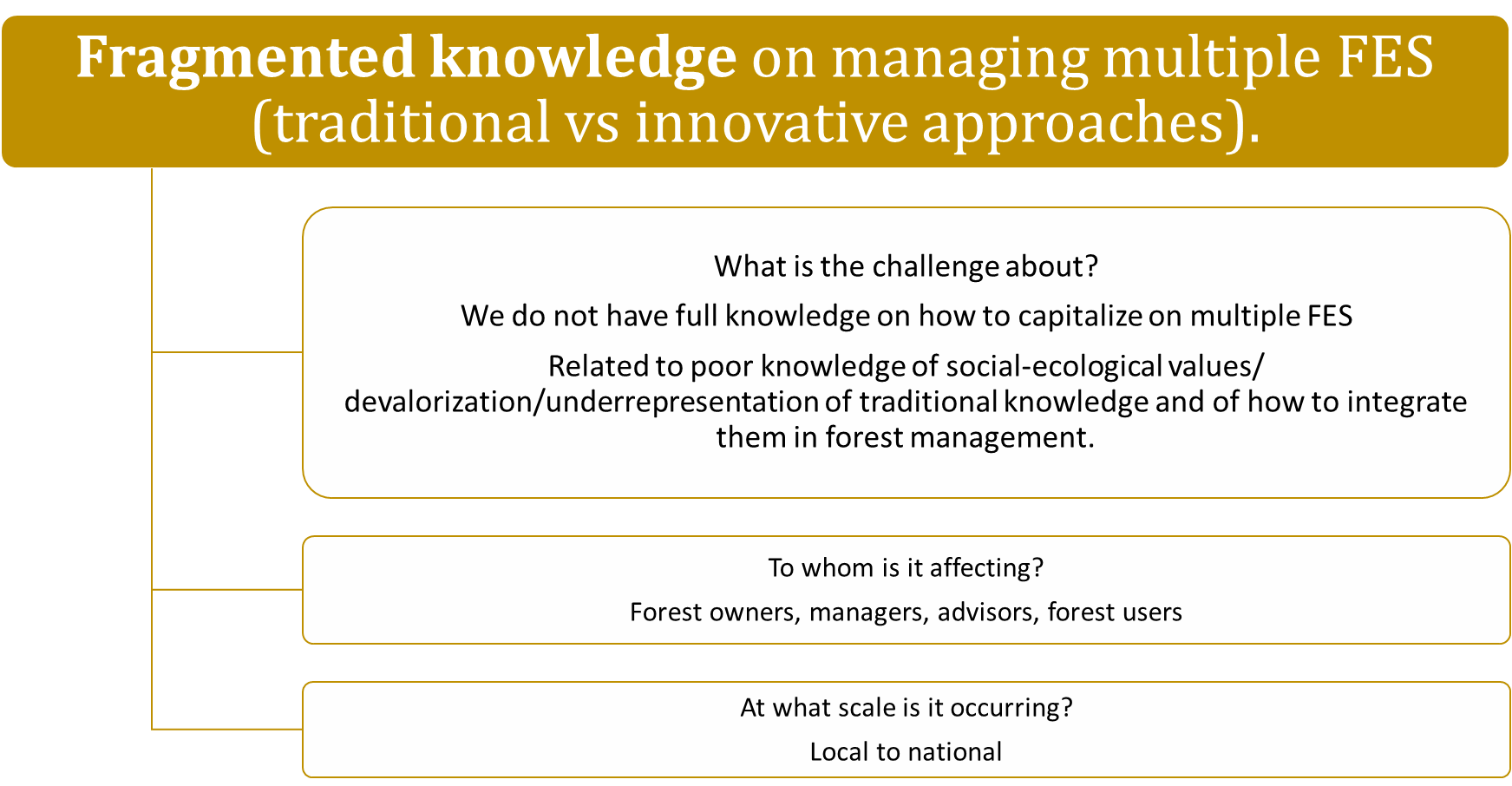


Unbalanced consideration of traditional knowledge/devaluation/underrepresentation of traditional knowledge.

Related to poor knowledge of social-ecological values and of how to integrate them in forest management

Link between values and practices.

When you hire someone to manage your forest, they do it with a productive mind-set, not related to multiple ES.

- Challenge 5: Conflicts between FES providers and FES beneficiaries (power asymmetries) - Unclear relationship between property rights and FES as common goods


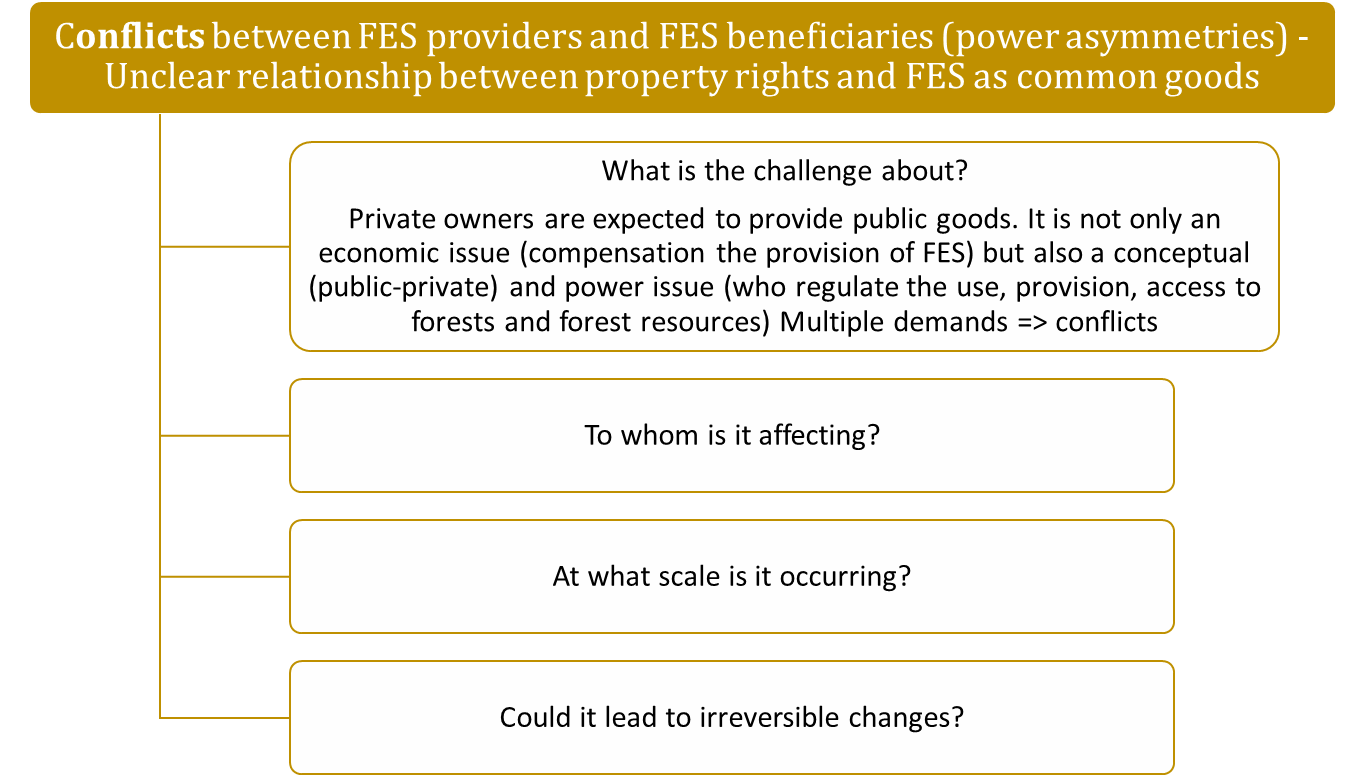


In relation to the relationships between the social challenges, FES categories and other thematic areas, there was not enough time to finish the work.

### *Group 3 – ECONOMIC*

- Challenge 1: Lack/inefficiency of financial tools and support to cover losses from, and adaptation towards natural hazards (e.g. pests, extreme weather events, etc.).

What is the challenge about?

- increasing frequency and magnitude of natural hazards
- Partial new phenomena (new pests, fires in the north of Europe, dying of Beech)
- High risk and high losses in FM
- Lack of information and usability of tools
- New long-term risk management needed

To whom is it affecting? Private and public forest owners, administration,

What is the scale of the challenge? More local to regional effects

Irreversible changes? n economic terms it may lead to irreversible changes (in ecological terms it is)

- Challenge 2: Lack of economic instruments (incentive based policies, PES) and business models for the recognition and promotion of regulating and cultural FES as well as non-wood-forest-products (particularly those of public good character) provided by forests in Europe.

What is the challenge about?

- There are not many incentive-based instruments implemented
- Few business models and schemes existent (lack of knowledge and niche businesses)
- Difficult to upscale and replicate
- Economic rational is increasingly contested
- Ecological efficiency and economic sustainability is questionable (voluntary)

To whom is it affecting? FES providers (i.e. forest owners)/users, entrepreneurs, society, NGOs,

What is the scale of the challenge? Implementation on local and solutions/institutional setting on state/national level

Irreversible changes? No, it’s reversible

- Challenge 3: Low profitability of multifunctional and close to nature forestry models (SFM) in Europe in relation to intensive timber production models

What is the challenge about?

- Higher opportunity costs and potentially higher management costs
- Less revenue
- Externalising the risk of timber loss to public funding/burden on society (i.e. storms, bark beetle)
- Externalising intensive timber production to other countries/outside Europe

To whom is it affecting? Private and public forest owners, entrepreneurs, society, NGOs

What is the scale of the challenge? Local to European/global

Irreversible changes? Irreversible in the case of conversion of primary forests

- Challenge 4: Economic power asymmetries in the forest sector dominated and influenced by a reduced number of actors (forestry regime, wood processing industry, other powerful sectors such as bioeconomy/bioenergy/biofuel), who control and direct the markets towards intensive biomass and wood/timber production regardless of the negative externalities

What is the challenge about?

- Closed circle of forestry actors with high path dependency
- Many unorganized small forest owners
- Less tradition of participatory decision-making process (setting objectives)
- Energy/agr: less economic power of forestry
- Non-recognition of externalities by industry
- Hinders forest owners to apply MF/FES orientated management (beyond timber production)

To whom is it affecting? Private forest owners on all scales, outside the forest sector as well

What is the scale of the challenge? Very local to national level within the forestry sector, influences (i.e. international trade) can be more on European level

Irreversible changes? Reversible

- Challenge 5: Lack of information/knowledge on societal demand for different FES (+ valuation/WTP/trade-offs

What is the challenge about?

- Lack of systematic studies, assessments, monitoring especial of demand for regulatory and cultural FES
- Methodological challenge, especially in terms of valuation
- Lack of knowledge of interaction of ES
- Trade-offs are often unclear: decisions are fluid and depends on socio-political preferences

To whom is it affecting? Policy makers, society, especially public forest owners

What is the scale of the challenge: local to global

Irreversible changes? No, reversible

As for the relationships between the challenges and other FES categories and thematic areas, it was discussed how all challenges are affecting all three FES and all 5 types of challenges, but with different intensities.


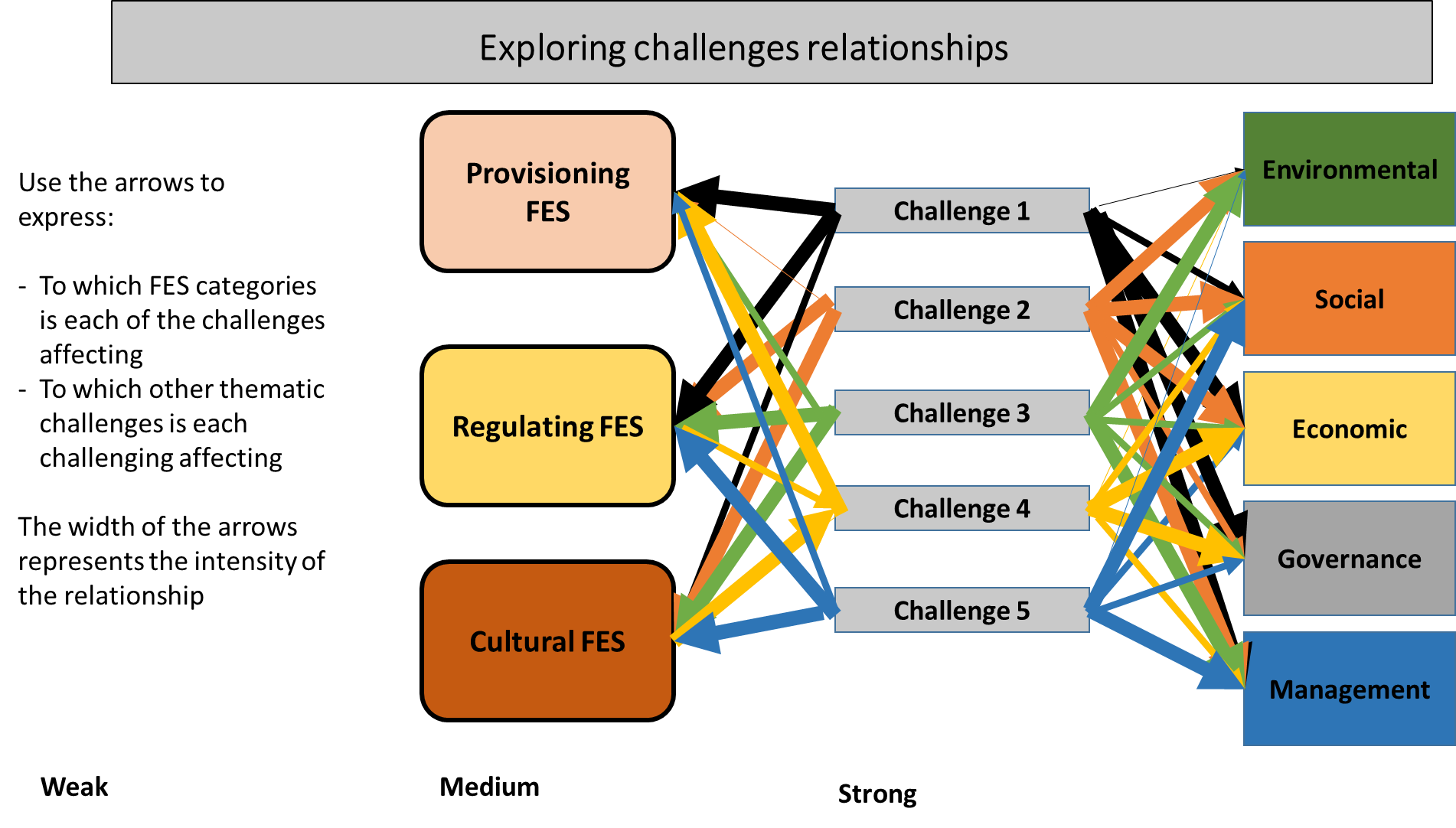


### *Group 4 – MANAGEMENT*

- Challenge 1: Unknown demand and supply of FES for adjust the management strategies accordingly


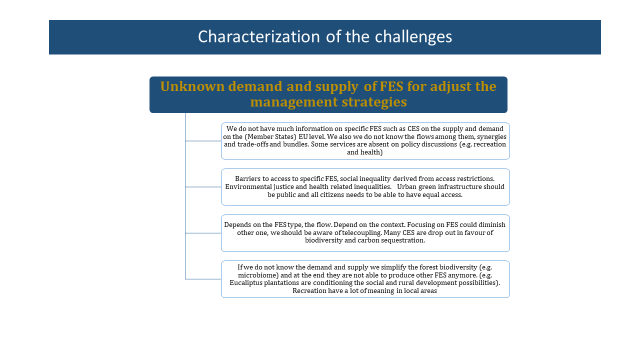


- Challenge 2: Outdated and normative mindset on forest management


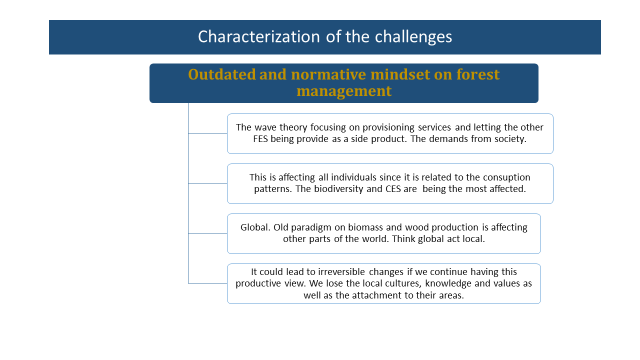


- Challenge 3: Lack of adaptive forest management practices to changing conditions


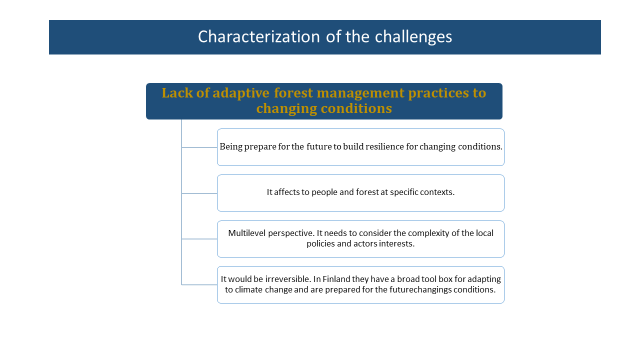


- Challenge 4: Loss of local ecological and traditional knowledge


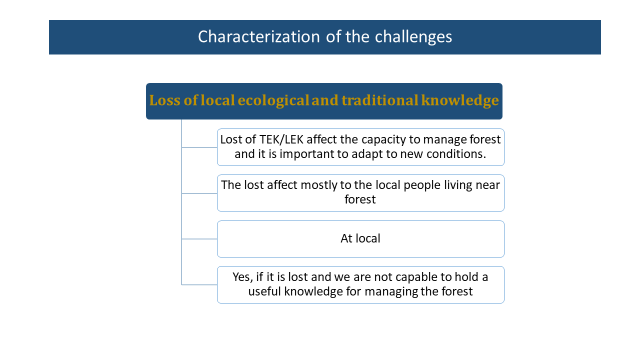


- Challenge 5: General lack of expert support to forest owners, forest operators (other stakeholders)…


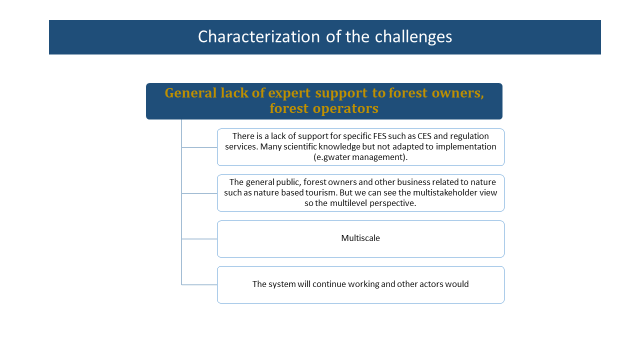


As for the inter-relations between the challenges and the FES categories and thematic areas:


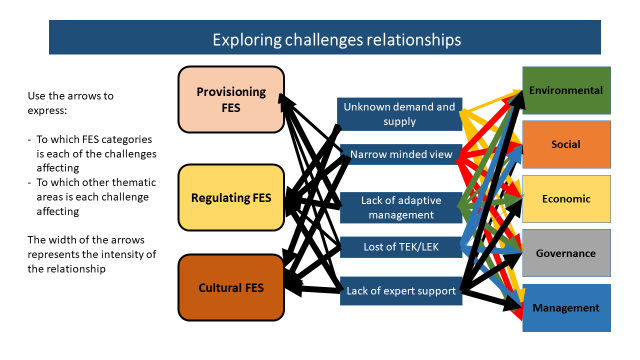


### *Group 5 – GOVERNANCE*

- Challenge 1: Conflict between nature conservation (no management) and resource use (management) as the pathway to provide multiple FES.

Despite its importance, it was commented how this is a challenge that occurs more frequently at an academic level rather than at a management level. Its relevance defining the national policies is however quite high.

The challenge is very associated to the land sharing-land sparing debate.

This challenge is also very asymmetric across Europe. Specifically, in the North and Central Europe there is a tendency of advocating for a low level of management in order to preserve and enhance regulating FES. However, in Mediterranean Europe, that is not an option, as those forest that are not managed end up burned.

- Challenge 2: Lack of coordination and competition among different policy sectors especially those with contradicting policy goals and across administrative levels

This is a challenge that occurs in any policy context, as it is very inherent to human behavior. It affects all FES supply at all levels and in every sector.

It is a problem that has two dimensions:

- Vertical conflicts: across different administration levels (i.e. municipal with regional administration).
- Horizontal conflicts: across different sectors of the administration (i.e. agriculture, forestry, economy, etc.)
- Challenge 3: Lack of participatory governance (who, when and why to include) and cooperative approaches to provide multiple FES

This is a challenge that intersects with all the previous challenges and is inherently related to the power asymmetries and inequalities of each context. It is also a challenge that affects almost everywhere at a local scale.

It is a challenge very much related to the lack of bottom-up participation. Something that particularly lacks in the forest sector (although there are cases where that does not happen). It does not only mean lack of citizen participation, but also participation from different sectors with different expertise.

- Challenge 4: Heterogeneous and unequal forest ownership structures, related to land tenure and forest size, obstructing policy implementation for the sustainable provision of multiple FES

It is a challenge that refers to the fact that Many European forest areas span over large areas with different ownership systems and property rights.

The heterogeneity in this case, can be seen more as an opportunity rather than a challenge. It is also a challenge that relates more with economic aspects than environmental (although of course, everything is inter-related) 🡪 It affects more timber policies than conservation policies.

- Challenge 5: Lack of integrated landscape approaches coordinating forest owners’ (and other ecosystems) decisions. Need of concerted management and policies guiding forest owners’ actions at landscape scale.

This is a challenge very much related to the lack of participatory governance. It is specially relevance for the lack of coordination with agricultural practice (for example the inclusion or exclusion of grazing in forests). This challenge has a focus on a small scale (municipal/community).

- Challenge 6: Tensions and conflict of interests in public forests between its role as a strategic profitable resource on the one hand, and as public good with the mandate to provide multiple FES on the other hand.

This is a very tricky challenge as it is very ideological. It related to how public resources should be managed. Public forests account for around half of European forests. There is a conflict of interests between the public demand and mandate for these forests to provide multiple FES. On the other hand, forests are a potentially profitable resource, so there is a tendency of management for that purpose with large negative environmental externalities. Another side of this coin happens when these forests are not profitable, which leads to their abandonment or mismanagement, which also has very negative effects (i.e. wildfires).

- Challenge 7. Significant proportion of European forests is owned by small forest owners (in many cases absent), difficult to engage in any forest strategy

This challenge is to some extent included in challenge 4, but also to the third. Small forest owners account for a significant part of European forests. Nowadays, it happens that land tenure is very fragmented and forest owners, particularly small forest owners, in many occasions don’t live in the area and/or are not interested in the management of their forests. To engage them in any kind of program designed to boost multiple FES is very challenging due to the lack of interest, incentives and knowledge.

Inter-relation of challenges with FES categories and thematic areas:


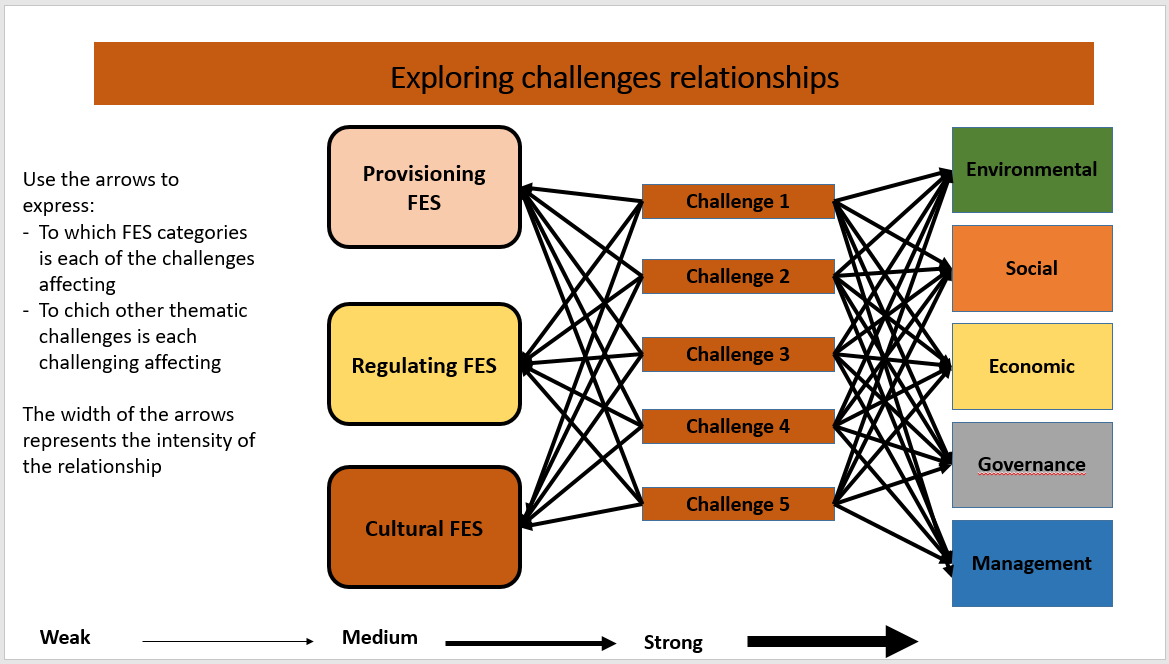


Governance is the mean to an end, not the final objective. How they relate with other items depends on the matter over which each specific policy relates. Therefore, it was discussed that all challenges interact deeply with all categories

# **Part III: Plenary and selection of top 3 challenges per area**

Each group presented the materials exposed above. After each of the presentations, an online voting was held. These are the results of the voting (in green are the top 3 challenges).

Environmental

| Challenge | % |
| --- | --- |
| 1. Increasing frequency and intensity of extreme weather events in climate variables causing droughts, changes in water regimes and floods, loss of protective function | 95 |
| 1. Fragmentation of forest habitats as a result of land use change | 68 |
| 1. Increasing extension, frequency and impacts of pests and invasive species such as the bark beetle infestation | 47 |
| 1. Homogenization of forest stands across Europe (Taken by Management group) | 47 |
| 1. Degradation of forest soil quality and soil loss and its effect on the capacity to supply FES (acidification, increased nitrogen deposition, etc.) | 42 |

There was a tie between two challenges. The discussion that came afterwards pointed to the selection of challenge 3, as challenge 4 would be to a large extent covered by some of the themes covered in the management group.

Social

| Challenge | % |
| --- | --- |
| 1. Conflicts between FES providers and FES beneficiaries (power asymmetries) - Unclear relationship between property rights and FES as common goods | 89 |
| 1. Changing connection and diverging perceptions and values on forest from society | 83 |
| 1. Rural-urban migration and impacts on social capital/networks/traditions in rural areas - Including lack of generational turnover in the forest sector and (or leading to) shifts/abandonment of forest management | 67 |
| 1. Fragmented knowledge on managing multiple FES (traditional vs. innovative approaches) | 62 |

Economic

| Challenge | % |
| --- | --- |
| 1. Lack of economic instruments and business models for the recognition and promotion of regulating and cultural FES as well as non-wood forest products (particularly those of public good character) provided by forests in Europe | 89 |
| 1. Economic power asymmetries in the forest sector dominated and influenced by a reduced number of actors, who control and direct the markets towards intensive biomass and wood/timber production regardless of the negative externalities | 74 |
| 1. Lack of information/knowledge on societal demands for different FES (+valuation/WTP/trade-offs) | 47 |
| 1. Lack/inefficiency of financial tools and support to cover losses from, and adaptation towards natural hazards (e.g. pests, drought, fires, ...extreme weather events, etc.) | 47 |
| 1. Low profitability of multifunctional and close to nature forestry models (SFM) in Europe in relation to intensive timber production models | 37 |

There was again a tie between challenge 3 and 4. In the discussion that came afterwards, it was observed that challenge 3 would be covered by the management group.

Management

| Challenge | % |
| --- | --- |
| 1. Unknown demand and supply of FES for adjust the management strategies accordingly (take the Economic Challenge: Lack of information/knowledge on societal demands for different FES (+valuation/WTP/trade-offs) | 68 |
| 1. Outdated and normative mindset on forest management | 68 |
| 1. Lack of adaptive forest management practices to changing conditions (Homogenization of forest stands across Europe) | 68 |
| 1. Loss of local ecological and traditional knowledge | 42 |
| 1. General lack of expert support to forest owners, forest operators and other stakeholders related to forest management | 47 |

Governance

| Challenge | % |
| --- | --- |
| 1. Lack of coordination and competition among different policy sectors, especially those with contradicting policy goals and across administrative levels. | 95 |
| 1. Lack of participatory governance and cooperative approaches to provide multiple FES (who, when and why to include). | 70 |
| 1. Tensions and mismatching expectations about the role of public forest either as a strategic profitable resource on the one hand and/ or as public goods with the public mandate to provide FES on the other hand. | 50 |
| 1. Heterogeneous and unequal forest ownership (structure related to property rights, land tenure and forest size) obstructing policy implementation for the sustainable provision of multiple FES. | 40 |
| 1. Conflicts between nature conservation and resources use as the pathway to provide multiple FES. This conflict at a local level is related to diverging forest uses (Hunting recreation) | 35 |

# **Day 2: Identifying the Strategic Solutions**

## **Part IV-V: Identification and characterization of the strategic solutions**

### *Group 1 - ENVIRONMENT*

- Challenge 1: Increasing frequency and intensity of extreme weather events in climate variables causing droughts, changes in water regimes and floods, loss of protective function


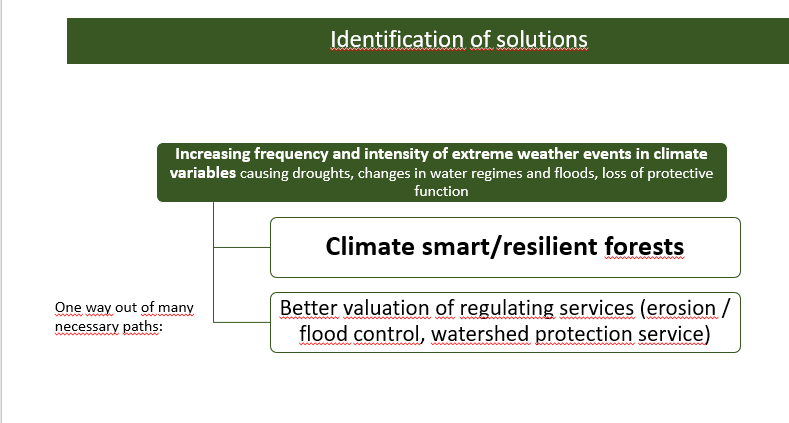


Comments we received:

- assist migration of species adapted to future climatic conditions

Own discussion:

- Adaptive management/planning works for all challenges
- Climate smart forest (several papers): What do we mean with it?

<https://efi.int/articles/climate-smart-forestry>

<https://www.sciencedirect.com/science/article/pii/S1389934120300630>

Policy paper with three main areas of action:

3.1. Enhance global afforestation and avoid deforestation and degradation

3.2. Combine mitigation and adaptation measures in the management of forests

3.3. Use wood sustainably and substitute non-renewable carbon-intensive materials

- Use models on how species distribution changes
- 2nd solution? Change current mind-set to uneven aged, resilient.
- Low productive areas can be very resilient.
- Better valuation of regulating services (erosion / flood control, watershed protection service) -> incorporate in regional forest management, will be in focus here as one path out of many possible strategies
- Strategic/policy level: commitment of countries to develop schemes for regulating services


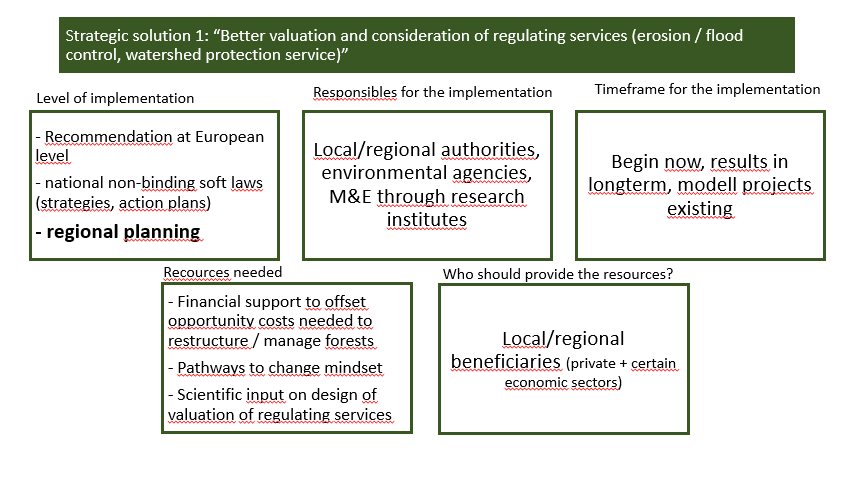


Characterization of the solution

- Resistant species
- Uneven Age
- Mixed Forets
- multifunctional
- Change mindsets of responsible persons (-> uneven aged forest stands, multiple species…)
- Include „new“ species (that are not invasive), ensure mixed forests

What is the right path?

- Interfere or let nature take its course?
- Region specific solutions

Level of implementation

- Legally: national state? -> implemented regional

Change of mindset necessary?

- Most difficult and long-term, in forestry mainly through generational change
- Recent years: mind set change started. Multifunctional is more prominent
- Societal perception on climate change and influence on forestry is increasing

Responsibilities

- depends on the way of implementing: legislation/voluntary/incentive/market-based
- Example: new york: council agreed to use regional tax money to forest managers
- Conclusion: Each local region has to decide on their own, national-non binding support
- Certain economic sectors -> for example ski industry benefits if less avalanches/…

Other important characteristics

- Need to link research and practice
- During decision process: Participation necessary
- Evaluation through science
- Financial support to offset opportunity costs needed to restructure forests, afforestation
- Spatial mismatch between supply and demand of regulating services (global scale for regulation, and local „demander“ do not care too much)

Other possible strategy: Cultural services and valuation -> traditional management -> resilience

- Challenge 2:


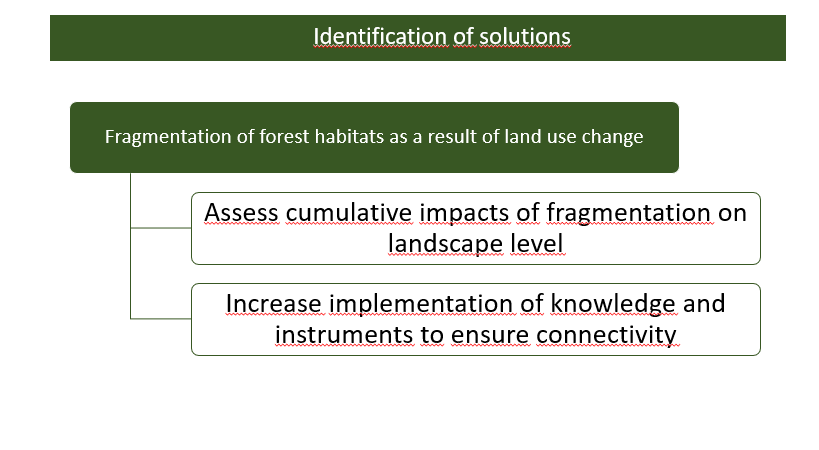


Here: Focus on physical fragmentation of forest patches (reduction of connectivity), not differences regarding habitat quality

Comments we received:

- possible solutions: create ecological corridors, replant forests were possible, prevent with proper plans instruments

Own discussion:

Identify areas with lack of connectivity (much data exists on European level)

Problem: Forest area or quality? Local level land use planning (local fragmentation, distribution)

Improve use of fragmentation in ES assessment

Ecosystem service „connectivity„ is not considered enough -> include in ES framework

Local planning instruments and knowledge is there, just not implemented (Eastern Europe. What about rest?)

Germany: Natura 2000 areas are not yet „secured „everywhere

EA = Environmental Assessment

Account not only locally

Long version: Overview of large scale and cumulative impacts of fragmentation via strategic EA that consider multiple local-level decisions

### Group 2 – SOCIO-CULTURAL

- Challenge 1: Conflicts between FES providers and FES beneficiaries (because of diverging interests, demands and rights).


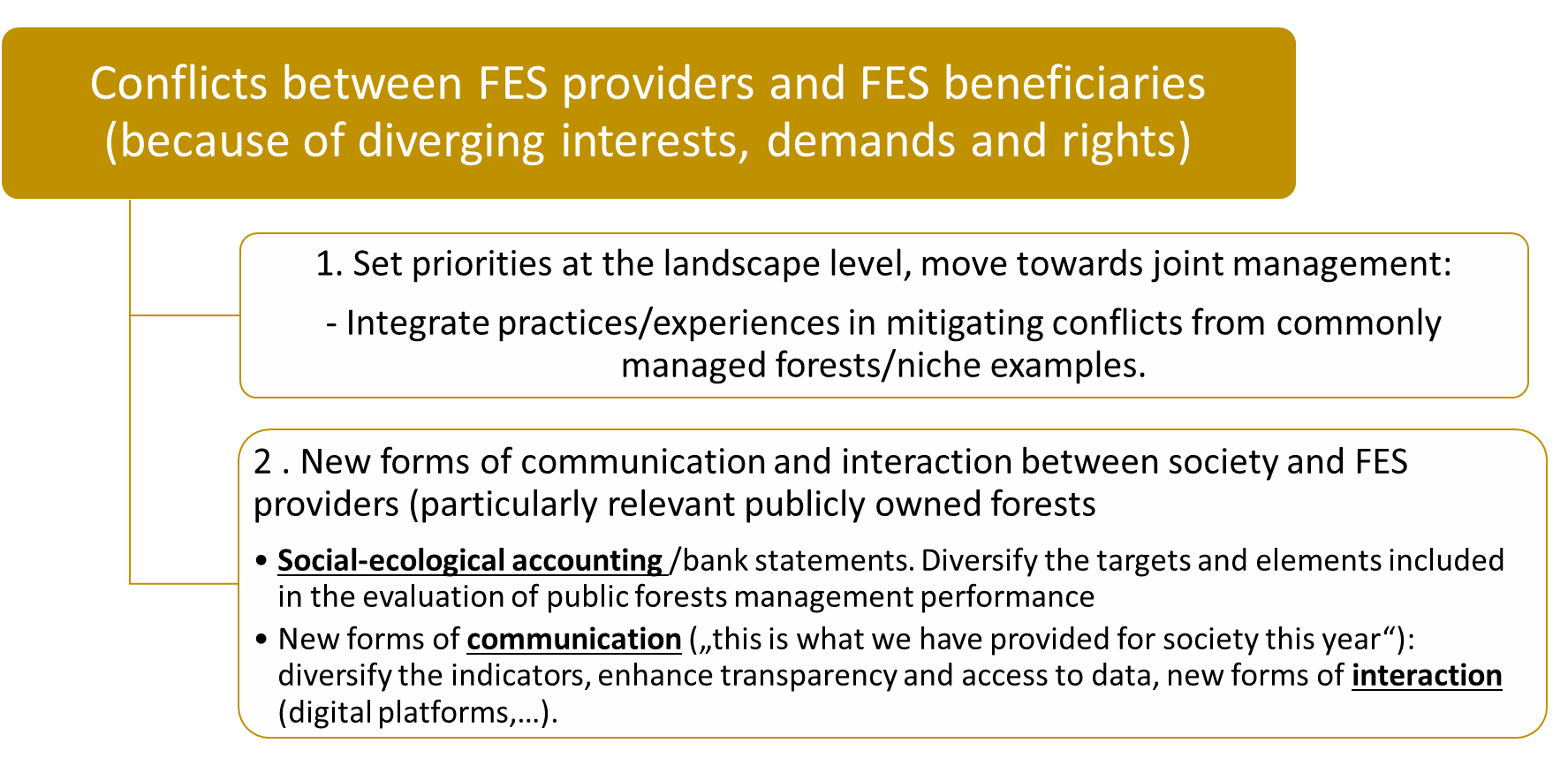


There would be two strategic solutions for this challenge:

1. Solution 1. Set priorities (Identify and integrate FES demands) at the landscape level, move towards joint management:

Integrate practices/experiences in mitigating conflicts from commonly managed forests/niche examples.

It is related to an unclear relationship between property rights and FES as common goods. (FES public goods/ decision power asymmetries) -

It is a Governance challenge:
There are tensions and mismatching expectations about the role of public forest either as a strategic profitable resource on the one hand and/ or as public goods with the public mandate to provide FES on the other hand. Diverging interests and demands between FES providers and beneficiaries, which lead to conflicts (Conflicts of rights)

Different demands and interests often corrugate with the rules of forest governance (property rights)

Identification of solution:

Different types of property regimes

1. For private forests: set priorities at the landscape level, move towards joint management. Incentive schemes that foster management at landscape levels (agro-environmental schemes), targets from the state and freedom to choose.
2. For public forests: should have clear role in being more experimental and focus on public good, recognizing the importance of FES
3. For common property (different across Europe: municipality / local communities / groups of farmers…): preserving them. Practices from commonly managed forests 🡪 into public forests management. Integrate experience made in mitigating the conflicts
4. Open-access forests
5. Solution 2: New forms of communication and interaction between society and FES providers (particularly relevant publicly owned forests) through:

- Social-ecological accounting /bank statements. Diversify the targets and elements included in the evaluation of public forests management performance
- New forms of communication („this is what we have provided for society this year“): diversify the indicators, enhance transparency and access to data, new forms of interaction (digital platforms…).
- Challenge 2: Changing relation with and diverging perceptions and values on forests by society


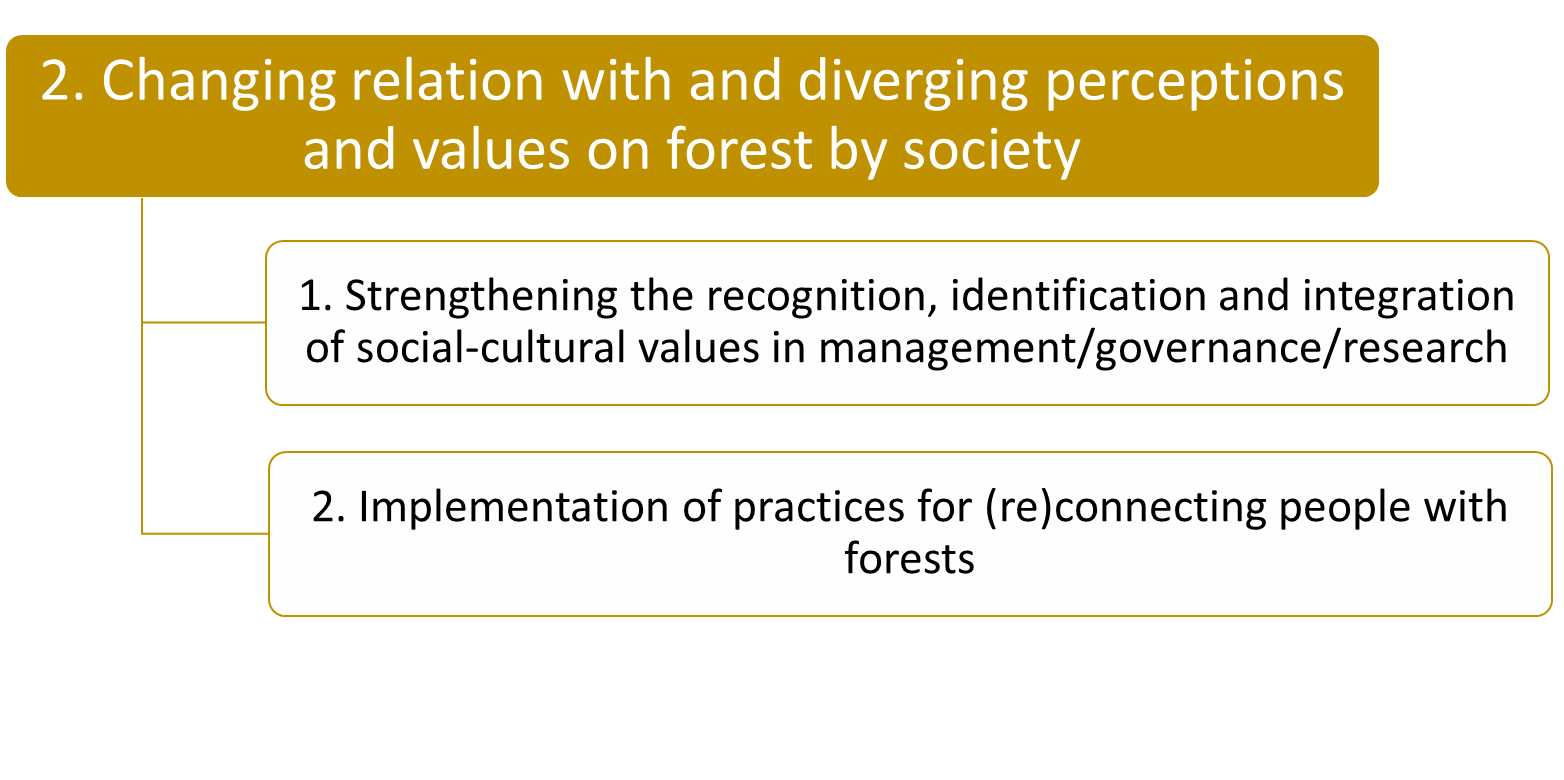


Identification of solution:

1. Solution 1. Strengthening the recognition, identification and integration of social-cultural values in management/governance/research

- Better integrate justice dimension (justice as recognition) 🡪 target for policy) Focus on parts of society that are less recognized, respected, included.
- Making forest management more inclusive. Not only inclusion of western scientists (US perspective on forestry – US forest service more diverse, not only forest engineers)
- Strengthening participatory approaches, forest management / co-management: we impose it in the Global South, but still not so well applied in the North. We still lack to social skills.
- Integrate the local community in planning, at local scale


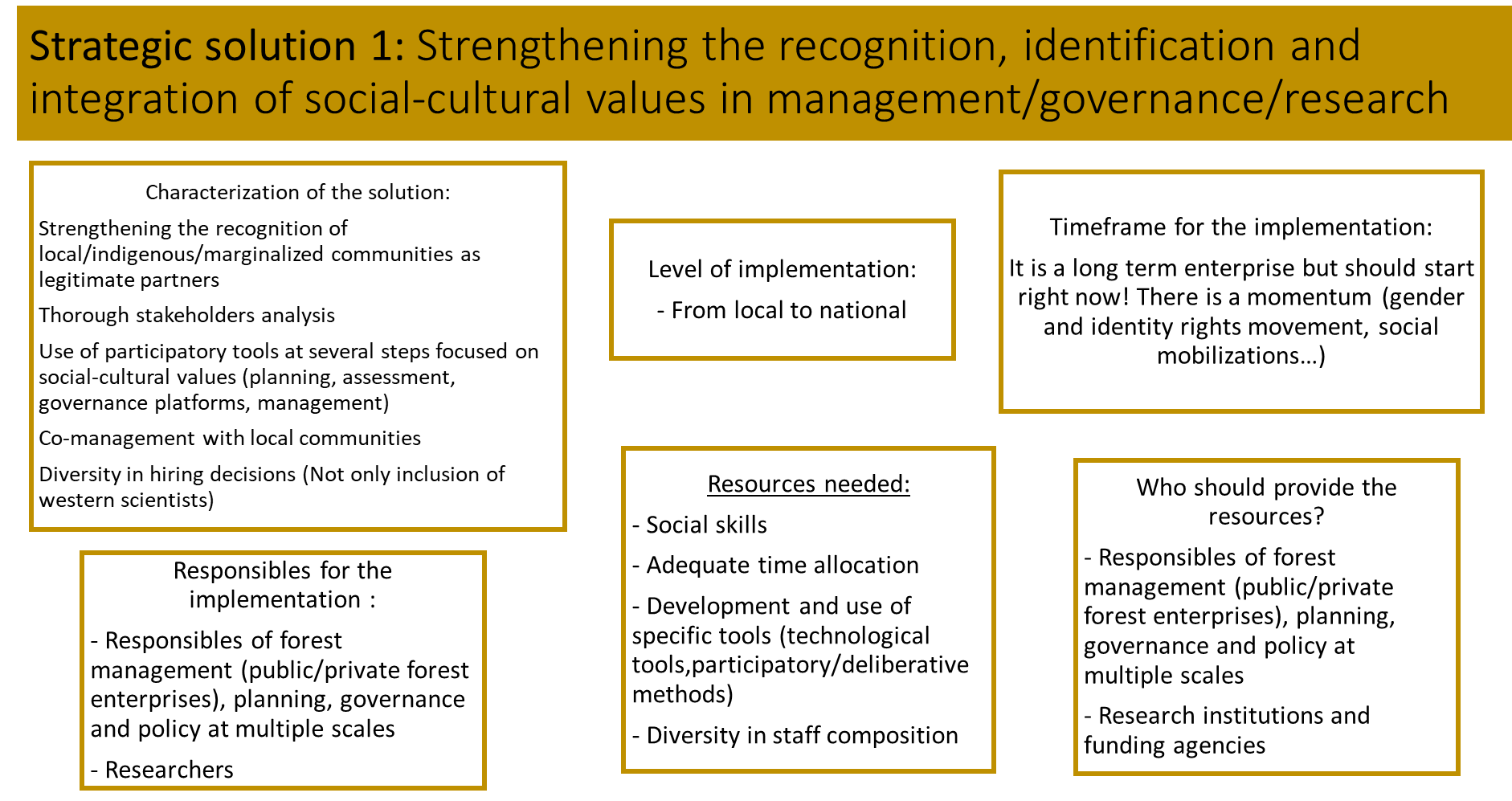


Characterization of the solution:
Strengthening the recognition of local/indigenous/marginalized communities as legitimate partners.

Thorough stakeholders analysis
Use of participatory tools at several steps focused on social-cultural values (planning, assessment, governance platforms, management)
Co-management with local communities
Diversity in hiring decisions (Not only inclusion of western scientists)

Level of implementation:
- From local to national

Responsibles for the implementation :
- Responsibles of forest management (public/private forest enterprises), planning, governance and policy at multiple scales
- Researchers

Timeframe for the implementation:
It is a long term enterprise but should start right now! There is a momentum (gender and identity rights movement, social mobilizations…)

Resources needed:
- Social skills
- Adequate time allocation
- Development and use of specific tools (technological tools, participatory/deliberative methods)
- Diversity in staff composition

Who should provide the resources?
- Responsibles of forest management (public/private forest enterprises), planning, governance and policy at multiple scales
- Research institutions and funding agencies

1. Solution 2: Implementation of practices for (re)connecting people with forests.

If there is a lost or weakening of connection: 🡪 connecting people to forests

- Reconnecting people to forests (kindergarten, forest schools…)
- More people are reconnecting to forest from these spiritual/cultural values, not for livelihoods.

Both approaches merge at certain scales

- Non-material values are underrepresented in ES assessments. Better integrate them already in the assessment. More qualitative social sciences in ES assessment.

Not underestimating the power issues in participation. 🡪 Challenge on power asymmetries

- Challenge 3: Rural migration and impacts on rural areas, including lack of generational turnover in the forest sector and (or leading to) shifts and/or abandonment of forest management


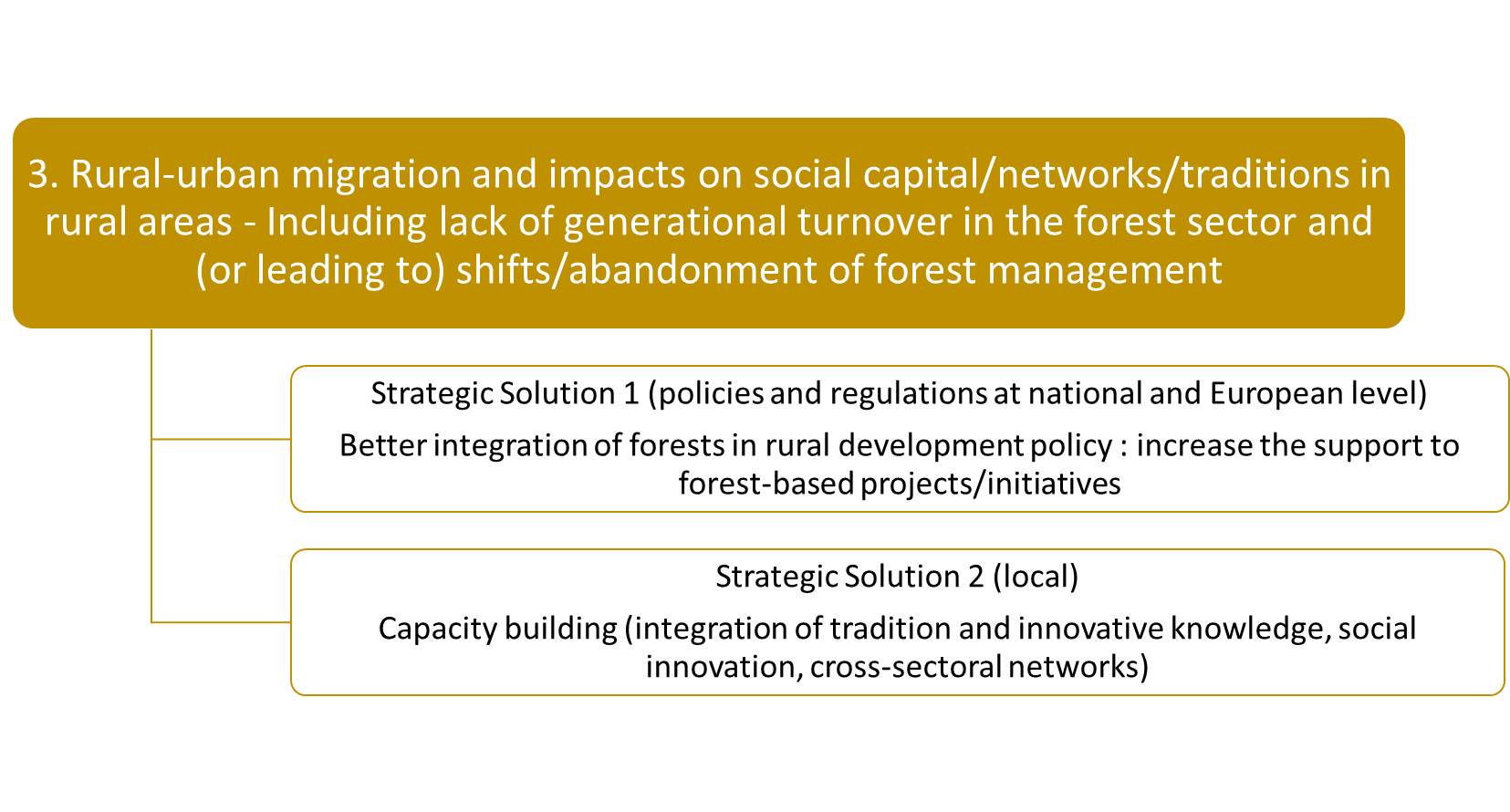


Notes brainstorming Identification of solution:

- Neoruralism = opportunity to integrate urban newcomers in rural networks
- More long distance connection with forests (with digital tools, rural-urban partnerships, collaboration in landscape management actions)
- Increase development funding (EU) to support initiatives/forest-based projects (from national to international) – rural dvlpt projects exist but lack of knowledge, support, experience. Better networks of consultancy
- Capacity building
- Rewarding forest owners who really manage their forests with tax reduction
- Less bureaucracy for small businesses/enterprises in rural areas to encourage them to integrate innovative solutions

Two strategic solutions were identified:

1. Strategic solution 1: better integration of forests in rural development policy: increase the support to forest-based projects/initiatives (through policies and regulations at national and European level)
2. Strategic solution 2: capacity building (integration of tradition and innovative knowledge, social innovation, cross-sectoral networks).

### *Group 3 – ECONOMIC*

- Challenge 1: Lack and inefficiency of economic instruments and business models for the recognition and promotion of regulating and cultural FES as well as non-wood forest products.


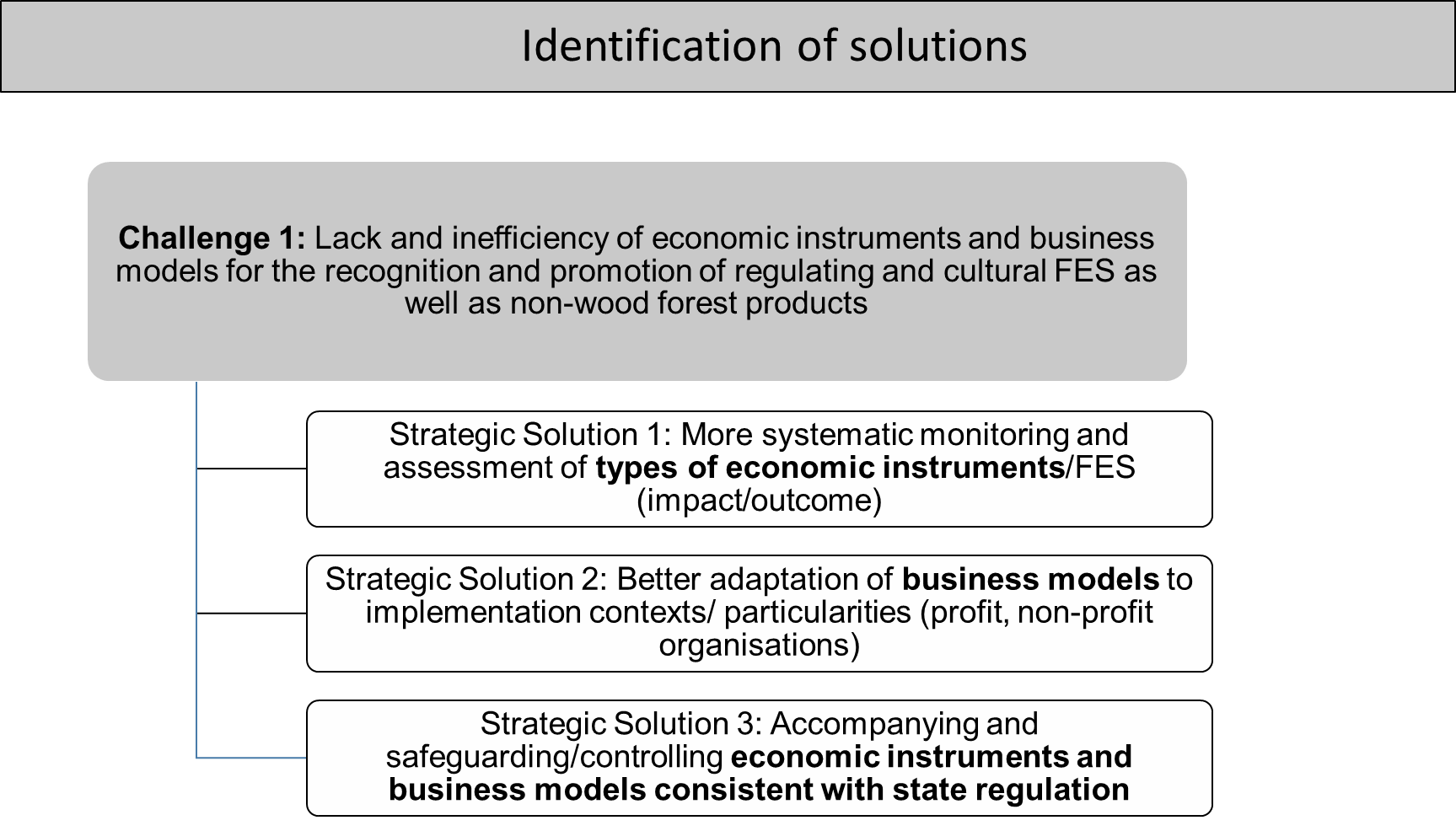

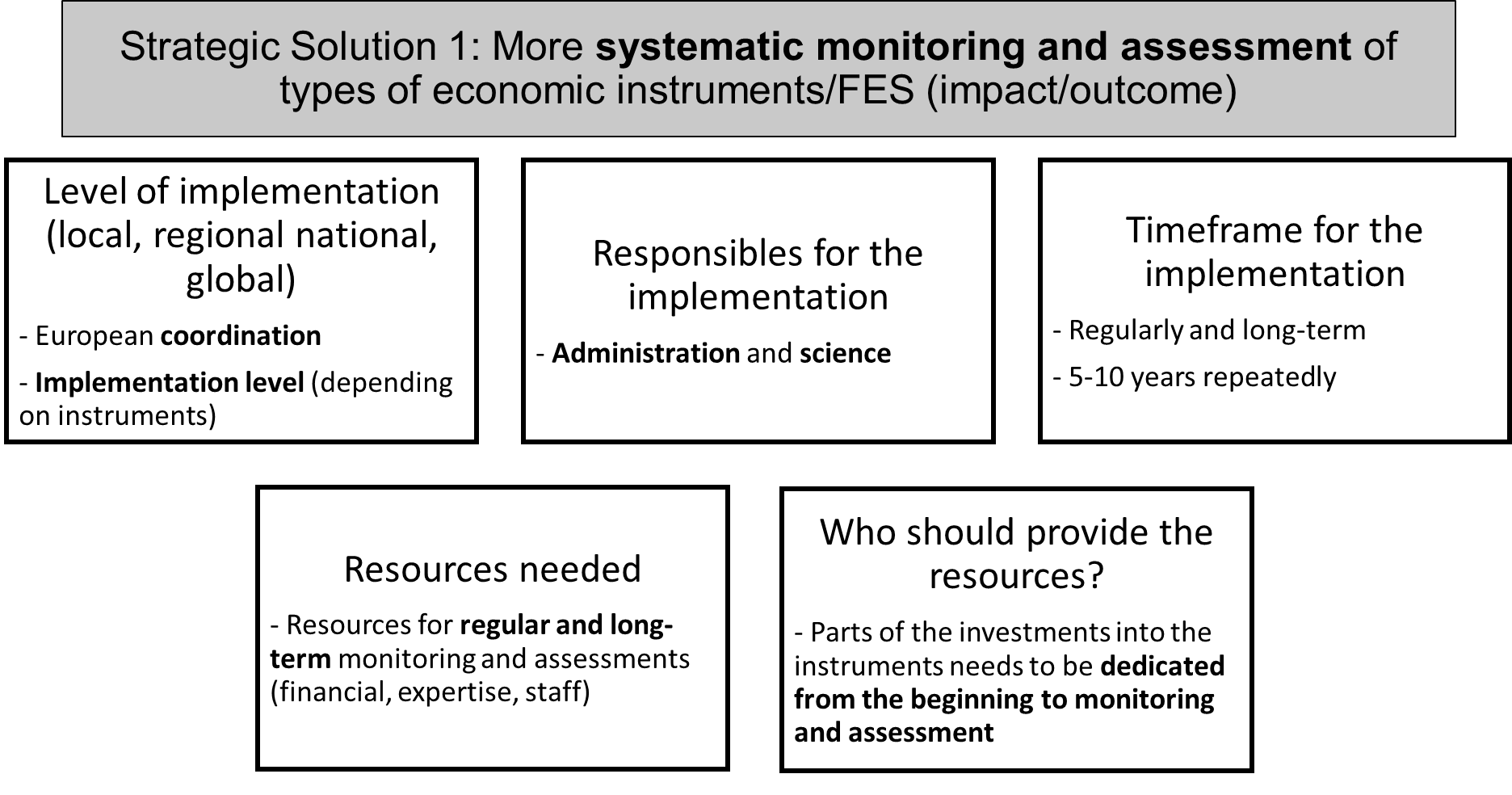

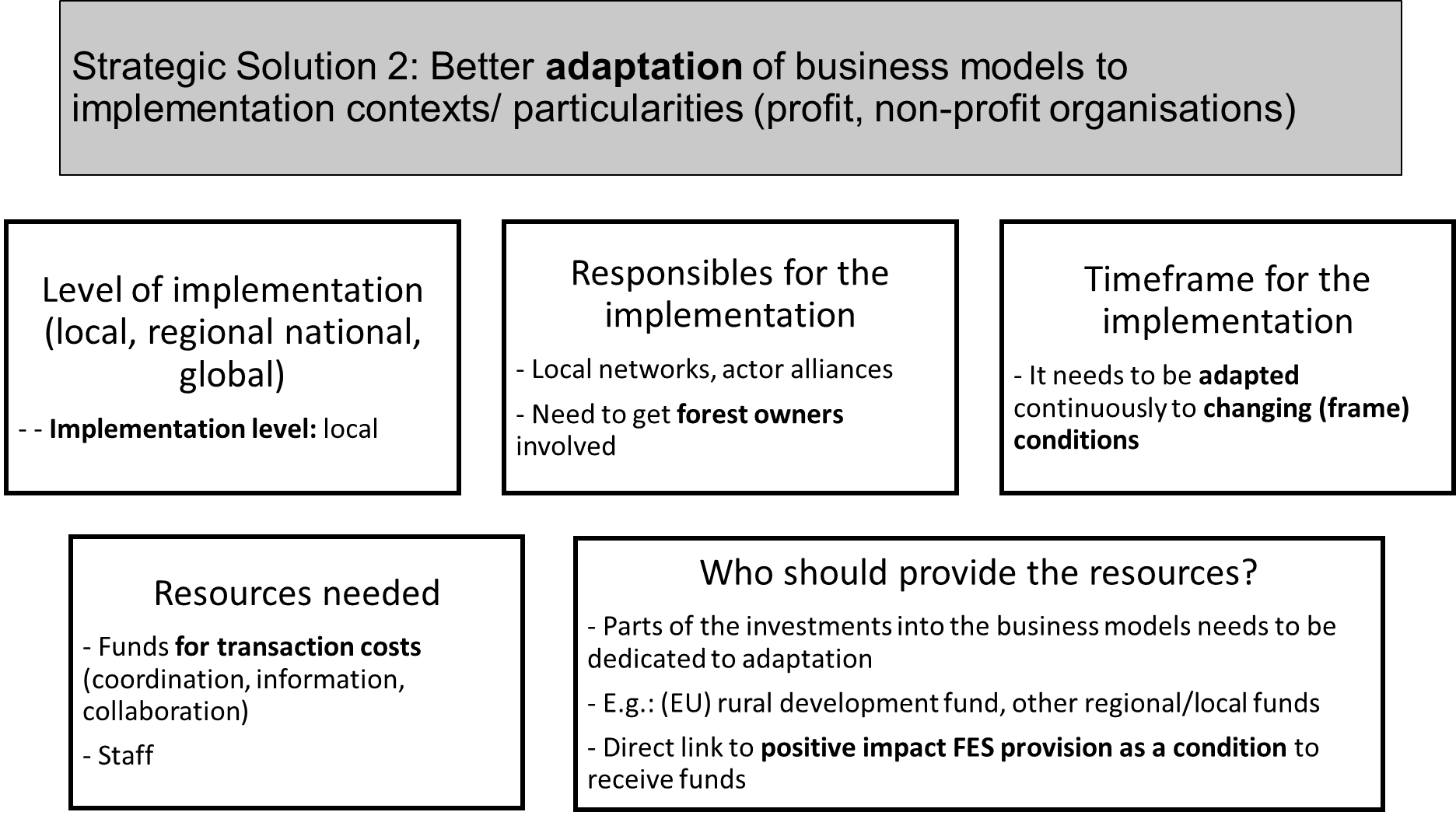


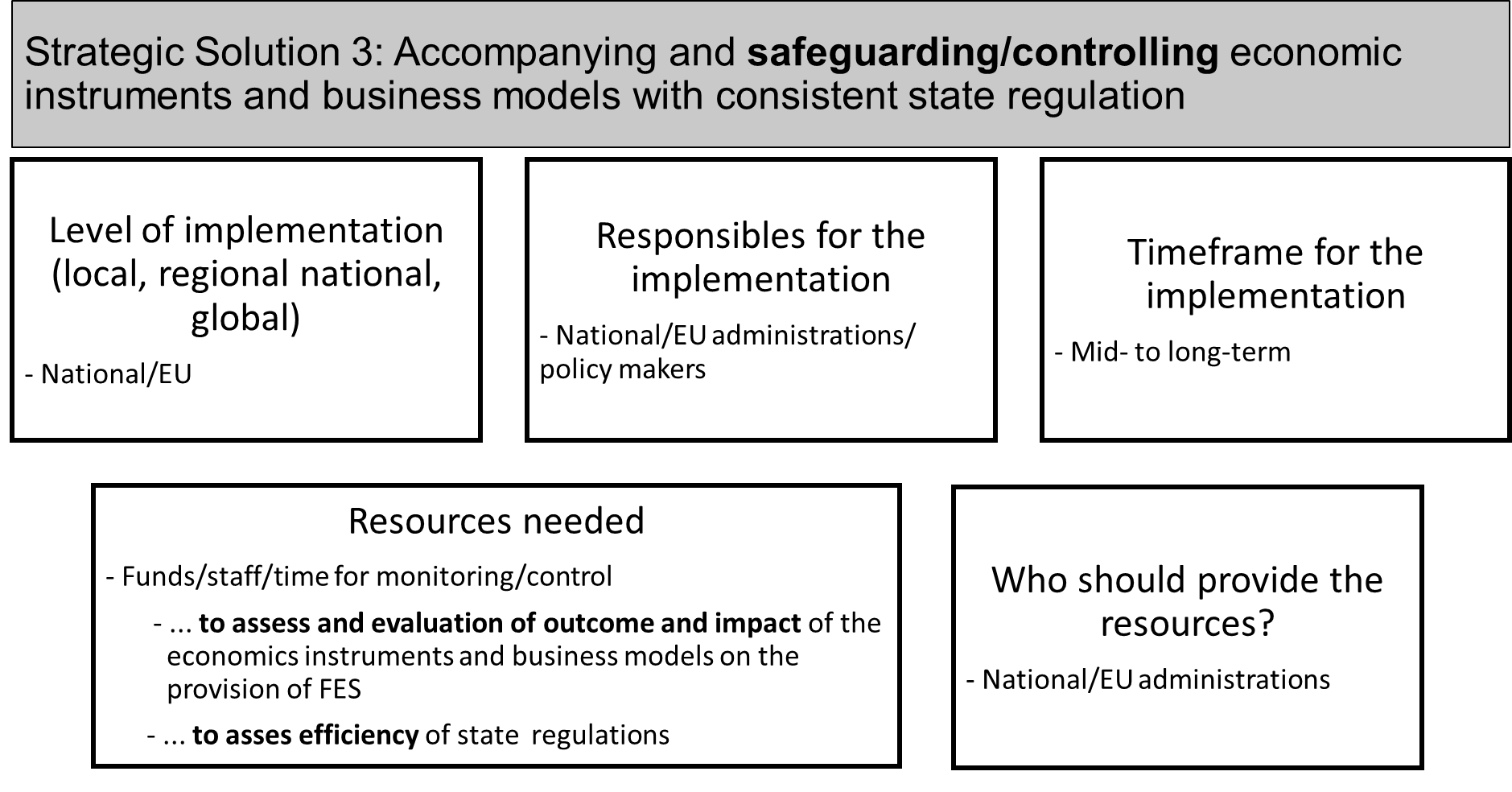


- Challenge 2: Economic power asymmetries in the forest sector dominated and influenced by a reduced number of actors, who control and direct the markets towards intensive biomass and wood/timber production regardless of the negative externalities


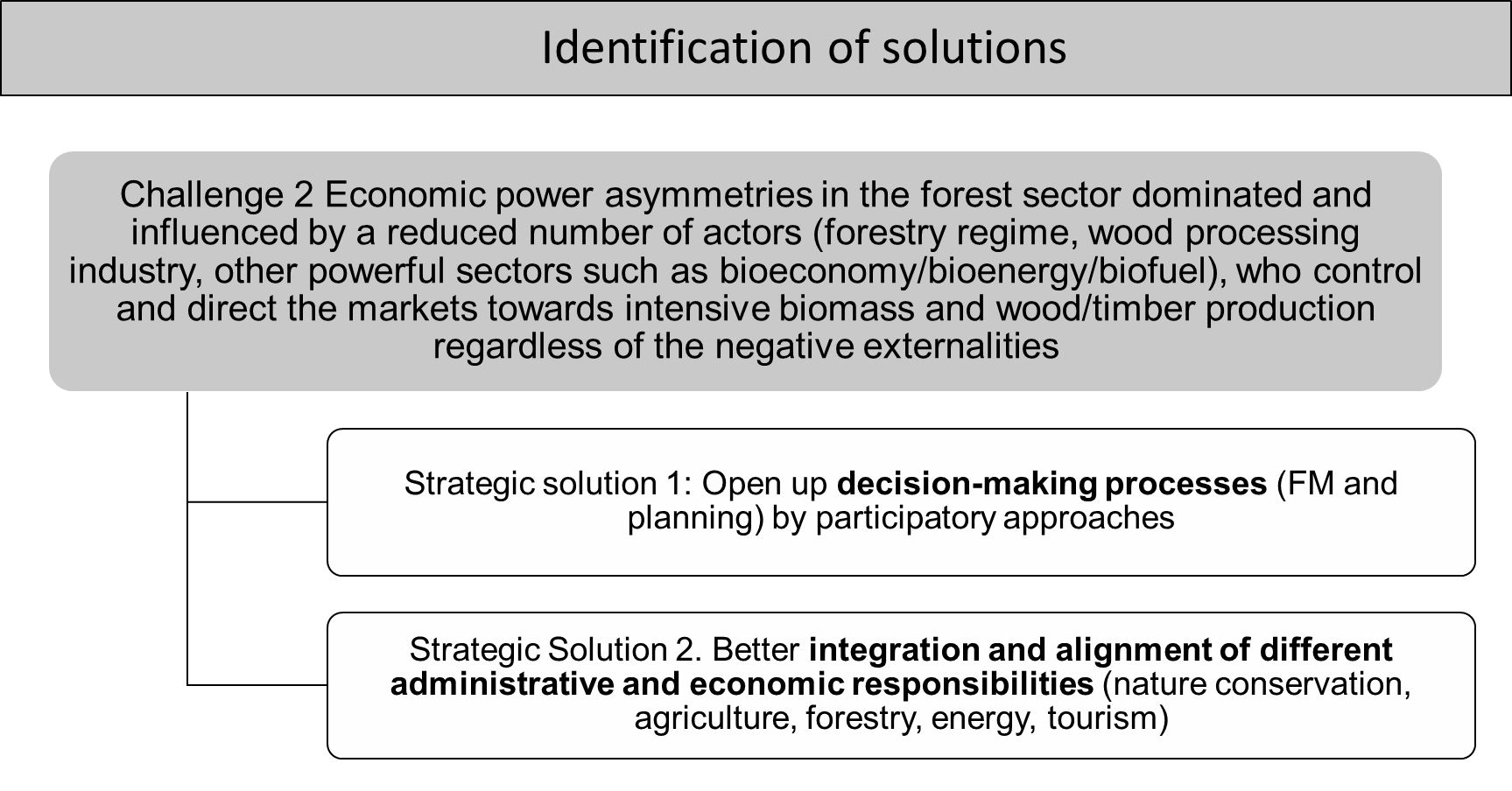


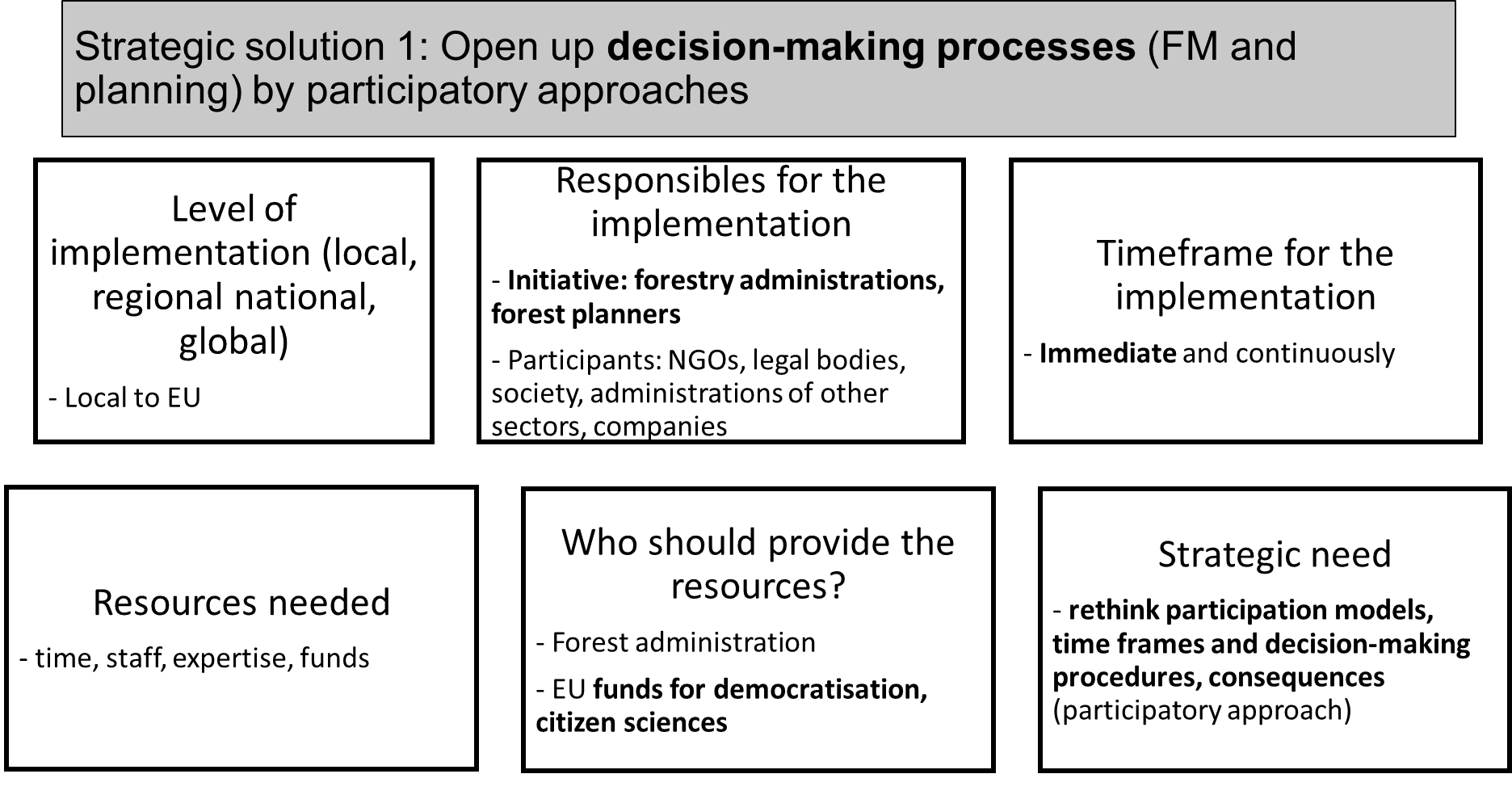


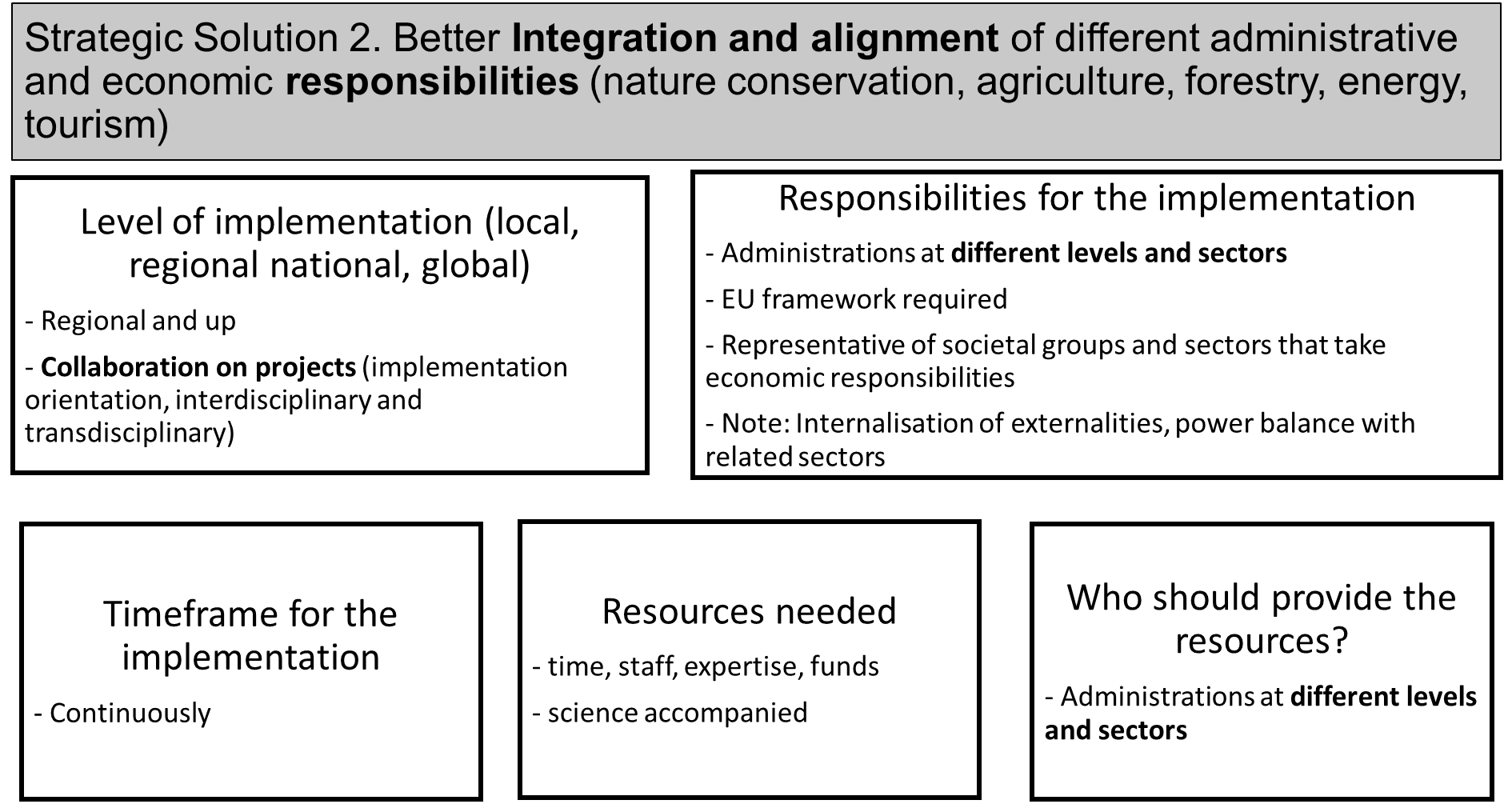


- Challenge 3: Lack/inefficiency of financial tools and support to cover losses from, and adaptation towards natural hazards (e.g. pests, drought, fires, ...extreme weather events, etc.)


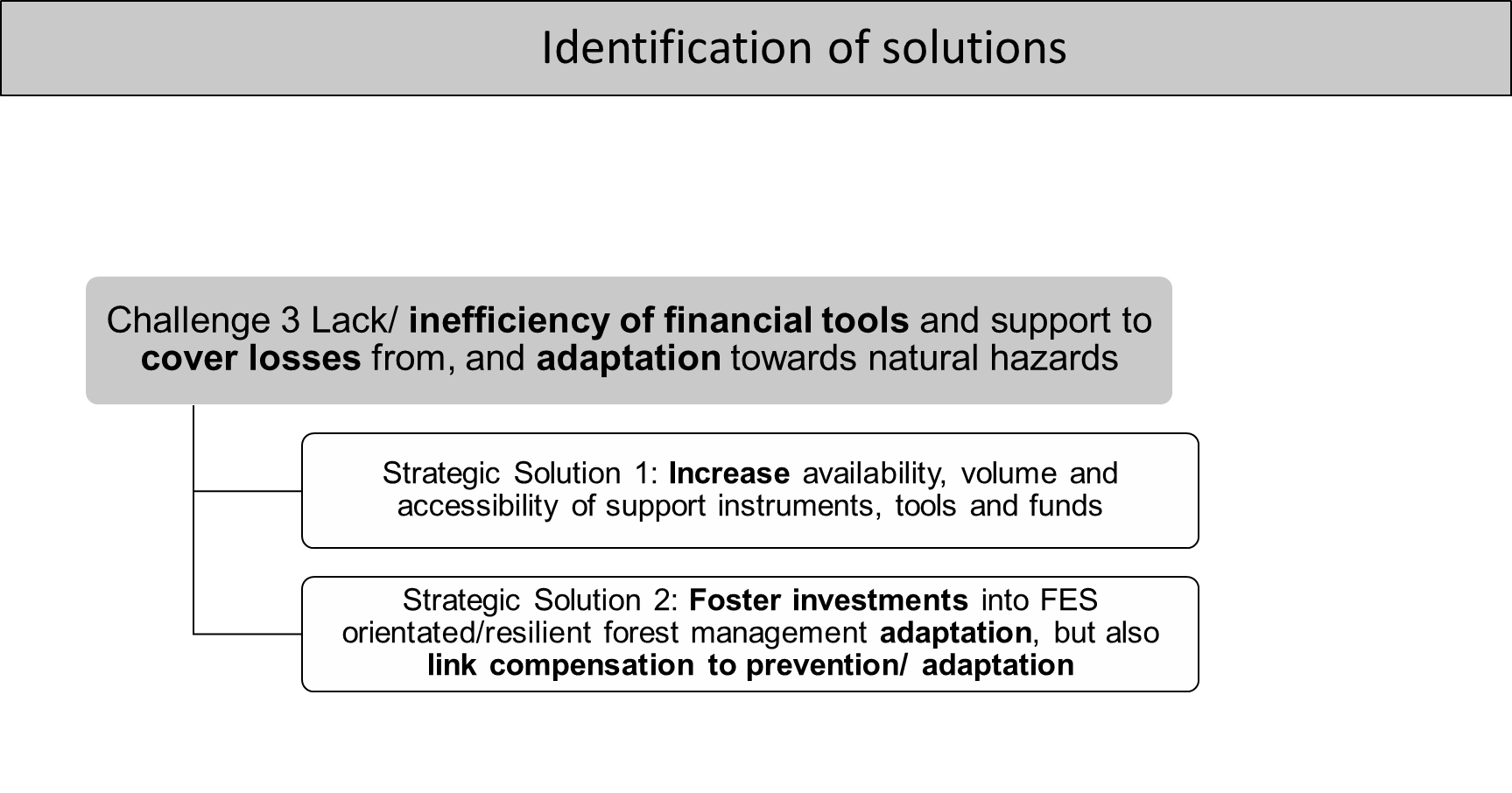

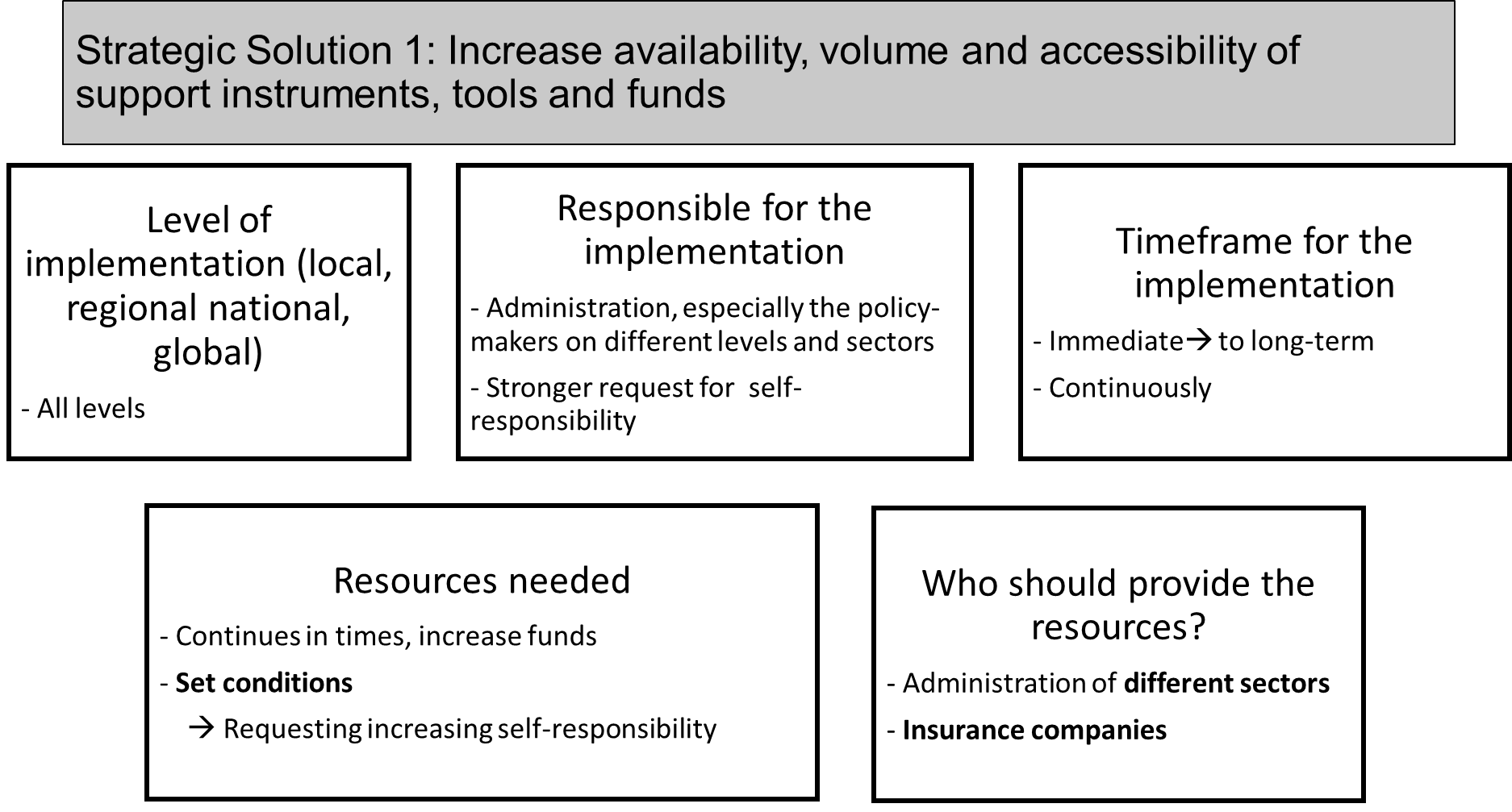

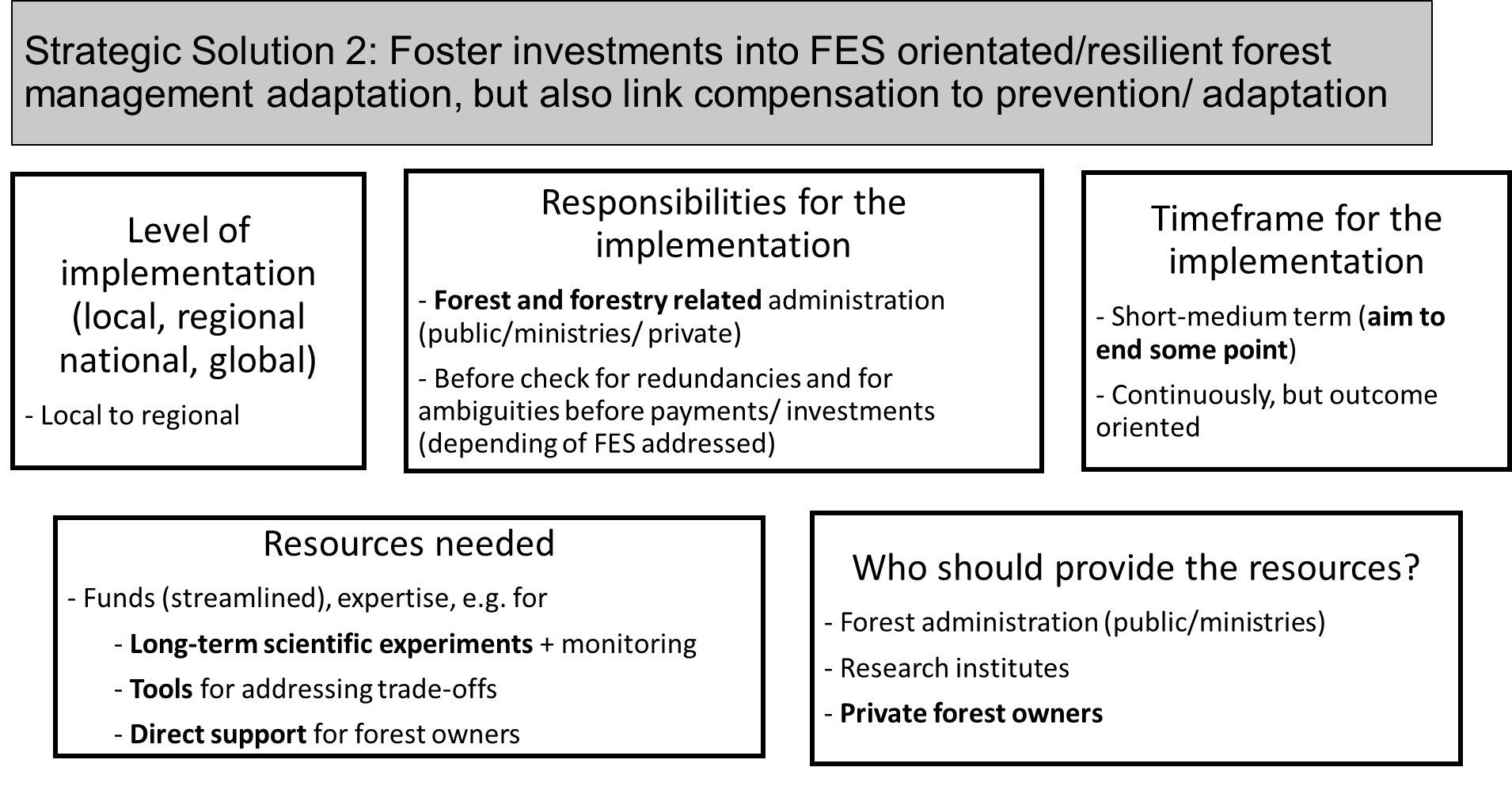


### *Group 4 – MANAGEMENT*

- Challenge 1: Unknown demand and supply of FES for adjust the management strategies accordingly

1. Strategic solution: Observatory at regional level bringing demands and supply from/to society


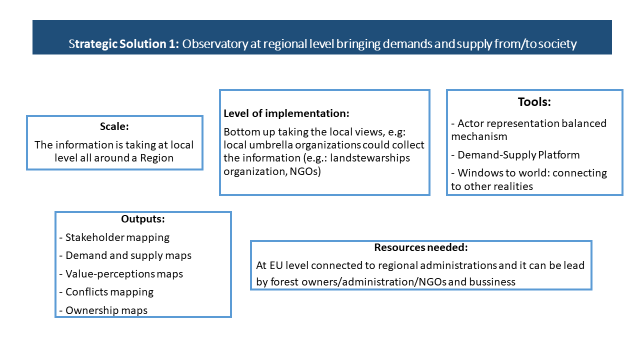


- Challenge 2: Outdated and normative mindset on forest management

1. Strategic solution 1: Mainstreaming the understanding of FES oriented management (raising awareness, education, and networking).


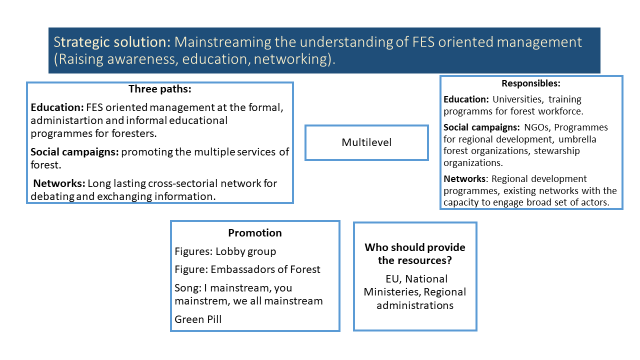


- Challenge 3: Lack of adaptive forest management practices to changing conditions and homogenization of forest stands

1. Strategic solution 1: Development of adaptive strategies to sustain multiple FES based on scenarios.


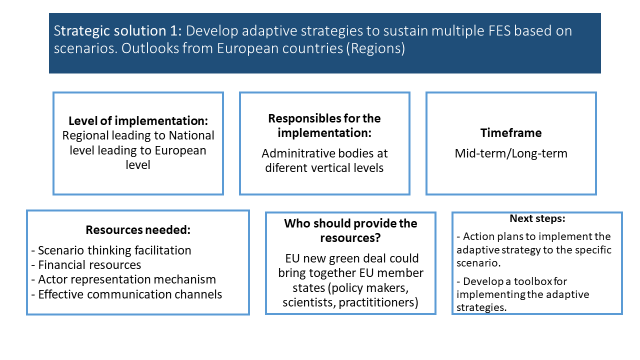


1. Ensuring diversity of forest stands at different levels (genetic, species & forest types)


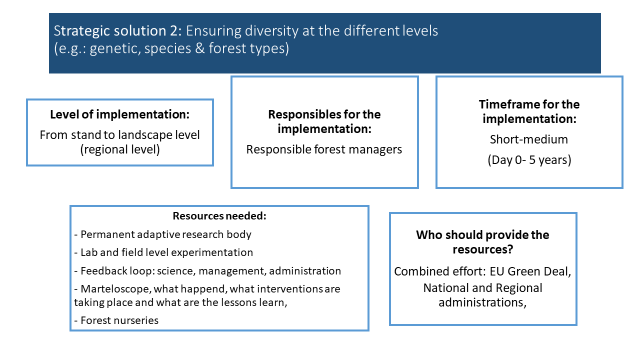


### *Group 5 – GOVERNANCE*

- Challenge 1: Lack of coordination and competition among different policy sectors, especially those with contradicting policy goals and across administrative levels.


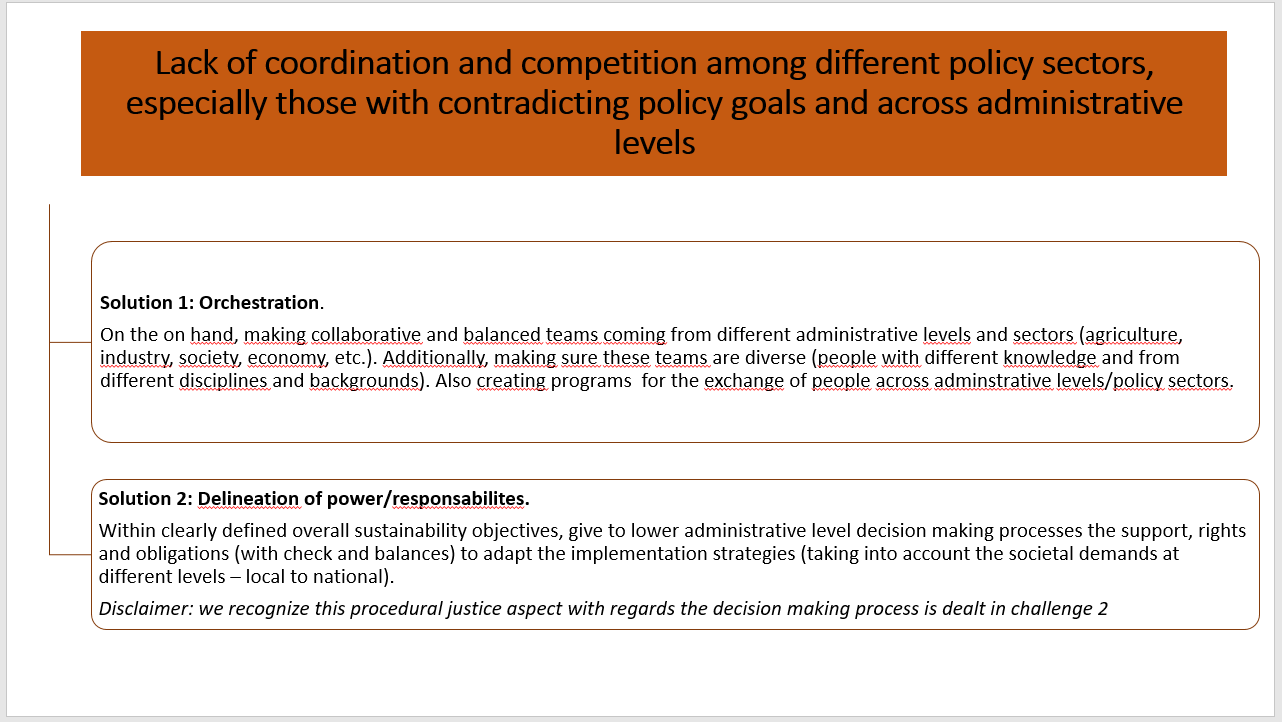


There would be two strategic solutions for this challenge:

1. Orchestration of the adminitration: that could be achieved by the creation of collaborative and balanced teams from differetn administrative levels (to deal with the vertical competition and conflicts) and sectors (to deal with the horizontal competition and conflicts). Also, it often occurs that the problem roots in the fact of the inertia within working groups that have no exchange and end up developing negative attitudes towards other administrations. To solve that, the forestry sector could be inspired by initiatives in other sectors and countries that have programs of exchange of people across departments.
2. Delineation of power/responsabilities: that would mean that lower administrative levels should held more power in making decisions for the implementation of strategies to foster multiple FES. This power would come also with support, rights and oblications. Higher adminsitrative levels would be more responsible on setting the overall objectives and the coordination. We acknowledge that this delineation of power nowadays is implemented in some sectors (i.e. CAP) and does not work very well. That is because in our opinion is not rightly implemented.

- Challenge 2: Lack of engagement and representation in decision making process about the provision and use of FES


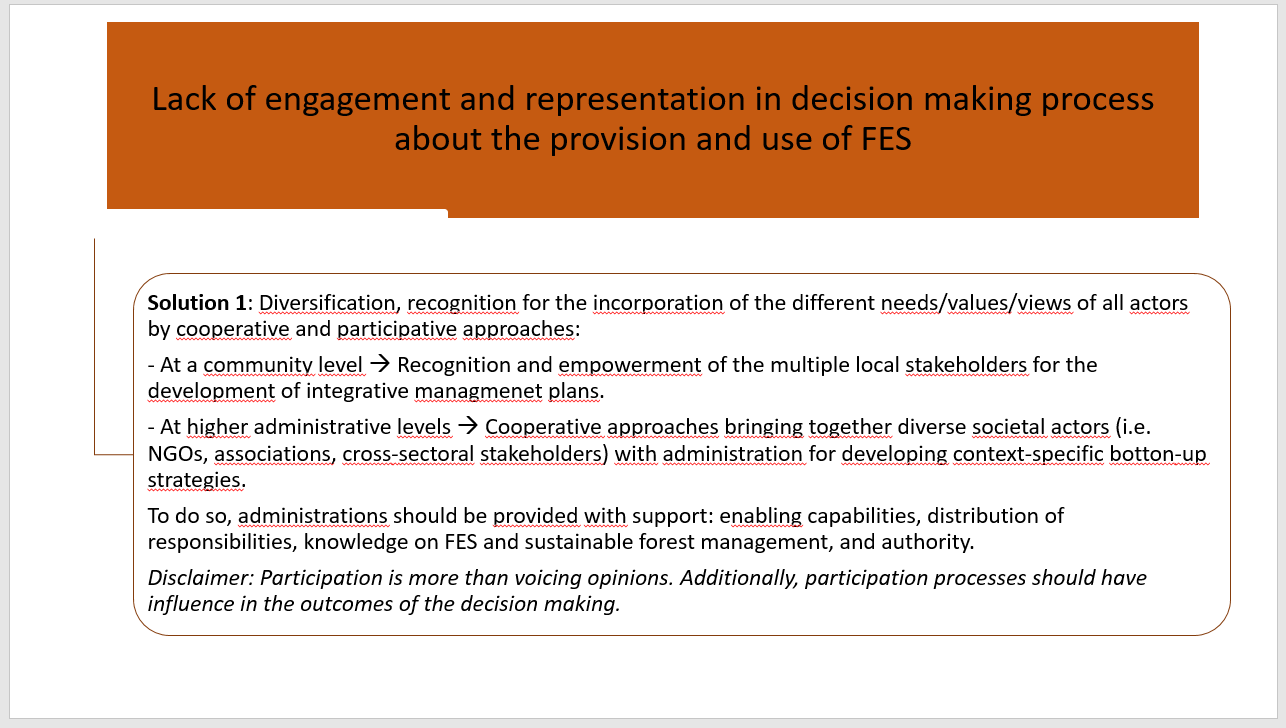


The strategic solution for this challenge would be the generation of spaces for the engagement and representation in decision making processes. This could be achieved through the Diversification, recognition for the incorporation of the different needs/values/views of all actors by cooperative and participative approaches. This should happen at two different levels: community level and higher administrative levels.

It should always be discussed what actually means and imply participation. It should always be more than voicing opinions to incorporate some influence in the decision making.

- Challenge 3: Tensions and mismatching expectations about the role of public forest either as a strategic profitable resource on the one hand and/ or as public goods with the public mandate to provide FES on the other hand


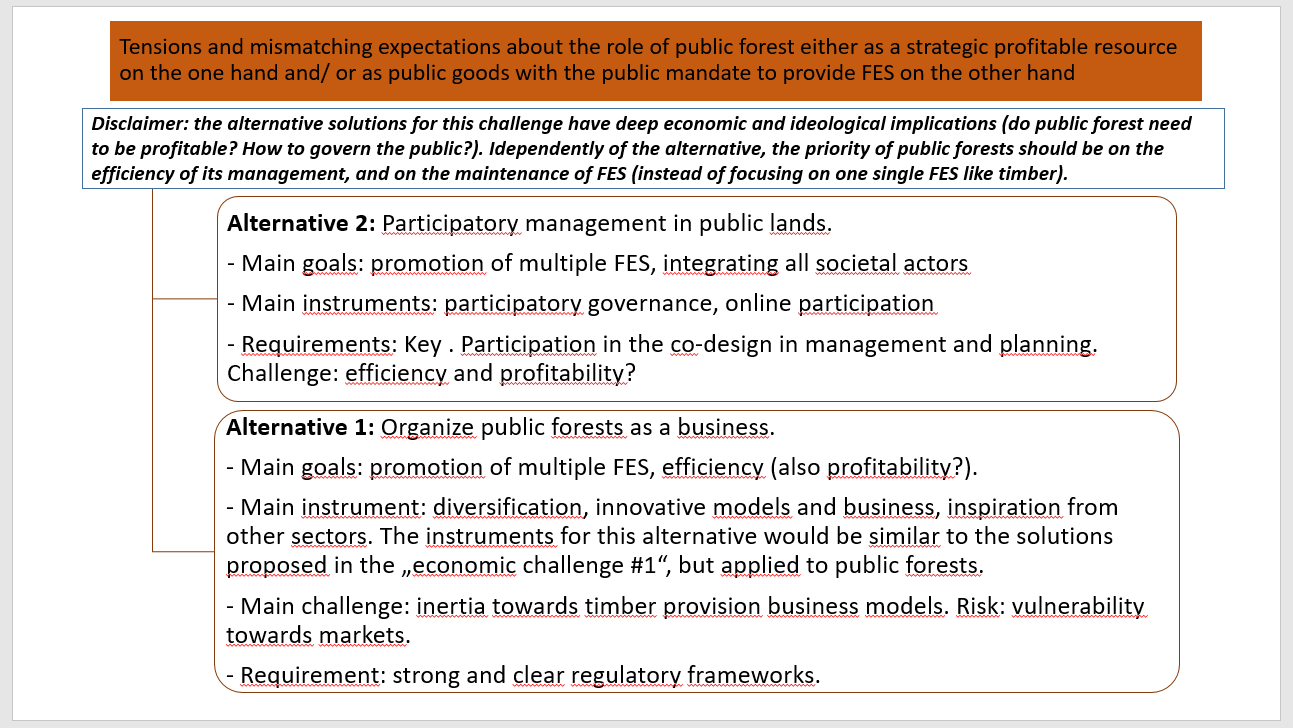


As commented in the previous day, this challenge has an important ideological component. Therefore, more than 2 specific strategic solutions, we find that there are 2 alternatives. Regardless of the alternative, it should be clear that the objective of the management of public forest should be in following the public mandate of fostering multiple FES. However, there would be at least 2 ways to do it:

- Alternative 1: Participatory management in public lands by integrating all societal actors. The instruments for this would be similar to the ones discussed in for the previous challenge. It would require the tools for the public participatory in governance (i.e. online participation).
- Alternative 2: Organize public forests using the principles of business management. In this case, the main goal would be the efficient promotion of multiple FES. The instruments would be diversification, innovative models and business, inspiration from other sectors. However, the challenge would be to avoid the inertia towards timber provision business models. It would require strong and clear regulatory frameworks.

There would be space for a third way, a middle ground where Participatory management is used to overcome management approaches outdated and that do not respond to societal demands or global challenges such as biodiversity loss or climate change (global change) together with a view on embracing innovations towards improved management and efficiency in the use of resources.

Public forests would be used as showcases (lighthouses of innovation) of, for example, public-private partnerships to improve the provision of some FES or enhance NWFP value chains. This should always has an ultimate goal of maximizing the provision of FES, addressing the trade-offs in their provision in a participatory way. These initiatives should be addressed carefully so that public resources do not end up creating exclusively private benefits, but rather improving local economies with a share of benefits re-invested in improved forest management (a sort of virtuous circle).

## **Part VI – Plenary**

In the final session of the workshop, each group presented the identified strategic solutions using the material presented in the previous sections. In the discussion that followed it was made emphasis on the point that most solutions included aspects related to participatory learning.

**S4. Profile of forest and ecosystem services experts**

| **Name** | **Affiliation and email** | **ORCID** | **Area of expertise (what do you do related to forest and FES?)** | **Angle (from which angle do you work? academia, policy and/or practice)** | **Geographical foci (region and country)** |
| --- | --- | --- | --- | --- | --- |
| **Teresa Baiges** | Centre de Propietat Forestal (CPF) tbaiges@gencat.cat |  | Forestry and land use property | Practice | Catalonia, Spain |
| **Andreas Bernasconi** | Pan Bern AG, Postfach, 3001 Bern, Switzerland andreas.bernasconi@panbern.ch | 0000-0002-1913-0361 | Sustainable development and urban forestry | Practice | Switzerland |
| **Giorgia Bottaro** | University of Padova, Land Environment Agriculture and Forestry Department (TeSAF),Viale dell’Università 16, 35020 Legnaro, Padova, Italy. giorgia.bottaro.2@phd.unipd.it | 0000-0002-6744-0962 | Forest, ecosystem services, innovation | Academia | Italy,  Europe,  Chile |
| **Sara Brogaard** | Lund University Centre for Sustainability Studies LUCSUS Lund University Box 170 SE-221 00 Lund, Sweden sara.brogaard@lucsus.lu.se | 0000-0003-0569-0568 | Sustainable land use and ecosystem services | Academia | Sweden |
| **Francesca Bussola** | Forest Service of the Autonomous Province of Trento, via Trener 3, 38121 Trento, Italy bussola.francesca@hotmail.com |  | Support to the forestry local administration in Trento | Practice | Trento, Italy |
| **José V. Roces-Diaz** | Centre for Ecological Research and Forestry Applications (CREAF), Cerdanyola del Valles 08193, Spain j.roces@creaf.uab.cat | 0000-0003-2569-8049 | Assessment and mapping forest ecosystem services | Academia | Spain |
| **Emilio Díaz-Varela** | Research Group COMPASSES-Planning and Management in Social-Ecological Complex Adaptive Systems University of Santiago de Compostela. Campus Universitario, s/n 27002 - Lugo, Spain emilio.diaz@usc.es | 0000-0001-9360-0352 | Ecological planning of landscapes and ecosystems and their services | Academia | Spain |
| **Davide Geneletti** | Department of Civil, Environmental and Mechanical Engineering, University of Trento, via Mesiano 77, 38123 Trento, Italy davide.geneletti@unitn.it | 0000-0002-5528-3365 | Methods for mapping and assessing ecosystem services; ecosystem services trade offs | Academia | Europe, Italy |
| **Carol M. Grossmann** | Forest Research Institute Baden-Wuerttemberg (FVA),  Wonnhaldestrasse 4, 79100 Freiburg, Germany  carol.grossmann@forst.bwl.de | 0000-0001-5914-9844 | Societal change and its influence on forest use and management, ecosystem services, | Academia | Germany, Europe, International |
| **Mónica Hernández-Morcillo** | Eberswalde University for Sustainable Development, Sustainable Forest Resource Economics, Schicklerstrasse 5, 16225 Eberswalde, Germany, mhernandez@hnee.de | 0000-0003-2527-6508 | Forest, ecosystem services and governance innovations | Academia, policy and practice interface | Europe International |
| **Jutta Kister** | University of Innsbruck, Department of Geography, Innrain 52f, 6020 Innsbruck, Austria Jutta.Kister@uibk.ac.at | 0000-0001-9022-9092 | Sustainable economic development, stakeholder participation, Human-environment relations, social innovation | Academia | Austria, Middle Europe, International |
| **Michael Klingler** | University of Natural Resources and Life Sciences Vienna, Institute for Sustainable Economic Development, Feistmantelstraße 4, 1180 Vienna, Austria michael.klingler@boku.ac.at | 0000-0003-0598-9180 | Forest ecosystem services, forest governance, co-production of knowledge | Academia | Europe, Latin America |
| **Lasse Loft** | Working Group Governance of Ecosystem Services, Leibniz Centre for Agricultural Landscape Research (ZALF), Eberswalder Str. 84, 15374 Müncheberg, Germany lasse.loft@zalf.de | 0000-0001-5640-7289 | Policy instruments for the provision of ecosystem services, in particular incentive-based instruments | Academia | Europe, South East Asia, Latin America |
| **Marko Lovrić** | European Forest Institute Yliopistokatu 6B, 80100 Joensuu, Finland, marko.lovric@efi.int | [0000-0002-7729-4743](https://orcid.org/0000-0002-7729-4743) | FES mapping, surveys and upscaling of findings from local to European-level | Academia | Europe, Croatia, Finland |
| **Carsten Mann** | Eberswalde University for Sustainable Development, Sustainable Forest Resource Economics, Schicklerstrasse 5, 16225 Eberswalde, Germany, carsten.mann@hnee.de | 0000-0002-8880-151X | Forest governance, forest ecosystem service, institutional analysis | Academia, policy and practice interface | Europe International |
| **Nathalie Pipart** | KU Leuven, Department of Earth and Environmental Sciences Celestijnenlaan 200E, 3001 Leuven, Belgium nathalie.pipart@kuleuven.be | 0000-0001-8606-7473 | Transdisciplinarity for sustainability, Forest governance | Academia | Europe |
| **Tobias Plieninger** | Faculty of Organic Agricultural Sciences, Universityof Kassel, and Department of Agricultural Economics and Rural Development, University of Göttingen, Platz der Göttinger Sieben 5, 37073 Göttingen, Germany, plieninger@uni-goettingen.de | 0000-0003-1478-2587 | Land use and ecosystem services | Academia | Europe |
| **Stefan Sorge** | Eberswalde University for Sustainable Development, Sustainable Forest Resource Economics, Schicklerstrasse 5, 16225 Eberswalde, Germany, stefansorge@pm.me | 0000-0002-2978-2849 | Forest ecosystem services, innovation science, renewable energies | Academia | Latin America, Africa, Europe |
| **Malin Tiebel** | University of Göttingen, Department of Agricultural Economics and Rural Development, Platz der Göttinger Sieben 5, 37073 Göttingen, Germany malin.tiebel@uni-goettingen.de | 0000-0002-3149-2082 | Small-scale private forest ownership, valuation of ecosystem services, Natura 2000 | Academia | Germany, Europe |
| **Mario Torralba** | Faculty of Organic Agricultural Sciences, Universityof Kassel, Steinstraße 19, 37213, Witzenhausen, Germany mario.torralba@uni-kassel.de | 0000-0001-9205-787X | Valuation of forest ecosystem services | Academina | Europe |
| **Liisa Tyrväinen** | Natural Resources Institute Finland, Latokartanonkaari 9, 00790 Helsinki, Finland liisa.tyrvainen@luke.fi | 0000-0001-5144-7150 | Social and cultural forest ecosystem services, multifunctional forest management | Academia | Finland |
| **Elsa Varela** | Forest Science and Technology Centre of Catalonia, Ctra. St. Llorenç de Morunys, Km2,E-25280 Solsona, Spain elsa.varela@ctfc.es | 0000-0001-9312-6187 | Valuation of forest ecosystem services, | Academia and policy interface | Catalonia, Spain |
| **Georg Winkel** | European Forest Institute (EFI), Platz der Vereinten Nationen; 753113 Bonn, Germany Georg.winkle@efi.int | 0000-0002-9254-0447 | Forest and environmental policy, governance | Academia and policy interface | Europe, Germany |

**S5. Definitions of the identified strategic solutions**

Strategic solutions to environmental challenges

***Solution 1. Promote climate-smart forestry and forest resilience***

Climate-smart forestry is a targeted approach to manage forests in response to climate change (Bowditch et al. 2020). It aims to increase the climate regulation benefits from forests and the forest sector, in a way that creates synergies with other societal needs related to forests. It is a large-scale strategy which unfolds in three main lines of action: the enhancement of natural regeneration and avoidance of deforestation; active forest management; and adaptive forest management to build resilient forests (Nabuurs et al. 2018; Verkerk et al. 2020). For example, a recent analysis along a climate gradient across Europe showed that the higher resilience and resistance to drought events happened in mixed stands compared to monospecific with higher benefits in conifer-broadleaved stands (Pardos et al. 2021). Here, forest resilience considers the maintenance of regimes and the adaptive capacity of the forest as a coupled human-natural system in the face of drivers of change (Nikinmaa et al. 2020). As such, climate-smart forestry strives beyond storing carbon to mitigate climate change, and generate synergies with other FES and biodiversity. The implementation of this solution needs to carefully consider the different regional contexts in Europe to identify the most cost-effective management options. It would also require sustained commitment as the benefits from this solution would only emerge in a mid-long term.

***Solution 2. Improve integration of regulating forest ecosystem services in local and regional planning***

This solution proposes that forest planning authorities consider to a larger extend those specific strategies that have been proven to enhance regulating services such as watershed protection, erosion prevention, or flood control, for example by promoting mixed forest stands of uneven ages (Bravo-Oviedo 2018; Felipe-Lucia et al. 2018). These should be economically supported to cover the opportunity costs needed to restructure forests. Such measures, like PES, already exist in some settings worldwide with different degrees of success (Wunder et al. 2020). The implementation of PES has been polarised between pro-market and anti-neoliberal arguments. To achieve their potential while ensuring an improved environmental governance, a political–cultural reconceptualization should be attained (Van Hecken et al. 2015). Moreover, PES implementation may encounter obstacles hampering the promotion of regulating FES and impeding the improvement of the socioeconomic situation of forest dependent communities and stakeholders. Some of these obstacles are on the social side, the lack of know how, insecure property rights, and problematic benefits distribution, on the market side, the adverse PES self-selection, inadequate administrative targeting, and enforced conditionality (Pagiola et al. 2005; Wunder et al. 2020). There is a large potential for the adaptation of these experiences to the European context.

***Solution 3. Coordinate strategic regional forestry stakeholders to join forces against biological and environmental threats***

This solution proposes a regional-level implementation of coordinated actions and monitoring strategies. Risk can be assessed using analytical techniques that account for threats both spatially and temporally. Subsequently, risk-management strategies need to account more fully for multi level responses that act to balance conflicting interests between stakeholder organizations concerned within the managed and natural environments (Mills et al. 2011). These strategies would integrate private and public forest owners together with the regional-national administration and other sectors depending on the context (e.g., nature conservation, local communities), and backed with national support. The objective would be to share knowledge about affected areas and to join forces for specific forest interventions, increasing the readiness, monitoring capacity, and hence increasing the resilience of the system to these perturbations. An example comes from some regions in the Mediterranean, where civil society engages in wildfires extinction through volunteer groupings (Górriz-Mifsud et al. 2019).

Coordination strategies would need to be specifically adapted to each individual context, as its transferability can be hampered by the heterogeneous systems of management and governance in Europe.

***Solution 4. Implementation of systematic and comprehensive environmental assessments considering multiple scales and cumulative effects of forest fragmentation on FES at landscape level***

Thorough studies on forest fragmentation already exist at a European level (Estreguil et al. 2013; de Montis et al. 2017). However, the effects of fragmentation on the supply of FES have remained largely under-studied (what kind of forest can deliver different ES thresholds in terms of size and quality), while existing knowledge is often scattered and not always backed by empirical data. Different FES operate at different scales and are impacted differently by fragmentation (Mitchell et al. 2015). In this context, this solution proposes the systematic incorporation of fragmentation and connectivity of FES into environmental assessments considering multiple scales and cumulative effects of forest fragmentation. This process would allow the discrimination between those areas that are still large enough to provide FES, to those that need to be (re-)extended or interconnected to ensure the provision of different FES. While this solution would be relevant for allowing public administrations to undertake science and monitoring based management decisions, the theoretical and methodological requirements for the practical integration of multiple FES is still in development (Bennett 2017).

Strategic solutions to management challenges

***Solution 5. Mainstream FES-oriented management in a threefold strategy: education, awareness raising, and networking***

This solution suggests broadening the often narrow perspective of forest management focused on timber and biomass production of highly productive stands (Jönsson and Snäll 2020) with help of education and information strategies. In particular, this could be done by diversifying education at the administration and university level (Nair 2004), fostering knowledge transfer to forest operators (Perera et al. 2006), starting and reinforcing social campaigns to make visible the multiple services of forest, and developing and enabling long lasting cross-sectorial networks (Guerrero and Hansen 2021). Although this solution requires long-term commitment and significant attitudinal changes within and beyond the forestry sector (shifting management goals, seeking long term instead of short-term benefits, or changing contractual arrangements) before its effects become apparent, this solution has the potential to largely generate synergistic and long-lasting effects over forest management in Europe. To tackle complex challenges and developing opportunities for innovation at EU level, collaboration can be enhanced through existing European Innovations Partnership (EIP) operational groups on forest and EU projects through multi-actor approaches such as InnoForESt and SINCERE. Moreover, in the light of the new EU CAP, Agricultural Knowledge and Innovation Systems (AKIS) are key to support the share of knowledge and innovative applications more intensively.

***Solution 6: Develop adaptive strategies to sustain multiple FES based on regional scenarios***

This solution consists of a series of regional multi-stakeholder’s workshops to, based on existing information (e.g., climate, tree species distribution), develop regional scenarios (Oteros-Rozas et al. 2015) and the identification of the most effective adaptive strategies and a toolbox for their implementation to provide sustainable FES in the EU. While this proposed process conducted at a strategic regional level would be a good first step before implementation, the quality of the scenarios would be largely dependent on the available resources and the previously established networks. In Europe, many projects have directly or indirectly advanced towards this objective (e.g., SINCERE and InnoForESt). A step towards this solution would be the capitalization and local-proof validation of this work.

**Solution 7: Ensure diversity at different levels (genetic, species, and forest)**

This solution strategy consists of a multi-step approach starting at national level, investigating the potential for different types and species diversity capacity at different forest stands within the country regions. To do this, the EU New Green Deal together with regional and local administration could allocate part of the budget to implement the most effective diversity strategy that would ensure the most sustainable FES provision.

***Solution 8. Establish regional observatories for capturing societal FES demand and supply***

This solution involves the creation of a network of centers that gather the information on FES demand and supply at local level across all European regions. The level of implementation is bottom-up considering local views. The task could be taken up by establishing local umbrella organizations responsible for collecting the information. This solution would require extensive preliminary work to define a monitoring framework and to develop clear and concrete indicators for capturing regional FES supply and demand (Maes et al. 2012; Wolff et al. 2015). If efficiently implemented, these observatories would constitute a reference for monitoring and a source of ideas for transferability.

Strategic solutions to economic challenges

***Solution 9. Foster investments into FES oriented forest management to increase resilience (prevention and adaptation) towards natural hazards***

Investing in increasing forest resilience (Nikinmaa et al. 2020) is key for ensuring the prevention and adaptation towards natural hazards and ensuring the sustainable provision of FES (Keenan 2015; Lecina‐Diaz et al. 2021). After assessing the redundancies and ambiguities of forest-related investments, local to regional forestry together with nature conservation administrations levels should be in charge of articulating and administering these investments. This should be implemented and monitored in a short-medium term to ensure that they foster sustainable solutions with regard to multiple forest functions. This support needs to be continuous and outcome-oriented by designing policies that consider spatial targeting to FES density, threats and cost levels, payment differentiation, and improved conditionality (Wunder et al. 2020). This solution requires an integrated forest policy that addresses various system dimensions in terms of policy sectors and administrative levels, including both local and landscape-level land uses with indicators oriented towards minimizing socio-ecological damages and losses (Moreira et al. 2020).

***Solution 10. Increasing availability, volume, and accessibility of supporting financial instruments to cover losses from natural hazards***

The current natural hazards require planning and management strategies that increase forest capacity for adaptive transformation. It could provide an opportunity to steer the objectives of forest management towards a more sustainable and less production-oriented model. To be efficient, financial instruments need to be conditional upon sustainable practices that ensure a diverse FES provision, while being adapted to the different realities existing in the European forestry sector. This could be achieved by dedicating part of the existing economic support (e.g., EU rural development fund, common agricultural policy, other regional/local funds) for business model implementation to its adaptation to each specific context, refocusing for example forest protection measures (Alliance Environnement EEIG 2017) and encouraging the use of result-based schemes in order to potentially increase the impact of the funding, while linking the business model with a positive and measurable impact on the FES provision (ECA 2020). Within this scheme, a requirement for eligibility to receive funds would be the direct link between the business model and a positive impact FES provision (Wunder et al. 2018; Ovando et al. 2019).

***Solution 11: Support economic instruments and business models promoting regulating and cultural FES with consistent policies***

Effective economic instruments as well as business models that contribute to the sustainable provision of FES (particularly for regulating and cultural FES) should be consistently supported by cross-scale European and national policies similar to those applied for timber and biomass production (Wunder et al. 2019). This could be achieved through, on the one hand, nested multi-scale policies (Ostrom 1990) and, on the other hand, a strategy of making available and advertising successful business models along with the key features leading to their success to allow replication elsewhere. In relation to incentive-based and result-based PES payment schemes, it is important to target forest owners of those forest areas that show a) high levels of FES supply (e.g., high carbon stocks/ha or endemic biodiversity hotspots), and b) areas with high potential risks (e.g., high threat of deforestation and degradation). This strategy would focus PES in areas where they can realistically make a difference (Börner et al. 2020; Wunder et al. 2020).

***Solution 12. Align finance and administration of different sectors***

This solution proposes the line-up of different administrative and economic responsibilities by adjusting and delimiting budgets and objectives pertaining to different sectors such as nature conservation, agriculture, forestry, energy, and tourism (Börner et al. 2020).

***Solution 13: Improve adaptation of business models to particular contexts of implementation***

To be efficient, business models for the sustainable use of FES need to be adapted to different realities. Therefore, this solution proposes to dedicate part of existing economic support (EU rural development fund, other regional/local funds) for business model implementation to its adaptation to each specific context. Within this scheme, a requirement for eligibility to receive funds would be the direct link between the business model and a positive impact FES provision (Wunder et al. 2018)*.*

***Solution 14: Monitor systematically the socio-ecological impact of economic instruments***

Periodic and tailored assessments of particular economic instruments for individual/groups of FES types with attention paid to its social-ecological impacts and outcomes (Fischer 2018). Alone, this solution would not solve any challenge, but would lay important groundwork towards developing potential pathways towards solving them.

Strategic solutions to governance challenges

***Solution 15. Promote vertical and horizontal coherence in administration***

For a long time, orchestration has been identified as a potential effective solution for many environmental challenges within the forest sector, despite its challenging implementation (Kleinschmit et al. 2018; Secco et al. 2018). This solution would entail on the on hand, the creation of collaborative and balanced teams coming from different administrative levels (vertical orchestration: local, regional, etc.) and sectors (horizontal orchestration: agriculture, industry, society, economy, etc.). These teams would be diverse and incorporate members with different knowledge and from different disciplines and backgrounds. On the other hand, temporal exchange programs for people across administrative levels/policy sectors would be created to create links, promote cross-fertilization, and avoid the creation of avoidable pernicious and insulating inertias within administrative departments.

***Solution 16. Delineate clear and stable power structures structure and responsibilities***

This solution proposes that clear overall sustainability objectives should be defined at high administrative levels, while lower administrative levels would hold more power in decision making processes for the implementation of strategies to foster multiple FES. This power would be accompanied with support, rights, and obligations (with checks and balances) in the adaptation and implementation processes (considering the societal demands at different levels – local to national).

***Solution 17. Generate spaces for stakeholders’ engagement and representation in decision making processes in cooperative and participative approaches***

This solution involves the generation of spaces for stakeholder’s engagement and representation in decision making process with command authority, through the diversification, recognition, and incorporation of the different needs, values, and views of all actors by cooperative and participative approaches. Participation should be more than voicing opinions. Additionally, participation processes should have influence in the outcomes of the decision making (Cornwall 2008; Reed 2008). This would be operationalized at two levels:

- At a community level: recognition and empowerment of the multiple local stakeholders for the development of integrative management plans (Luyet et al. 2012; Kraus and Krumm 2013).
- At higher administrative levels: cooperative approaches bringing together diverse societal actors (i.e., NGOs, associations, cross-sectoral stakeholders) with administration for developing context-specific bottom-up strategies. To do so, administrations should be provided with specific support: enabling capabilities, distribution of responsibilities, knowledge-transfer on FES and sustainable forest management, and crucially, increased command authority.

***Solution 18. Engage the community in participatory decision-making approaches for the management of public forests, while embracing innovations towards efficient use of forest resources***

This solution strategy promotes participatory forest management to overcome outdated management approaches that do not respond to current societal demands and larger social-ecological challenges (such as biodiversity loss or climate change). These strategies are often coupled with a philosophy of embracing innovations towards improved forest management for the provision of FES bundles, specially for regulating and cultural FES, for the promotion of ecological and societal transformation, and for the sustainable use of public goods. Public forests would be used as niches of innovation (Geels 2005) of, for example, public-private partnerships or novel actor alliances to improve the provision of regulating and cultural FES or enhance non-wood forest products (NWFP) value chains. Public forests would act as ‘incubation rooms’ for radical novelties, providing locations for learning processes, and spaces to build the social networks which support innovation. Initiatives and innovations would be carefully addressed so that public resources do not end up creating exclusively private benefits, but rather improving local economies with a share of benefits re-invested in improved forest management.

***Solution 19. Integrate all actors in participatory decision making about management goals of public forest lands***

Participatory management to overcome outdated management approaches that do not respond to current societal demands and larger social-ecological challenges (such as biodiversity loss or climate change), coupled with a philosophy of embracing innovations towards improved management and efficiency in the use of resources. Public forests would be used as showcases (lighthouses of innovation) of, for example, public-private partnerships to improve the provision of some FES or enhance NWFP value chains. The ultimate goal would be the provision of multiple FES, addressing the trade-offs in their provision in a participatory way. Initiatives and innovations would be carefully addressed so that public resources do not end up creating exclusively private benefits, but rather improving local economies with a share of benefits re-invested in improved forest management.

***Solution 20. Streamline public forest management organization and administration following the principles of the private forest sector***

The main goal of management is the efficient promotion of multiple FES. The instruments to achieve this goal would be the diversification and incorporation of innovative models and businesses inspired by other sectors. This alternative solution would require strong and clear regulatory frameworks to avoid the inertia towards timber provision business models. This solution might be controversial, as currently many European public forests are already managed following the principles and modes of private forest land without achieving the sustainable promotion of FES. While the general model would stay in place, the management objectives of those public forests would need to be shifted and re-aligned to match the public mandate for the efficient promotion of multiple FES.

Strategic solutions to socio-cultural challenges

***Solution 21. Implement practices for (re)connecting people with forests***

Understanding forests as a mean to solve economic problems is a reducionistic standpoint. In the pursuit of sustainable forest management, the increased identification and inclusion of cultural bonds is crucial. To achieve a deeper understanding of the mutual constitution of society-forest relations, it is also necessary to recognize the multi-layered spectrum of forests’ contributions (Ritter and Dauksta 2013). Mainstreaming forest models that (re)connect people and forests (like forest kindergartens and forest schools) is crucial. Increasing studies show the perceived linkages of people to spiritual and cultural values in forest that are not necessarily related to livelihoods (Rodríguez-Morales et al. 2020; Torralba et al. 2020). In parallel, there is a need to strengthen the social and cultural sciences in FES assessments with a clearer representation of non-material values (Jacobs et al. 2016) and more-than-human thinking (Whatmore 2006).

***Solution 22. Strengthen the recognition, identification, and integration of social-cultural values in forest management, governance, and research***

Strengthening of the use of participatory tools at several steps (stakeholder analysis, assessments, planning strategies, governance platforms, and participatory management models) focused on the recognition, identification, and integration of social-cultural values (Berkes 2009; Scholte et al. 2015; Jacobs et al. 2016). This solution would involve the progressive integration of local communities in forest planning at local scales, while larger-scale policies would ensure the integration and recognition of those marginalized parts of society that are less recognized. In the academic arena, it would mean the inclusion of forest knowledge and traditions from outside Europe.

***Solution 23. Promote new forms of communication and interaction between society and FES providers with a focus on public forests***

When forests provide more regulating or cultural services than provisioning services, governance mechanisms are key to maintain the supply of FES, especially in private owned forests. To overcome the lack of markets to deal with public goods and services, social support is needed to finance the expenses which keep the sustainable forest management ongoing; especially in situations where management is key to guarantee the provision flow of these goods and services, but these are under high threat (e.g., wildfire risk in the Mediterranean region that increases with lack of active forest management). European studies of public perception (Rametsteiner et al. 2009) reveal that forestry issues are not well understood outside the forestry community and suggest that improving communication to the general public is essential. Management goals and objectives must be identified and communicated at short as well as long term, a wide variety of channels should be used, messages should be simple and clear, and collaboration with other organizations (agriculture, wood construction, etc.) should be enhanced. The joint effort with media professionals would turn into more successful results. In parallel, further research into public perception of forests and forestry is needed to define targeted communication strategies (Fabra-Crespo and Rojas-Briales 2015).

***Solution 24. Build capacities as a tool to prevent abandonment and promote generational turnover in the forest sector***

The final solution involves the integration of tradition and innovative knowledge, promotion of social innovation, and creation of cross-sectoral networks. Integration and capacity building is in many European contexts a requisite to activate citizens into participation in forest planning processes. The positive effects of this solution would only emerge in the medium/long term period of time, but its effect would be lasting.

**References**

- Alliance Environment EEIG (2017) Evaluation study of the forestry measures under Rural Development. European Commission
- Bennett EM (2017) Research Frontiers in Ecosystem Service Science. Ecosystems 20:31–37. doi: 10.1007/s10021-016-0049-0
- Berkes F (2009) Evolution of co-management: Role of knowledge generation, bridging organizations and social learning. J. Environ. Manage. 90:1692–1702
- Börner J, Schulz D, Wunder S, Pfaff A (2020) The Effectiveness of Forest Conservation Policies and Programs. Annu Rev Resour Econ 12:45–64. doi: 10.1146/annurev-resource-110119-025703
- Bowditch E, Santopuoli G, Binder F, et al (2020) What is Climate-Smart Forestry? A definition from a multinational collaborative process focused on mountain regions of Europe. Ecosyst Serv 43:101113. doi: 10.1016/j.ecoser.2020.101113
- Bravo-Oviedo A (2018) The Role of Mixed Forests in a Changing Social-Ecological World. In: Dynamics, Silviculture and Management of Mixed Forests. Managing Forest Ecosystems. Springer, Cham, pp 1–25
- Cornwall A (2008) Unpacking “Participation” Models, meanings and practices. Community Dev J 43:269–283. doi: 10.1093/cdj/bsn010
- de Montis A, Martín B, Ortega E, et al (2017) Landscape fragmentation in Mediterranean Europe: A comparative approach. Land use policy 64:83–94. doi: 10.1016/j.landusepol.2017.02.028
- ECA (2020) Special Report Biodiversity on farmland: CAP contribution has not halted the decline
- Estreguil C, Caudullo G, De Rigo D, San Miguel J (2013) Forest Landscape in Europe: Pattern, Fragmentation and Connectivity. JRC Scientific and Policy Report EUR 25717EN
- Fabra-Crespo M, Rojas-Briales E (2015) Comparative analysis on the communication strategies of the forest owners’ associations in Europe. For Policy Econ 50:20–30. doi: 10.1016/j.forpol.2014.06.004
- Felipe-Lucia MR, Soliveres S, Penone C, et al (2018) Multiple forest attributes underpin the supply of multiple ecosystem services. Nat Commun 9:1–11. doi: 10.1038/s41467-018-07082-4
- Fischer AP (2018) Forest landscapes as social-ecological systems and implications for management. Landsc Urban Plan 177:138–147. doi: 10.1016/j.landurbplan.2018.05.001
- Geels FW (2005) Processes and patterns in transitions and system innovations: Refining the co-evolutionary multi-level perspective. Technol Forecast Soc Change 72:681–696. doi: 10.1016/j.techfore.2004.08.014
- Górriz-Mifsud E, Burns M, Marini Govigli V (2019) Civil society engaged in wildfires: Mediterranean forest fire volunteer groupings. For Policy Econ 102:119–129. doi: 10.1016/j.forpol.2019.03.007
- Guerrero JE, Hansen E (2021) Company-level cross-sector collaborations in transition to the bioeconomy: A multi-case study. For Policy Econ 123:102355. doi: 10.1016/j.forpol.2020.102355
- Jacobs S, Dendoncker N, Martín-López B, et al (2016) A new valuation school: Integrating diverse values of nature in resource and land use decisions. Ecosyst Serv 22:213–220. doi: 10.1016/J.ECOSER.2016.11.007
- Jönsson M, Snäll T (2020) Ecosystem service multifunctionality of low‐productivity forests and implications for conservation and management. J Appl Ecol 57:695–706. doi: 10.1111/1365-2664.13569
- Keenan RJ (2015) Climate change impacts and adaptation in forest management: a review. Ann For Sci 72:145–167. doi: 10.1007/s13595-014-0446-5
- Kleinschmit D, Pülzl H, Secco L, et al (2018) Orchestration in political processes: Involvement of experts, citizens, and participatory professionals in forest policy making. For Policy Econ 89:4–15. doi: 10.1016/j.forpol.2017.12.011
- Kraus D, Krumm F (2013) Integrative approaches as an opportunity for the conservation of forest biodiversity. European Forest Institute
- Lecina‐Diaz J, Martínez‐Vilalta J, Alvarez A, et al (2021) Characterizing forest vulnerability and risk to climate‐change hazards. Front Ecol Environ 19:126–133. doi: 10.1002/fee.2278
- Luyet V, Schlaepfer R, Parlange MB, Buttler A (2012) A framework to implement Stakeholder participation in environmental projects. J. Environ. Manage. 111:213–219
- Maes J, Egoh B, Willemen L, et al (2012) Mapping ecosystem services for policy support and decision making in the European Union. Ecosyst Serv 1:31–39. doi: 10.1016/j.ecoser.2012.06.004
- Mills P, Dehnen-Schmutz K, Ilbery B, et al (2011) Integrating natural and social science perspectives on plant disease risk, management and policy formulation. Philos Trans R Soc B 366:2035–2044. doi: 10.1098/rstb.2010.0411
- Mitchell MGE, Suarez-Castro AF, Martinez-Harms M, et al (2015) Reframing landscape fragmentation’s effects on ecosystem services. Trends Ecol Evol 30:190–198. doi: 10.1016/j.tree.2015.01.011
- Moreira F, Ascoli D, Safford H, et al (2020) Wildfire management in Mediterranean-type regions: Paradigm change needed. Environ. Res. Lett. 15:011001
- Nabuurs G-J, Verkerk PJ, Schelhaas M-J, et al (2018) Climate-Smart Forestry: mitigation impacts in three European regions. European Forest Institute
- Nair CTS (2004) What does the future hold for forestry education? Unasylva 55: 216:
- Nikinmaa L, Lindner M, Cantarello E, et al (2020) Reviewing the Use of Resilience Concepts in Forest Sciences. Curr For Reports 6:61–80. doi: 10.1007/s40725-020-00110-x
- Ostrom E (1990) Governing the Commons. Cambridge University Press, Cambridge
- Oteros-Rozas E, Martín-López B, Daw TM, et al (2015) Participatory scenario planning in place-based social-ecological research: Insights and experiences from 23 case studies. Ecol Soc 20:. doi: 10.5751/ES-07985-200432
- Ovando P, Beguería S, Campos P (2019) Carbon sequestration or water yield? The effect of payments for ecosystem services on forest management decisions in Mediterranean forests. Water Resour Econ 28:100119. doi: 10.1016/j.wre.2018.04.002
- Pagiola S, Arcenas A, Platais G (2005) Can Payments for Environmental Services help reduce poverty? An exploration of the issues and the evidence to date from Latin America. World Dev 33:237–253. doi: 10.1016/j.worlddev.2004.07.011
- Pardos M, del Río M, Pretzsch H, et al (2021) The greater resilience of mixed forests to drought mainly depends on their composition: Analysis along a climate gradient across Europe. For Ecol Manage 481:118687. doi: 10.1016/j.foreco.2020.118687
- Perera AH, Buse LJ, Crow TR (2006) Knowledge Transfer in Forest Landscape Ecology: A Primer. Springer New York
- Rametsteiner E, Eichler L, Berg J (2009) Shaping Forest Communication in the European Union: Public Perceptions of Forests and Forestry. ECORYS. Final Report
- Reed MS (2008) Stakeholder participation for environmental management: A literature review. Biol Conserv 141:2417–2431. doi: 10.1016/j.biocon.2008.07.014
- Ritter E, Dauksta D (2013) Human-forest relationships: Ancient values in modern perspectives. Environ Dev Sustain 15:645–662. doi: 10.1007/s10668-012-9398-9
- Rodríguez-Morales B, Roces-Díaz J V., Kelemen E, et al (2020) Perception of ecosystem services and disservices on a peri-urban communal forest: Are landowners’ and visitors’ perspectives dissimilar? Ecosyst Serv 43:101089. doi: 10.1016/j.ecoser.2020.101089
- Scholte SSK, van Teeffelen AJA, Verburg PH (2015) Integrating socio-cultural perspectives into ecosystem service valuation: A review of concepts and methods. Ecol Econ 114:67–78. doi: 10.1016/J.ECOLECON.2015.03.007
- Secco L, Paletto A, Romano R, et al (2018) Orchestrating Forest Policy in Italy: Mission Impossible? Forests 9:468. doi: 10.3390/f9080468
- Torralba M, Lovrić M, Roux JL, et al (2020) Examining the relevance of cultural ecosystem services in forest management in Europe. Ecol Soc 25:2. doi: 10.5751/ES-11587-250302
- Van Hecken G, Bastiaensen J, Windey C (2015) Towards a power-sensitive and socially-informed analysis of payments for ecosystem services (PES): Addressing the gaps in the current debate. Ecol Econ 120:117–125. doi: 10.1016/j.ecolecon.2015.10.012
- Verkerk PJ, Costanza R, Hetemäki L, et al (2020) Climate-Smart Forestry: the missing link. For. Policy Econ. 115:102164
- Whatmore S (2006) Materialist returns: Practising cultural geography in and for a more-than-human world. Cult Geogr 13:600–609. doi: 10.1191/1474474006cgj377oa
- Wolff S, Schulp CJE, Verburg PH (2015) Mapping ecosystem services demand: A review of current research and future perspectives. Ecol Indic 55:159–171. doi: 10.1016/J.ECOLIND.2015.03.016
- Wunder S, Börner J, Ezzine-de-Blas D, et al (2020) Payments for Environmental Services: Past Performance and Pending Potentials. Annu Rev Resour Econ 12:209–234. doi: 10.1146/annurev-resource-100518-094206
- Wunder S, Brouwer R, Engel S, et al (2018) From principles to practice in paying for nature’s services. Nat Sustain 1:145–150. doi: 10.1038/s41893-018-0036-x
- Wunder S, Feder S, Pettenella D, et al (2019) “What works?” State-of-the-art synthesis report about best-practice design and implementation of PES and other IM in the European context. SINCERE project. Deliverable 1.4
